# Recent synthetic strategies of spiro-azetidin-2-one, -pyrrolidine, -indol(one) and -pyran derivatives-a review

**DOI:** 10.1039/d3ra06054c

**Published:** 2023-11-07

**Authors:** Mohammed B. Alshammari, Ashraf A. Aly, Akil Ahmad, Alan B. Brown, Asmaa H. Mohamed

**Affiliations:** a Chemistry Department, College of Sciences and Humanities, Prince Sattam Bin Abdulaziz University Al-Kharij Saudi Arabia m.alshammari@psau.edu.sa aj.ahmad@psau.edu.sa; b Chemistry Department, Faculty of Science, Minia University 61519 El-Minia Egypt ashrafaly63@yahoo.com ashraf.shehata@mu.edu.eg asmaa.hamouda@mu.edu.eg; c Department of Chemistry and Chemical Engineering, Florida Institute of Technology Melbourne FL 32901 USA abrown@fit.edu

## Abstract

Spiro-heterocycles have received special attention in medicinal chemistry because of their promising biological activity. Over the years, many synthetic methodologies have been established for the construction of spirocyclic compounds. Spiro heterocycles such as spiro-azetidin-2-one, -pyrrolidine, -indol(one) and -pyran derivatives have been found to exhibit diversified biological and pharmacological activity in addition to their therapeutic properties. In view of these facts, we decided in this review to present representative synthetic approaches of the aforementioned spiro heterocycles, especially in the past 20 years.

## Abstract 2

Spiro-heterocycles have received special attention in medicinal chemistry because of their promising biological activity.

## Graphical abstract

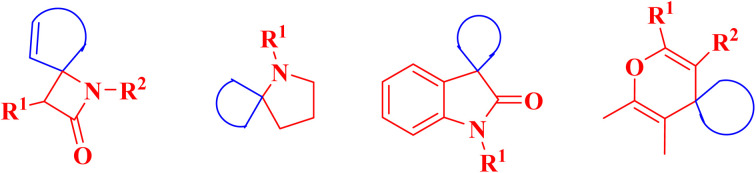

## 1. Introduction

Spirocyclic compounds are characterized by having two rings sharing the same atom, the quaternary spiro carbon.^1–3^ The inherent rigidity of spirocyclic compounds causes a decrease in the conformational entropy penalty when it comes to an interaction between a potential bioactive spiro compound and its putative molecular target.^1,4–7^ Spiro compounds are considered spiro heterocyclic if the spiro atom or any atom in either ring are not carbon atoms. Spiro heteroatoms such as nitrogen, oxygen, or sulfur connecting the rings have been commonly observed. Moreover, there are also many classes where one or more heteroatoms appear in one or more of the rings that are joined at a carbon spiro atom. In this review, we focus on four classes of important spiro heterocycles identified as spiro-azetidin-2-one, -pyrrolidine, -indol(one) and -pyran derivatives.

### 1.1. Spiro-azetidine-2-one derivatives

**Fig. 1:**
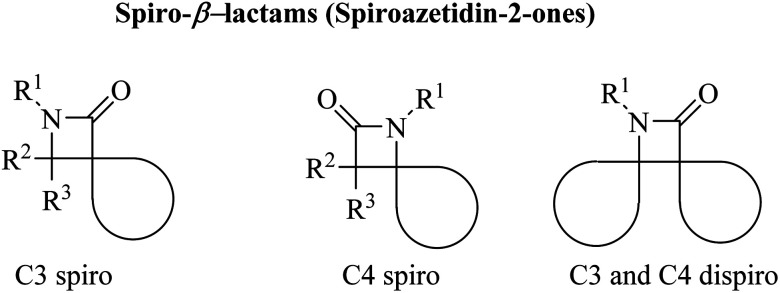
Different structures of spiroazetidin-2-ones.

Azetidine can be considered as a fairly typical cyclic amine. Strain in the four-membered ring is less than that in the three-membered aziridine system; as a result azetidines show few of the exceptional properties associated with aziridines. In spirocyclic *β*-lactams, the spiro carbon may be at positions C3 and/or C4 (Fig. 1).

**Fig. 2:**
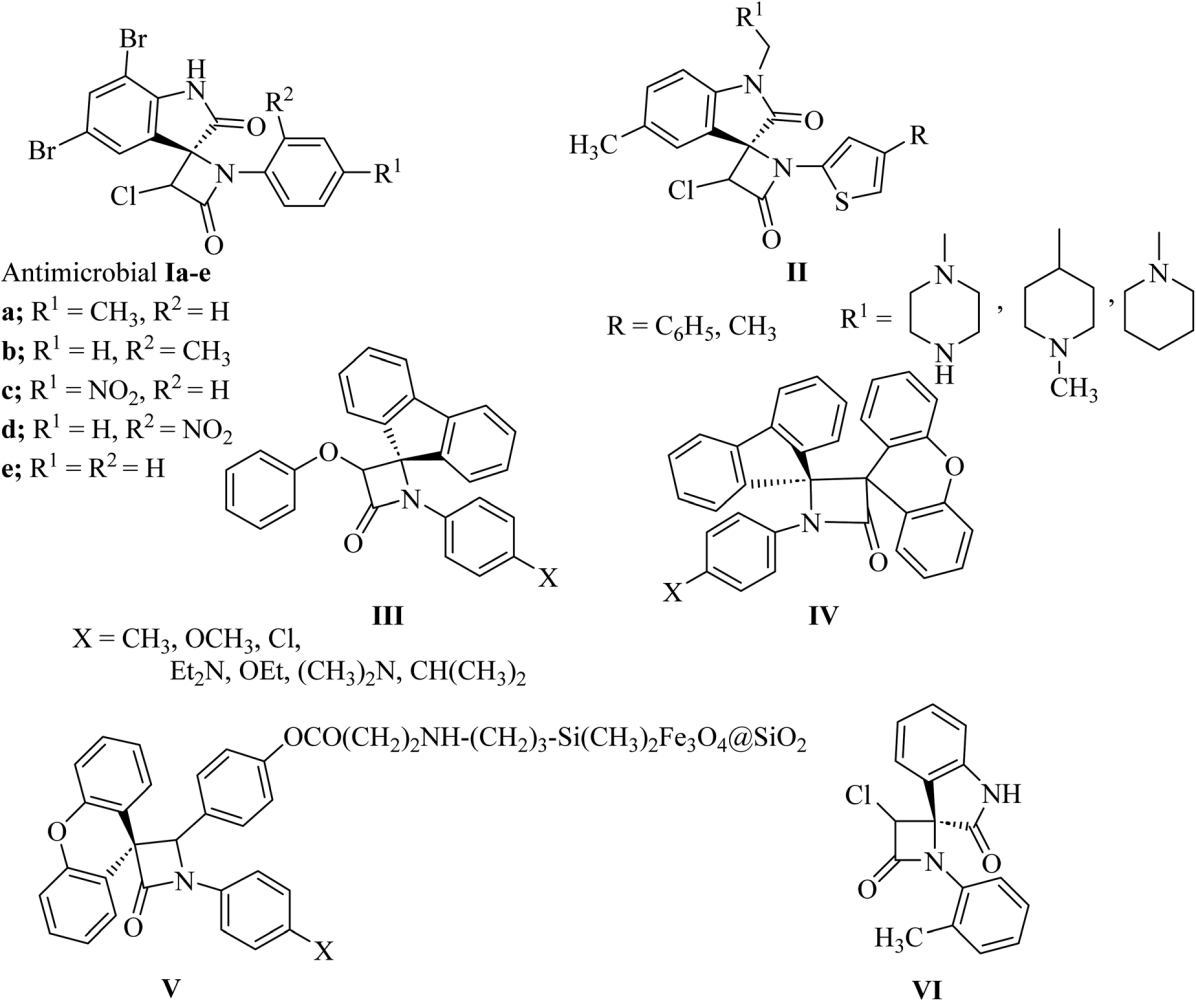
Biologically active spiro azetidin-2-one derivatives I–VI.

The azetidin-2-one ring has given life-saving penicillin and cephalosporin antibiotics.^8^ Further exploitation of the *β*-lactam ring has yielded biologically active new chemical entities exhibiting a variety of activities.^8^ Over the years, *β*-lactams have also emerged as versatile building blocks (*β*-lactam synthon method) for the synthesis of amino acids, alkaloids and toxoids with potential biological properties. Interestingly, spiro[azetidine-2,3′-indole]-2′,4(1′*H*)-dione derivatives Ia–e (Fig. 2) showed antibacterial and antifungal activities.^9^ The antibacterial activity of all the compounds against *Staphylococcus aureus* as Gram-positive, *Escherichia coli* and *Pseudomonas aeruginosa* as Gram-negative bacteria showed good potencies which are comparable to control drugs amoxicillin, gentamycin, and streptomycin. With bromo substituents at the 5′ and 7′ positions of indoline, Ia–e showed very good activity with MIC values of 6.25–12.5 μg mL^−1^. Other derivatives have exhibited moderate activity against all three bacterial strains. Similar structure II (Fig. 2) have shown anthelmintic potency and were evaluated *versus* standard albendazole. Antibacterial activity was also tested for the synthesized compounds with standard ampicillin against the five different pathogens *Bacillus subtilis*, *Pseudomonas*, *aeruginosa*, *Escherichia coli*, *Proteus mirabilis* and *Staphylococcus aureus*.^10^ Spiro-*β*-lactams III and dispiro-*β*-lactams IV (Fig. 2) have exhibited good to excellent antimalarial activities against chloroquine-resistant *Plasmodium falciparum* K14 strain with IC_50_ varying from 5 to 32.2 mM.^11^ The spirocyclic *β*-lactam V (Fig. 2), supported on superparamagnetic Fe_3_O_4_@SiO_2_ nano-particles, enhanced the antibacterial activity in comparison with the corresponding spirocyclic *β*-lactam, which was supposed to be due to the synergic effect of the Fe_3_O_4_@SiO_2_/*β*-lactam combination.^12,13^ A number of spiro-azetidine-dione derivatives have been tested for anti-breast cancer activity, however, only 3-chloro-1-(*o*-tolyl)spiro[azetidine-2,3′-indoline]-2′,4-dione (VI) (Fig. 2) displayed a significant cytotoxicity (IC_50_ = 22.75–25.18 μM) for breast cancer cell lines, which was comparable to the standard control drug doxorubicin.^14^

**Fig. 3:**
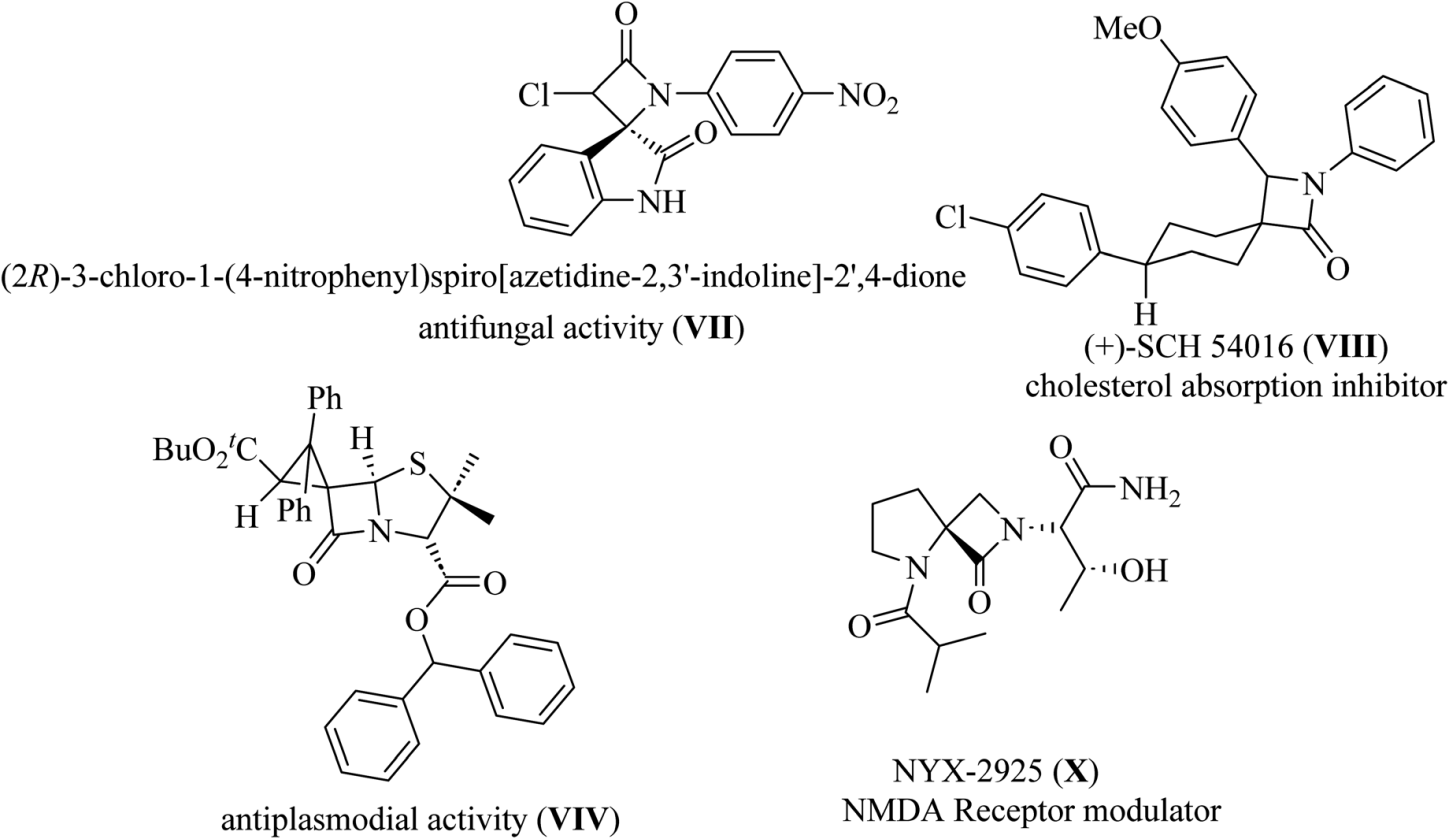
Representative examples of biologically active spiro cyclic *β*-lactam VII–X.

Spiro-lactams include synthetic biologically active molecules such as antifungal spirooxindole *β*-lactam VII,^12^ cholesterol absorption inhibitor (+)-SCH 54016 VIII,^15^ antiplasmodial spiro penicillate VIV,^16–18^ and NMDA receptor modulator NYX-2925 X ^19^ (Fig. 3).

### 1.2. Spiro-pyrrolidine derivatives

**Fig. 4:**
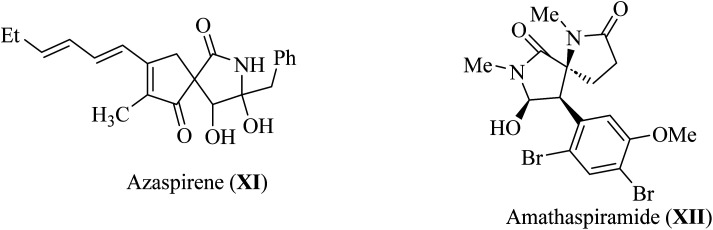
Alkaloids naturally occurring pyrroldin-2-ones XI–XII.

Pyrrolidine fragments are widespread in nature; in particular, they are structural units of numerous biologically active compounds^20,21^ and natural alkaloid acting as an efficient glucosidase I inhibitors used in the therapy of type II diabetes. Such as azaspirene,^22^ and casuarina^23^ (Fig. 4). Some synthetic spiropyrrolidines were found to be promising antileukemic agents and anticonvulsants.^24–27^ In addition, spiro pyrrolidine-2-ones such as Azaspirene (XI) are found in Nature.^28^ The marine alkaloid amathaspiramide XII (Fig. 4), isolated from Zealand collection of the marine bryozoan *Amathia wilsoni*, shows potential antiviral, antimicrobial and cytotoxic activities.^29^

### 1.3. Spiro-indol(one) derivatives

**Fig. 5:**
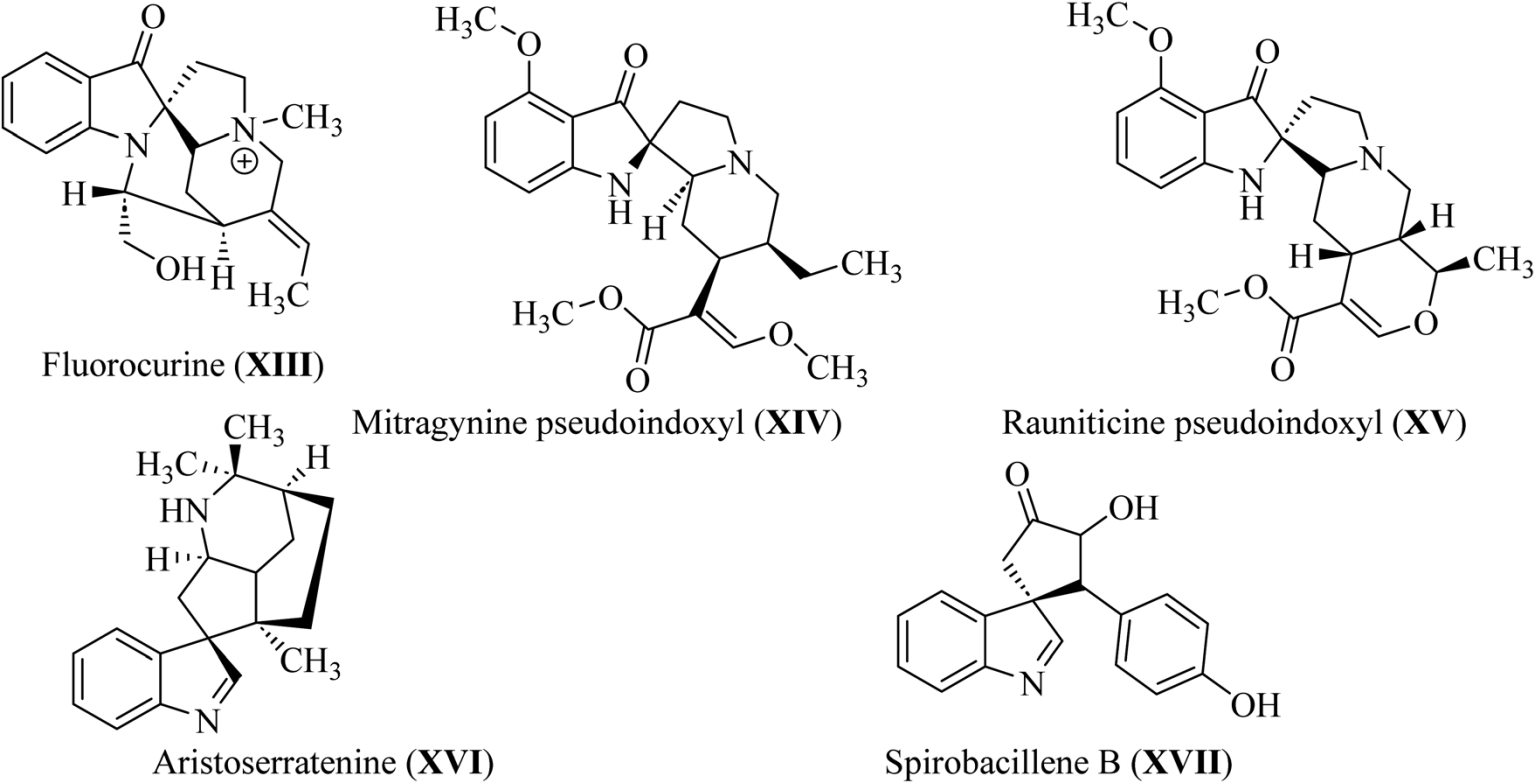
Natural C-2 and C-3 spiroindol and spiroindol(one) containing compounds XIII–XVII.

In spiroindol(one)s, an indolone ring is substituted with another ring in a spiro arrangement. Spiro indol(one) derivatives occupy a unique place within organic chemical compounds due to their rigidity and 3D-geometrical structure.^30^ These structural characteristics give rise to the versatile biological properties shown by analogs wherein C-2 or C-3 of the indolyl ring is spiro-cyclized with many heterocycles (XIII–XVII; Fig. 5).^31^

**Fig. 6:**
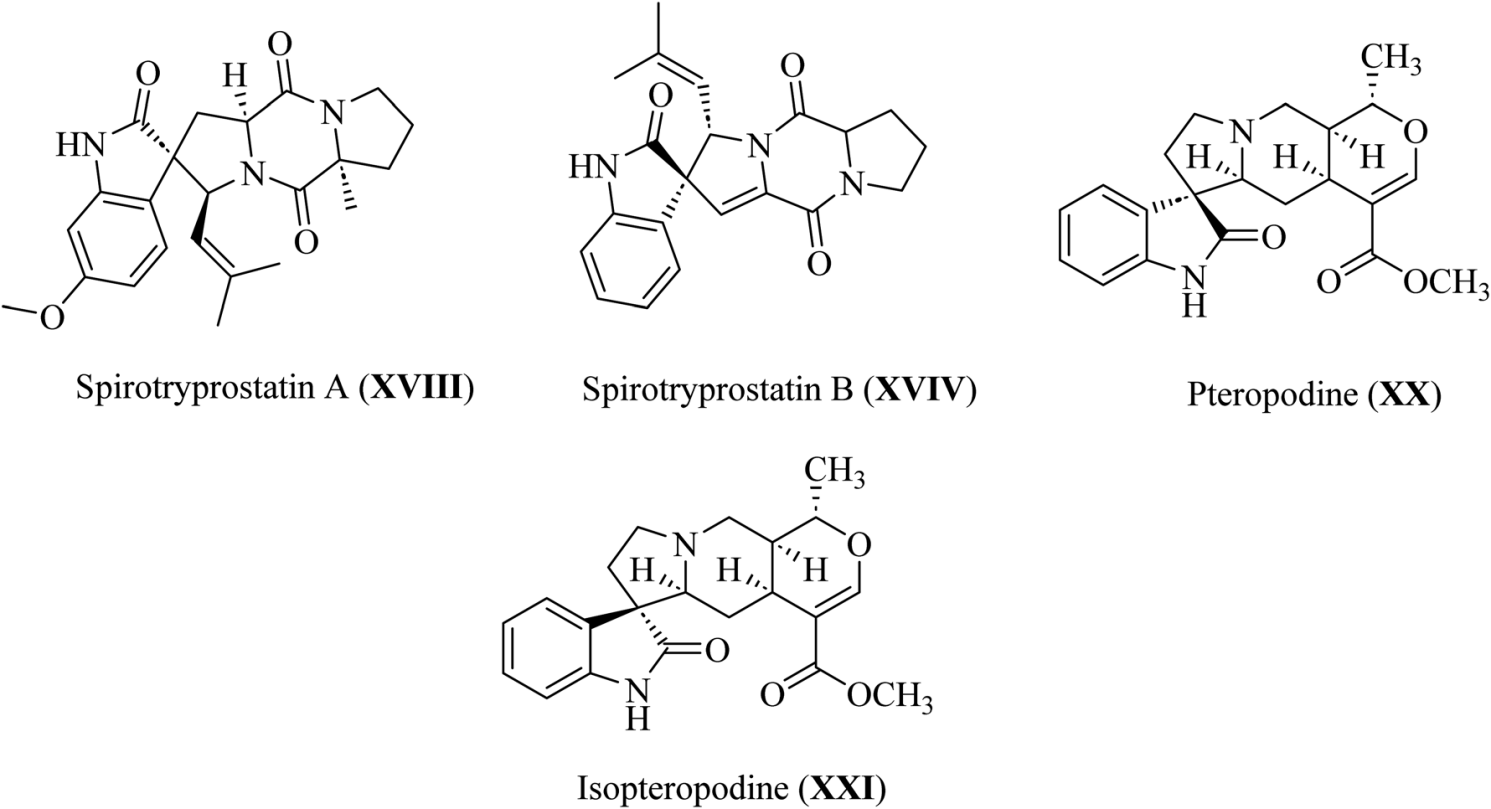
Selected pharmaceutical structures containing spiro indol(one) scaffolds XVIII–XXI.

Spirotryprostatin A (XVIII) and spirotryprostatin B (XVIV) showed microtubule assembly inhibition, whereas pteropodine (XX) and isopteropodine (XXI) damped the operation of muscarinic serotonin receptors (Fig. 6).^32,33^

### 1.4. Spiro-pyran derivatives

Spiropyrans were discovered in the early twentieth century.^34^ In the 1920s, Fisher and Hirshbergin observed their photochromic characteristics and reversible reaction.^34^ Studies on photochromic compounds that have continued up to the present.^35,36^

Spiropyran and spirooxazine compounds can undergo reversible structural transformations under the influence of external stimuli; this induces a color change, as well as changes in their physical and chemical properties.^37,38^ Spiropyrans and spirooxazines are the most investigated photochromic spiro compounds.^39,40^ It is reported that spiropyrans and spirooxazines could also respond to other external stimuli such as thermal effects, pH, and stress.^41–43^

**Fig. 7:**
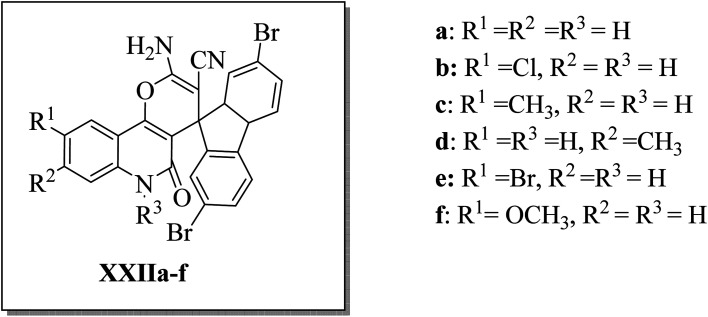
2′-Aminospiro[pyrano[3,2-*c*]quinoline]-3′-carbonitrile derivatives XXIIa–f as ATP-non-competitive Src inhibitors that suppress breast cancer cell migration and proliferation.

Aly *et al.*^44^ designed and synthesized three series of 2′-aminospiro[pyrano[3,2-*c*]quinoline]-3′-carbonitrile derivatives XXIIa–f (Fig. 7), hypothesizing that small molecules with a spiro scaffold appended to a pyrano[3,2-*c*]quinoline analog could act as ATP-noncompetitive Src kinase inhibitors. XXIIb, XXIIc, and XXIId inhibited Src kinase activity with IC_50_'s of 4.9, 5.9, and 0.9 μM, respectively. At the same time, they did not affect the MDM2/p53 interaction in HEK293 cells that have been reported to be affected by some spirocyclic compounds. Kinetic analysis for the inhibition of Srctide phosphorylation by XXIId revealed a mechanism of ATP-non-competitive inhibition. 1 μM of XXIId was enough to diminish Src, Fak, and paxillin phosphorylation in the MCF7 breast cancer cell line.^44^

This work is divided into four distinct classes of spiro heterocycles: spiro-azetidin-2-one, -pyrrolidine, -indol(one) or -pyran derivatives. We deal with building the aforementioned spiro heterocycles, according to the method's scope, selectivity, and reaction mechanism. Previously, a few reviews dealt with the syntheses of these types of spiro heterocycles;^45–47^ however, each review article dealt with only one of the four classes mentioned. We concentrate on the synthesis of these spiro compounds in the past 20 years, and discuss the biological activity of some of these classes.

## 2. Discussion

### 2.1. Synthesis of spiro-azetidin-2-one derivatives

**Scheme 1:**
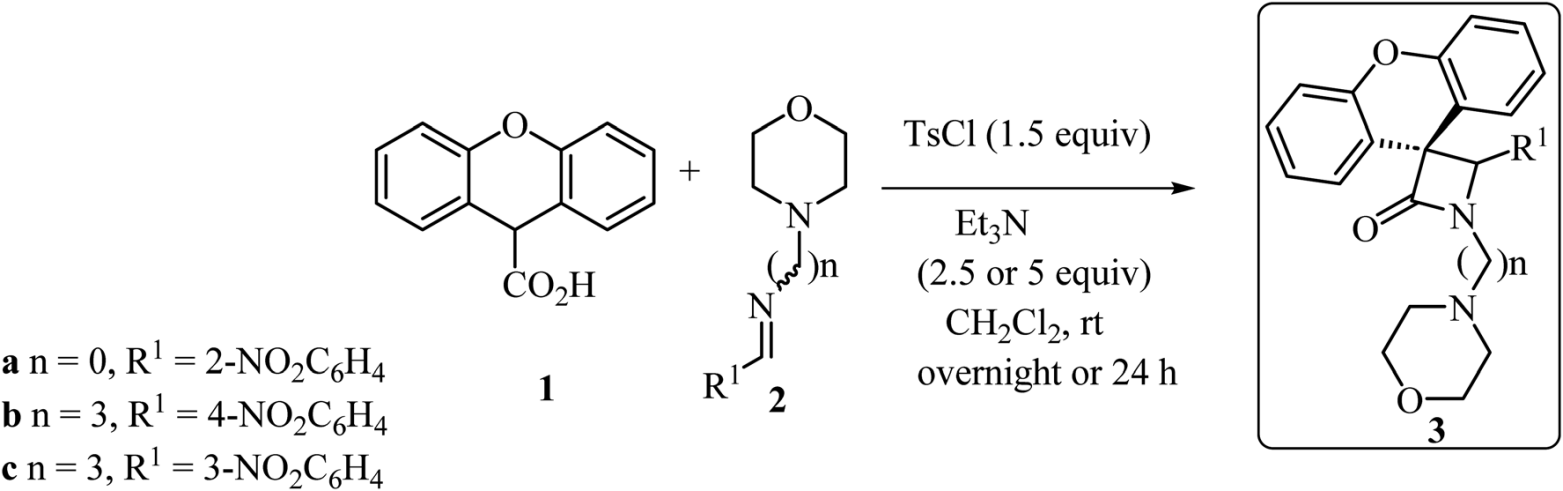
Staudinger synthesis of spiro-*β*-lactams 3 containing the xanthene moiety.

With the discovery and structural elucidation of the antibiotic penicillin, the Staudinger synthesis became of major importance in medicinal chemistry, as it allowed the synthesis of penicillin derivatives in the laboratory. Although several alternative methods have been developed, the Staudinger reaction remains the most common method for the synthesis of *β*-lactams, including spiro-*β*-lactams.^48^ Heiran and co-workers synthesized C3 spiro-*β*-lactams 3 bearing a morpholine ring, in moderate to good yields (41–71%) (Scheme 1)^49^*via* cyclocondensation of xanthene-9-carboxylic acid (1) and aromatic imines in the presence of tosyl chloride (TsCl) and triethylamine (Et_3_N) in dichloromethane (CH_2_Cl_2_).

**Scheme 2:**
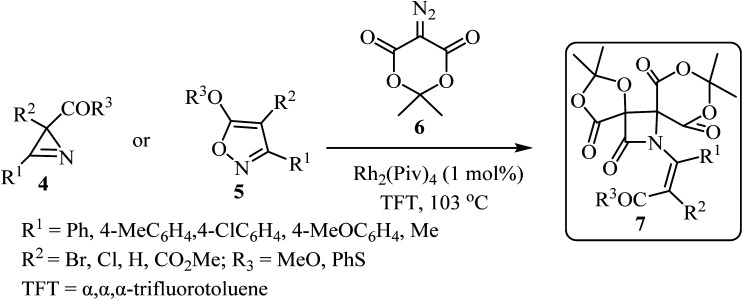
Synthesis of dispirocyclic *N*-vinyl-*β*-lactam 7 from diazo Meldrum's acids (6) and 2*H*-azirines or 5-alkoxyisooxazoles 5.

In 2019, Novikov and co-workers^50^ reported the domino synthesis of dispirocyclic *N*-vinyl *β*-lactams 7 from diazo-Meldrum's acid (6) and 2*H*-azirines 4 or 5-alkoxyisoxazoles 5 through Rh_2_(Piv)_4_-catalyzed 2-azabuta-1,3-diene formation, and subsequent Staudinger ketene-imine cycloaddition (Scheme 2).^50^ The reaction was carried out in trifluorotoluene (TFT).

**Scheme 3:**
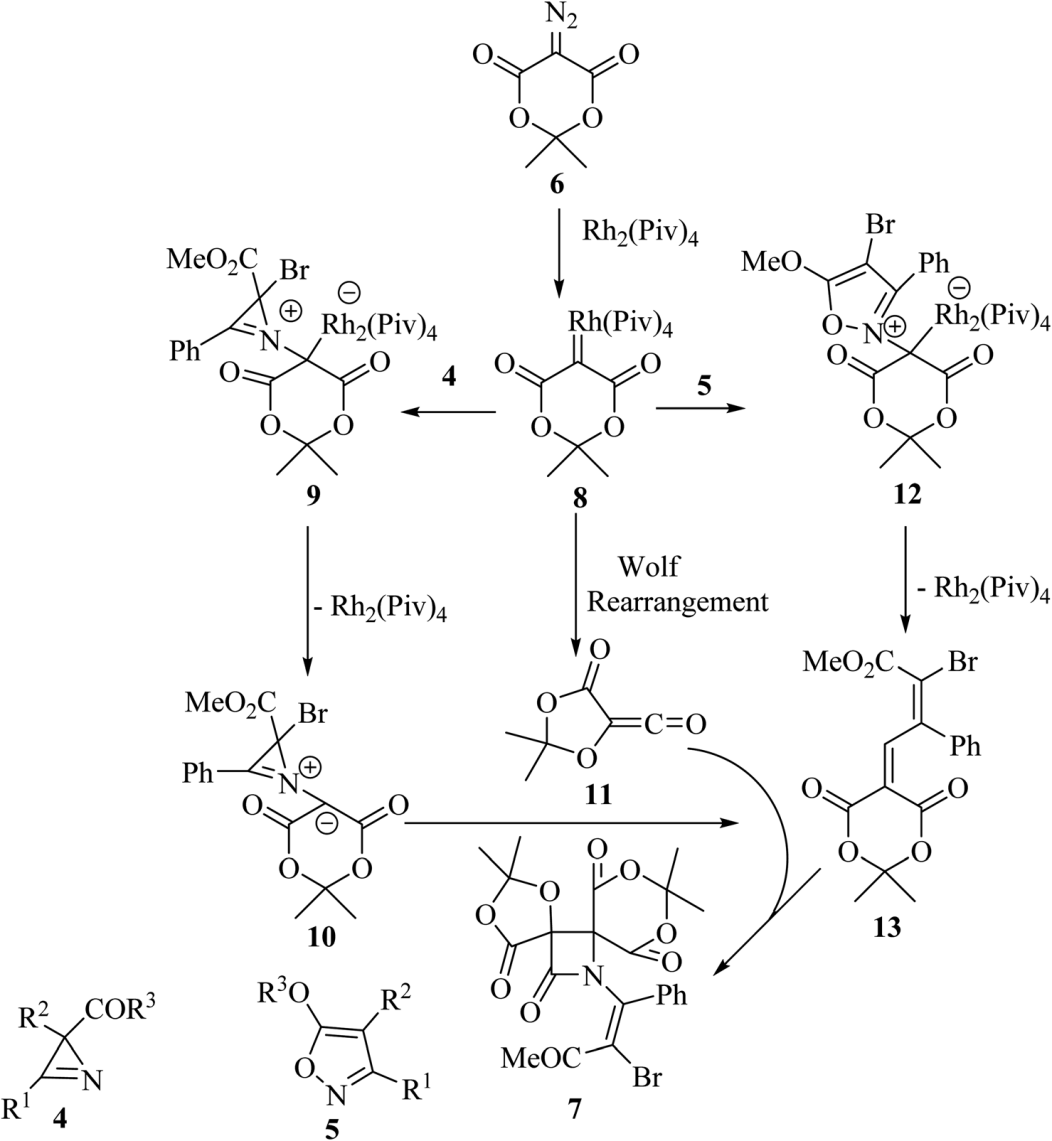
Mechanism described the formation of compound 7.

Concerning the reaction mechanism, the rhodium carbenoid 8 obtained from the Meldrum's acid derived diazo-compound 6, adds to the azirines 4 or isoxazoles 5 forming adducts 9 or 12, respectively (Scheme 3). Both pathways lead to the same 2-azabuta-1,3-diene 13 product. A parallel Meldrum's acid carbenoid Wolff rearrangement leads to the *in situ* generation of a ketene 11, which undergoes a [2 + 2] Staudinger cycloaddition with 13 to give *β*-lactam 7. By using different substituent groups on both azirine and isoxazole, nine different dispirocyclic *N*-vinyl *β*-lactams 7 were obtained in low to moderate yields (22–67%).^50^

**Scheme 4:**
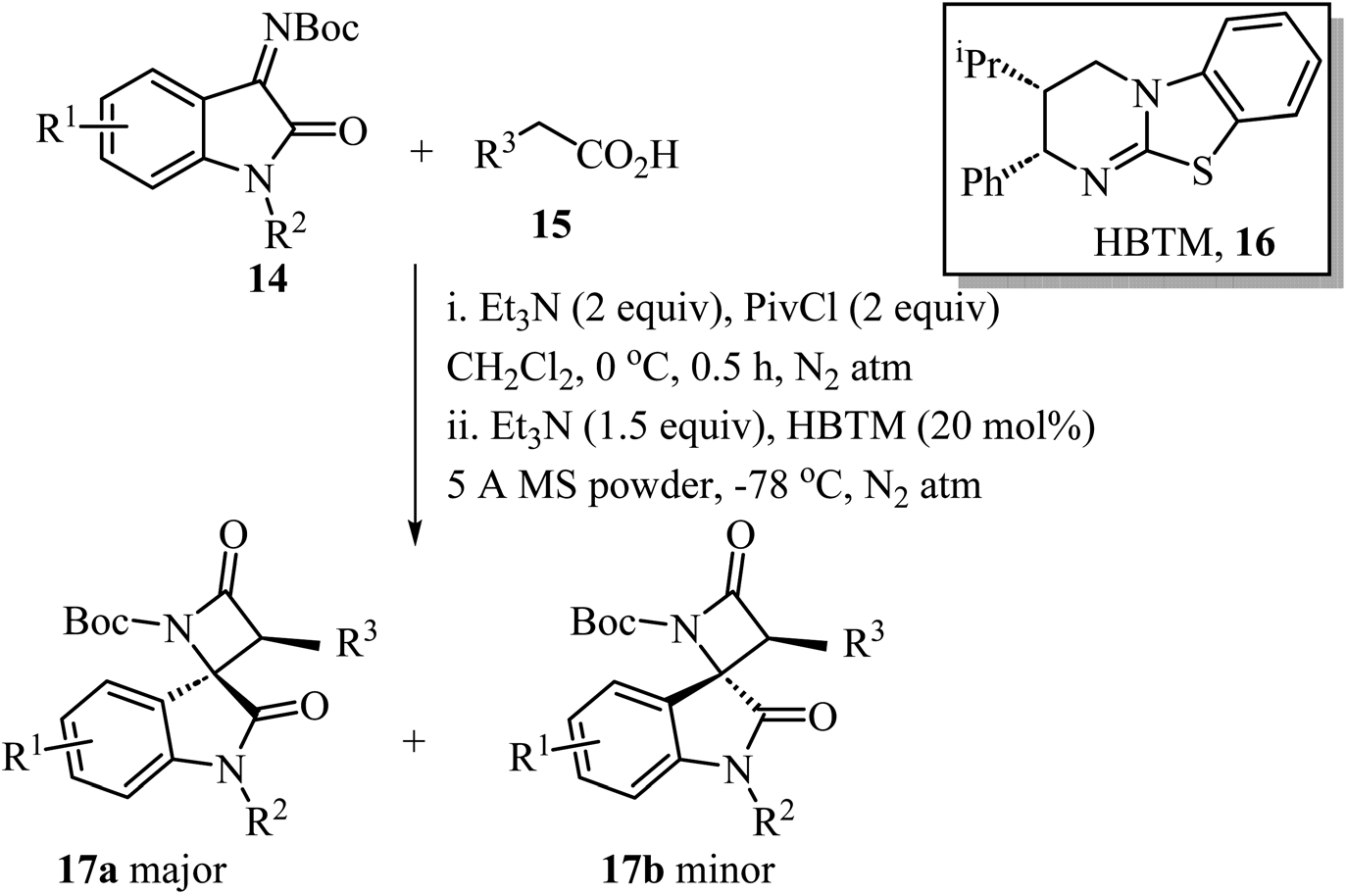
HBTM catalyzed Mannich/lactamization cascade reaction of isatin derived imine 14 with aryl acetic acids 15.

A convenient procedure reported by Zhao, Deng and co-workers led to the synthesis of twenty-two enantio-enriched spirooxindole-*β*-lactams 17 bearing two vicinal stereogenic centers.^51^ The molecules were obtained in high yields (up to 98%), with good to high diastereo-selectivity and excellent enantioselectivities (Scheme 4). The reaction happens through a homo benzotetramisole (HBTM)-catalyzed Mannich/lactamization cascade reaction of isatin-derived imines 14 with aryl acetic acids 15 (Scheme 4).^51^

**Scheme 5:**
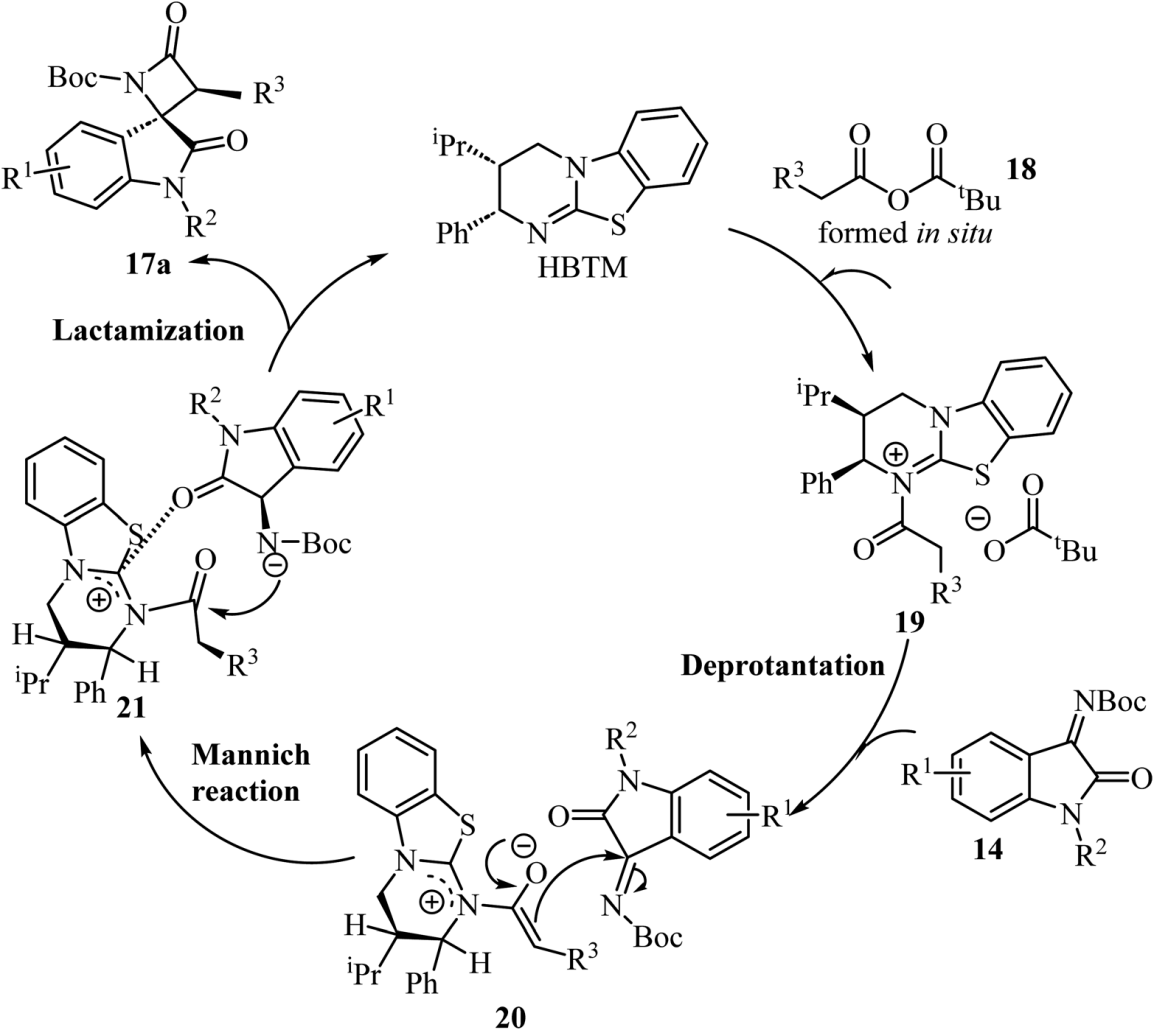
A plausible mechanism for the formation of 17a.

Concerning the proposed reaction mechanism,^51^ the first step of the reaction involves a reaction between the aryl acetic acid 15 and pivaloyl chloride which generates a mixed anhydride 18, responsible for the HBTM acylation which afforded intermediate 19 (Scheme 5). Deprotonation of intermediate 19 occurs on the C1-ammonium enolate to give intermediate 20, which subsequent *Si*-face-attack Mannich reaction afforded intermediate 21. On the last step of the catalytic cycle, intermediate 21 underwent an intramolecular lactamization providing the desired *cis*-spiro-oxindole *β*-lactam product 17a and regenerating the HBTM catalyst (Scheme 5).

**Scheme 6:**
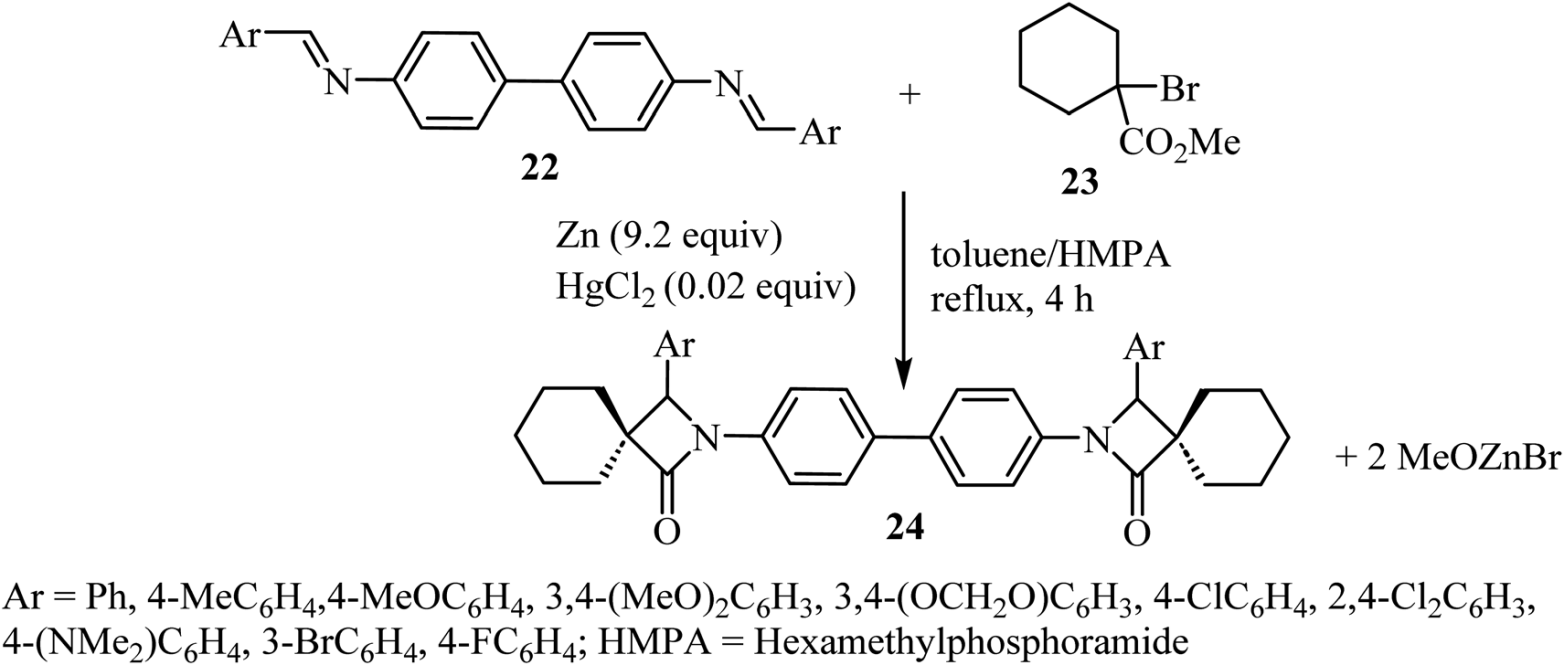
Zinc mediated reaction between *N*,*N*-bis(arylmethylidene)benzidines 22 and a *α*-bromoalkancarboxylate 23.

Kirillov *et al.* synthesized a library of eleven bis(spiro-*β*-lactams) 24, using the Reformatsky reaction between methyl-1-bromo-cyclohexanecarboxylate (23) and *N*,*N*-bis-(arylmethylidene)benzidines 22 as the first step (Scheme 6).^52^

**Scheme 7:**
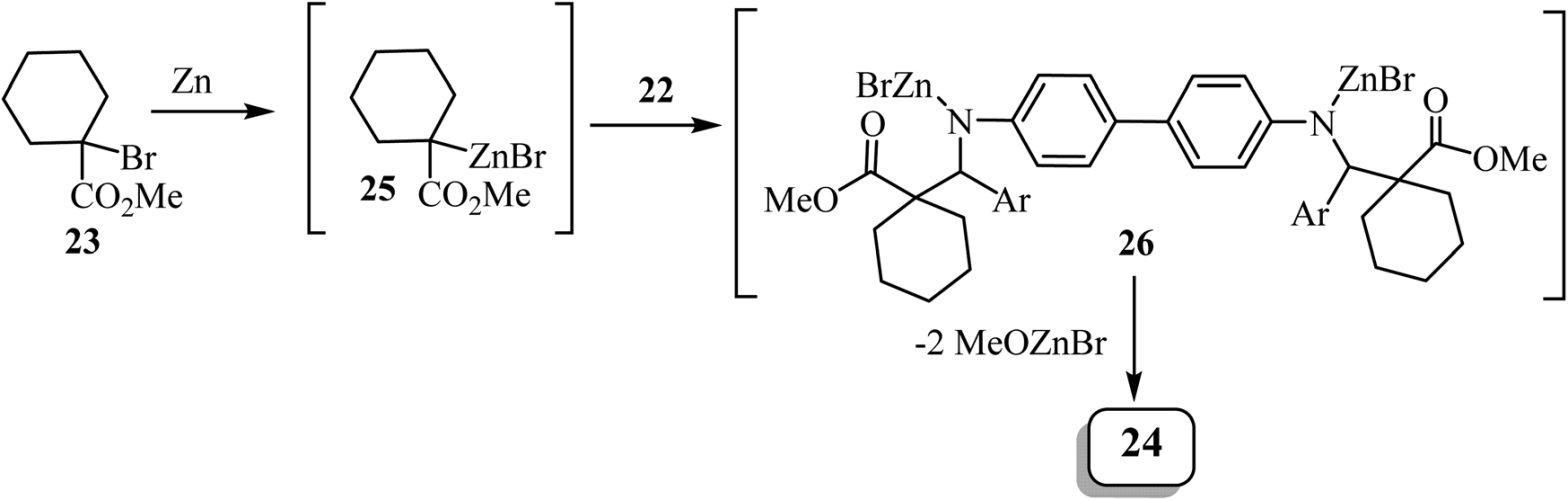
Rationale for the formation of compound 24.

Zinc metal would presumably react with methyl 1-bromocyclohexanecarboxylate to form Reformatsky reagent 25, which would react with the bis-imine to form the corresponding adduct 26 (Scheme 7). The adduct spontaneously cyclizes and forms the lactam ring, affording the bis-spiro-cyclohexane-*β*-lactam 24 ^52^ with elimination of bromo-zinc methoxide. Bis(spiro-*β*-lactams) 24 were isolated in good yields (69–84%) (Scheme 7). The reaction was also expanded to synthesize nine spiro-cyclopentane-containing *β*-lactams by using methyl 1-bromocyclo-pentanecarboxylate as the starting carboxylate, in yields ranging from 54% to 84%.

**Scheme 8:**
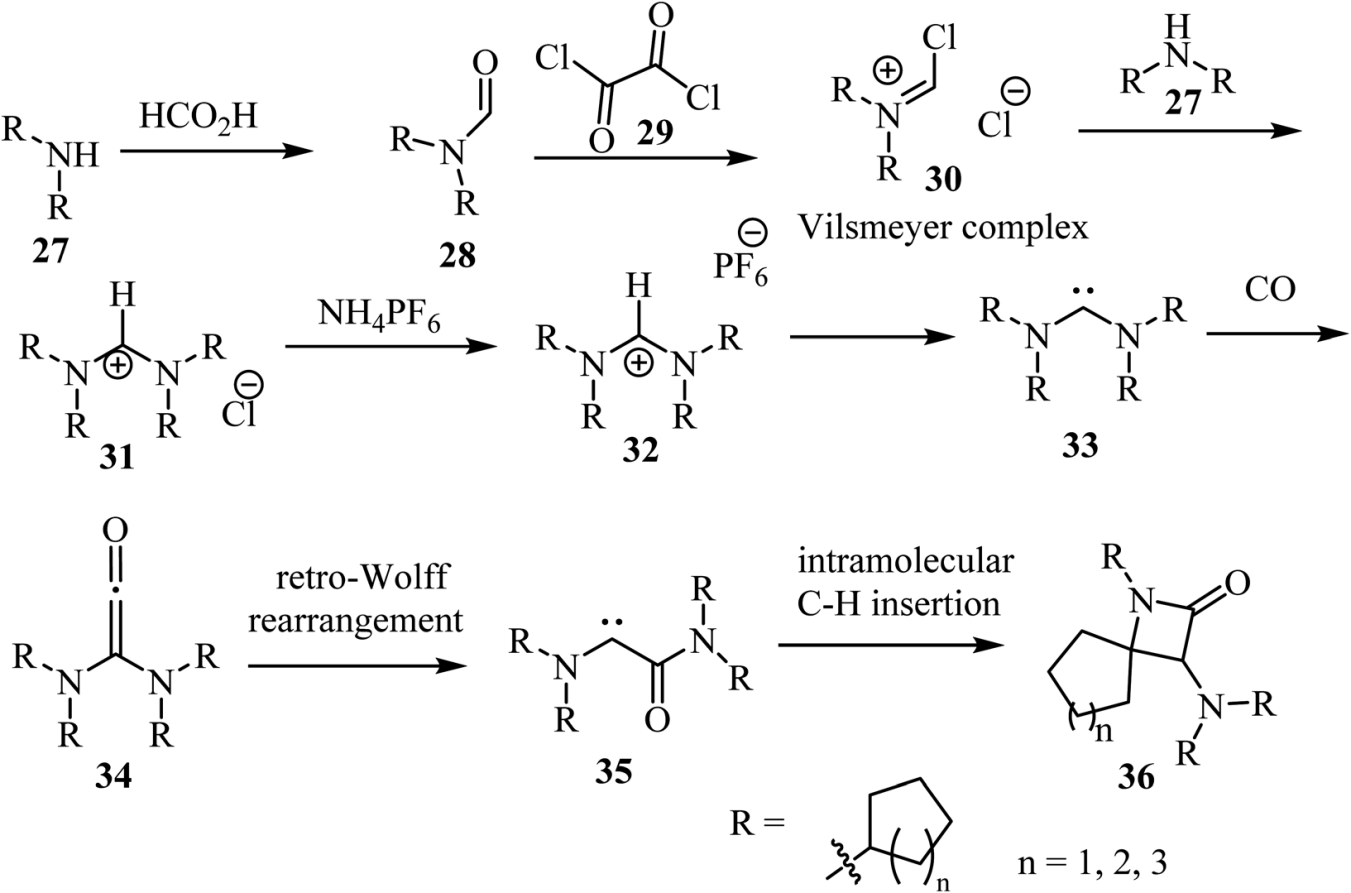
Carbonylation of acyclic diaminocarbenes leading to spiro *β*-lactams 36 containing cycloalkyl substituents.

Siemeling *et al.*,^53^ reported a metal-free synthesis of racemic spiro-*β*-lactam derivatives 36 by exploring the reactivity of acyclic diaminocarbenes 33, containing cycloalkyl substituents and using carbon monoxide as building block (Scheme 8). Compounds 33 were synthesized from secondary amines (*cyclo*-C_*n*_H_2*n*−1_)_2_NH (*n* = 5, 6, 7). The amines 27 were formylated with formic acid into compounds 28, which reacted with oxalyl (29) to give the corresponding Vilsmeier complex 30. The latter reacted with the secondary amines to afford formamidinium chlorides 31.^53^ Anion exchange was performed with ammonium hexafluorophosphate to afford the corresponding formamidinium hexafluorophosphates 32, which were converted into carbenes 33 upon treatment with NaN(SiMe_3_)_2_. The synthesis of the spirocyclic *β*-lactams 36 proceeded *via* carbonylation of the acyclic diaminocarbenes 33 leading to diaminoketenes 34, which underwent a retro-Wolff rearrangement to give (amido)(amino)carbenes 35 followed by an intramolecular C–H insertion to afford the final products 36 in yields ranging from 65% to 91% (Scheme 8).^53^

**Scheme 9:**
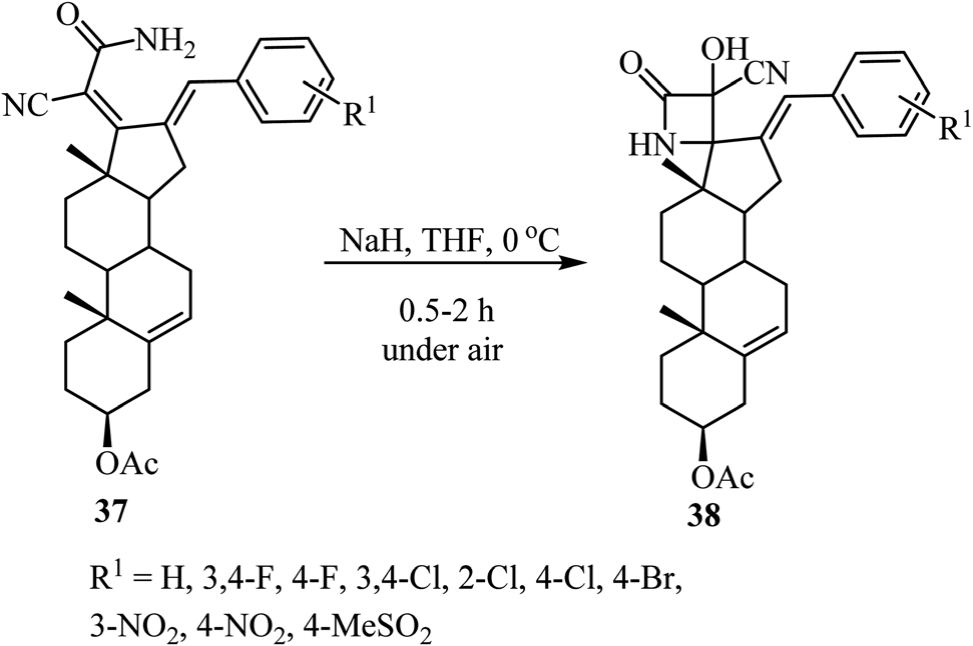
Steroidal spiro *β*-lactams 38; synthesis from dienamides 37 through a cascade 4-endo *N*-cyclization/aerobic oxidation sequence.

The synthesis of steroidal spiro-*β*-lactams 38, bearing a cyanohydrin functional group, from steroidal dienamides 37 has been reported (Scheme 9).^54^ The spirocyclic products were obtained in low to moderate yields (22–68%) under mild conditions and short reaction time in a one-pot procedure.^54^

**Scheme 10:**
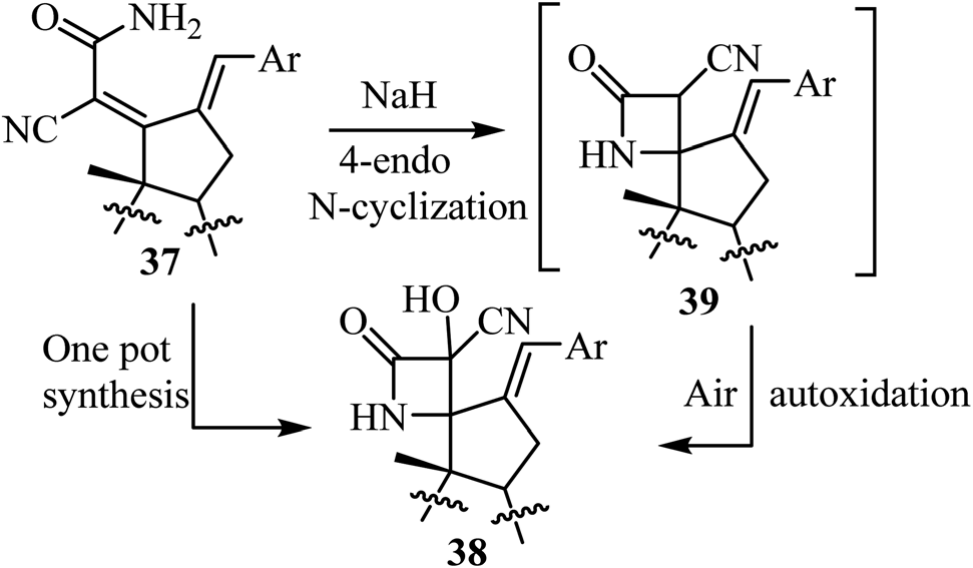
Plausible mechanism for the formation of compound 38.

The proposed mechanism involves an intramolecular lactamization of steroidal dienamides 37*via* a selective 4-*endo N*-cyclization, followed by a base-mediated aerobic oxidation which introduces a hydroxyl group at the *α*-position of the 2-azetidinone ring, generating the final spirocyclic product 38 ^54^ (Scheme 10).

**Scheme 11:**
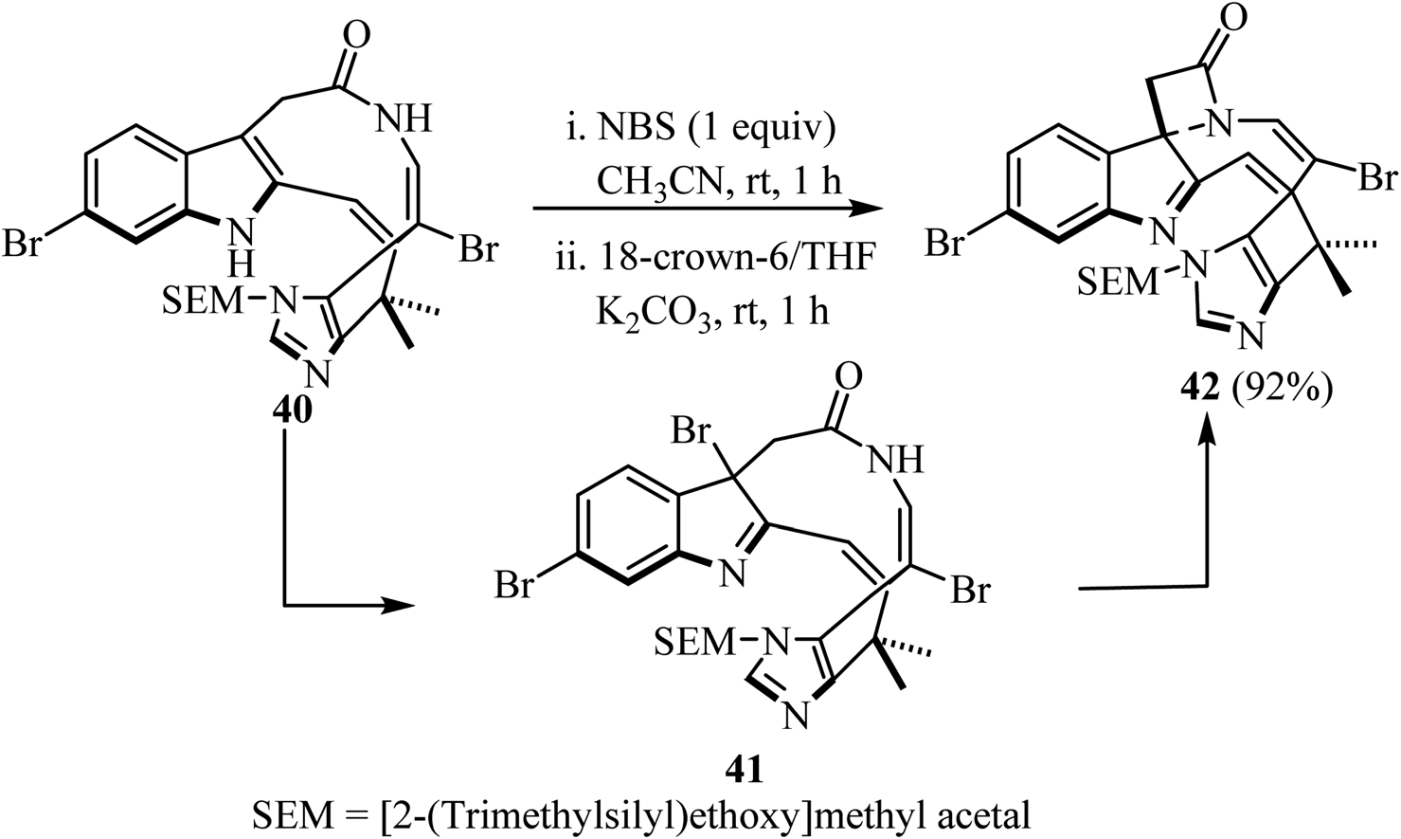
Chartelline-core spiroindolenine-*β*-lactam 42 synthesis *via* bromide mediated spirolactamization of a bromoenamide.

Recently, Nishikawa *et al.*^55^ disclosed the synthesis of a spiro-indolenine-*β*-lactam 42 analogue of alkaloid Chartelline C (Scheme 11). The two-step synthesis comprised the initial formation of a bromoindolenine intermediate 41*via* a *N*-bromosuccinimide (NBS)-mediated chemoselective bromination of bromoenamide 40 at C3, followed by intramolecular lactamization in the presence of 18-crown-6 and K_2_CO_3_/CH_3_CN. The target spirocyclic indolenine-*β*-lactam 42 was obtained in 92% yield.^55^

**Scheme 12:**
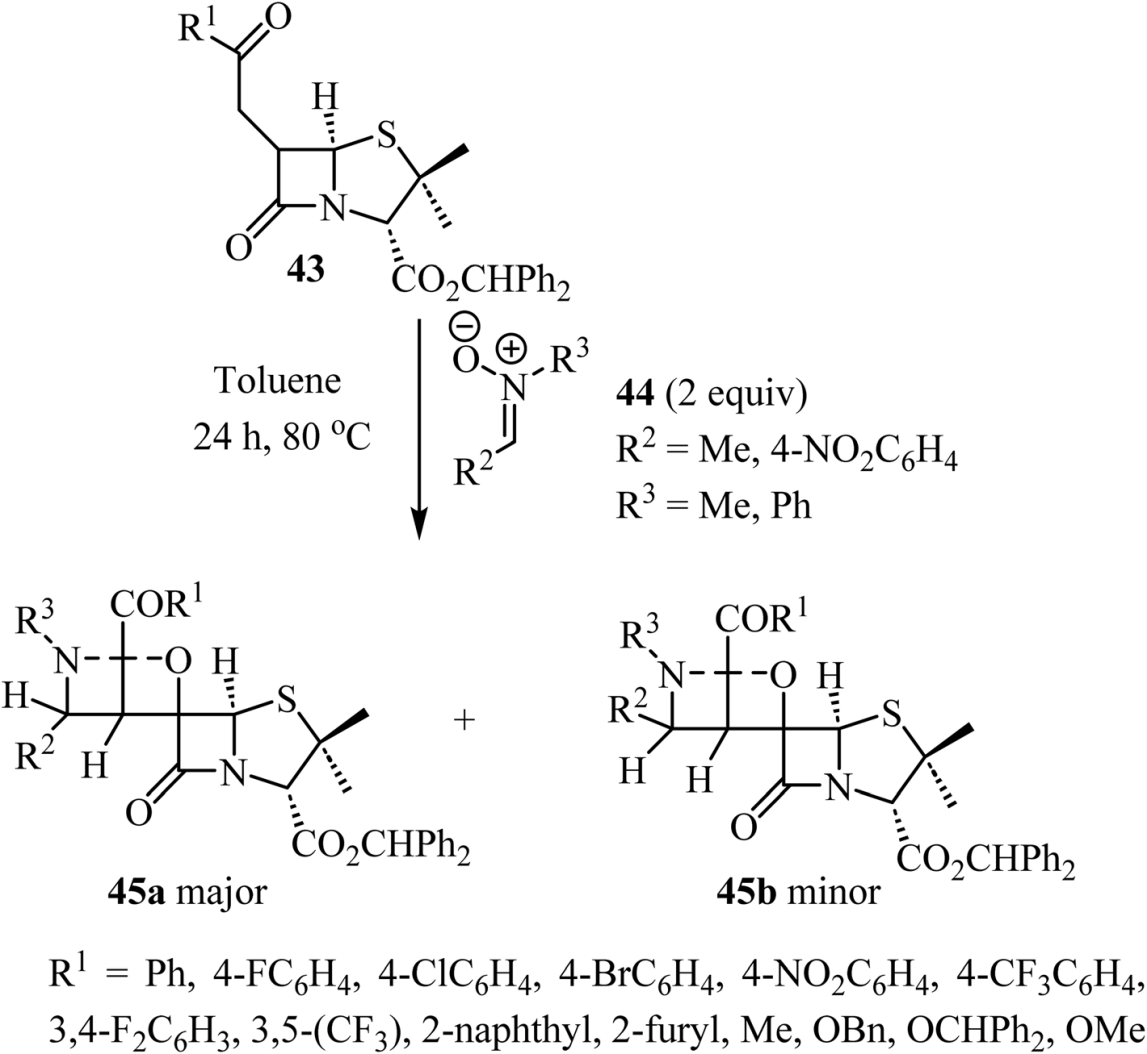
1,3-Dipolar cycloaddition between 6-alkylidene penicillanates 43 and nitrones 44.

The use of *C*-aryl-*N*-substituted nitrones 44 as dipoles in 1,3-dipolar cycloadditions with 6-alkylidene-penicillanates 43 to synthesize spiro-*β*-lactams was explored by Pinho e Melo *et al.* (Scheme 12).^56^ The generation of three consecutive stereogenic centers proved to be regio- and stereoselective and afforded chiral spiroisoxazolidine-penicillanates 45 in moderate to good overall yields, using mild conditions.^56^ The major products 45a were obtained through an *endo* 1,3-dipolar cycloaddition with addition of the nitrone to the *α*-side of the *β*-lactam and were obtained efficiently (26–80% yield); the stereoisomeric *exo*-cycloadducts 45b were isolated as minor products (7–27% yield). Two cases were stereospecific, only affording the major product 45a.

**Scheme 13:**
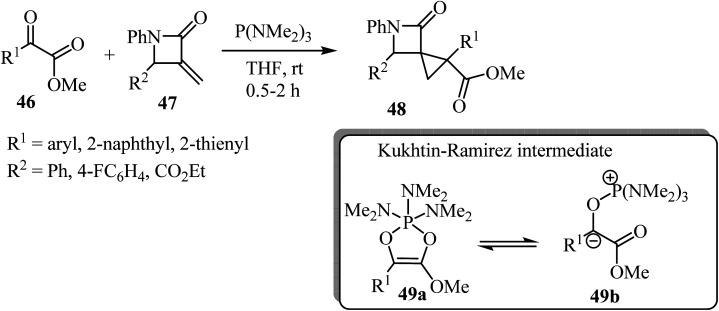
P(NMe_2_)_3_-mediated cyclopropanation of *α*-methylene-*β*-lactams 48.

Recently Luo *et al.* reported a phosphine-mediated reductive cyclopropanation reaction of *α*-keto esters 46 with *α*-methylene-*β*-lactams 47 (Scheme 13).^57^ That metal-free protocol provided the efficient *syn* synthesis of highly functionalized spirocyclopropyl *β*-lactams 48 through a mechanism involving the initial oxophilic addition of the phosphine to *α*-ketoester to generate Kukhtin-Ramirez intermediates (*e.g.* oxyphosphonium enolate 49b), which can behave as a carbene surrogate. Subsequent Michael addition of these intermediates to the electron deficient *β*-lactam exocyclic double bond followed by a 3-*exo-tet* cyclization furnishes spirocyclic lactams as diastereoisomeric mixtures.^57^

**Scheme 14:**
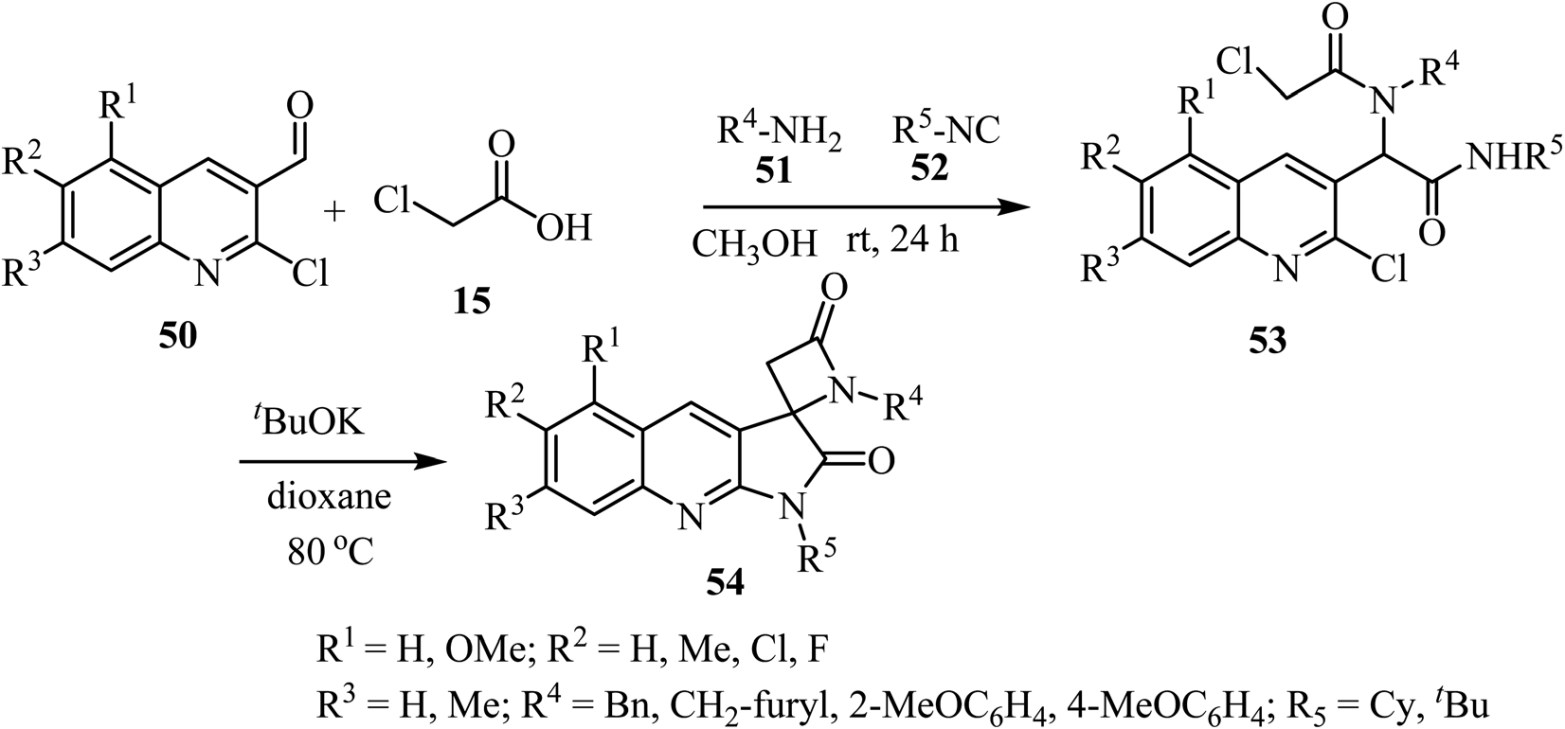
Synthesis of spirocyclic-bis-*β*-lactams-lactams 54 from four-component Ugi-adducts precursors.

The synthesis of a library of spiropyrrolo-quinoline *β*-lactams 54 was described, using four-component Ugi-adducts 53 as precursors (Scheme 14).^58^ These spirocyclic-bis-*β*-lactams 54 were obtained as racemic mixtures in moderate to high yields (54–88%). The Ugi-adducts 53 were synthesized through a four-component reaction of 2-chloro-3-formylquinolines 50, 2-chloroacetic acid (15), amines 51, and isocyanides 52 (Scheme 14). The proposed spirocyclization mechanism depends on two sequential cyclizations of the Ugi-adduct, under basic conditions. The first cyclization involves the *γ*-lactam ring formation *via* intramolecular aromatic nucleophilic substitution, followed by formation of the *β*-lactam ring through a nucleophilic acyl substitution.^58^

### 2.2. Synthesis of spiro pyrrolidine derivatives

**Scheme 15:**
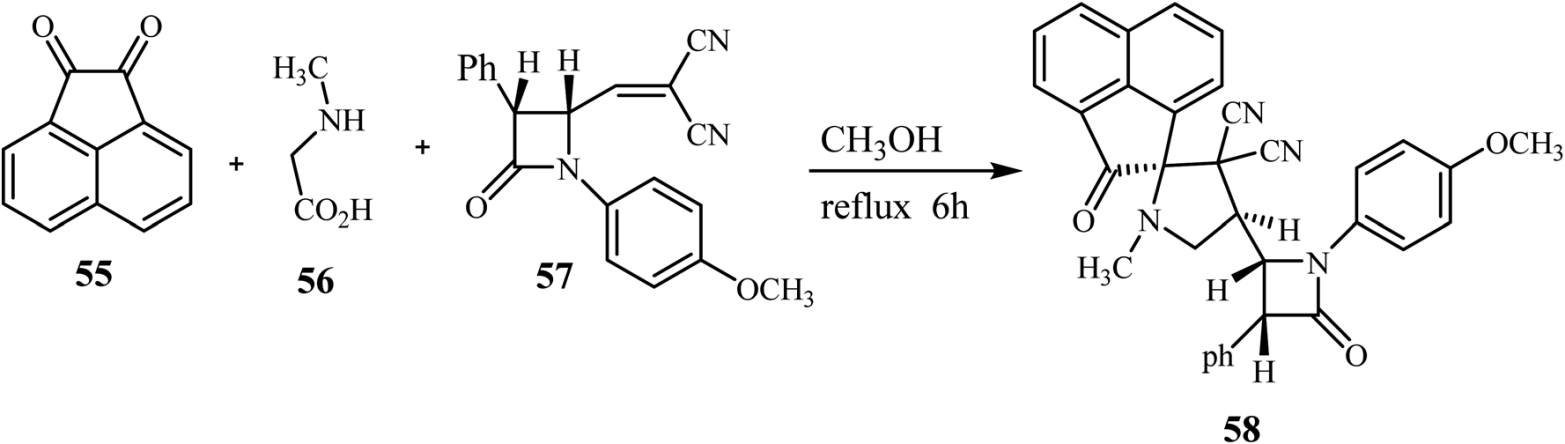
Synthesis of *β*-lactam grafted spiro acenaphthenopyrrolidines 58.

The azomethine ylide generated *in situ* by the condensation of acenaphthenequinone (55) with sarcosine (56) underwent smooth cycloaddition reaction with 57 regio- and stereo-selectively in refluxing CH_3_OH for 6 h affording exclusively the single diastereomer 58 in excellent yield (Scheme 15).^59^

**Scheme 16:**
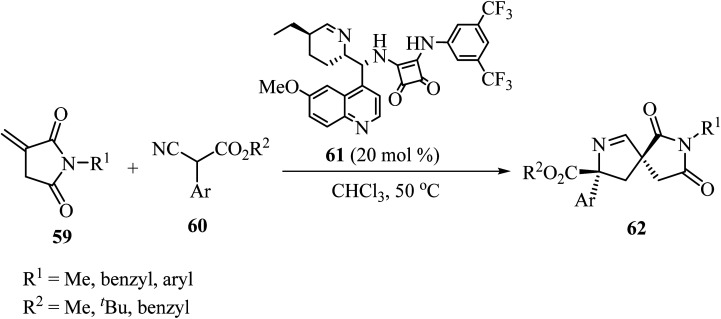
Formal [3 + 2] cycloaddition reaction of *N*-itaconimides 59 and isocyanoacetates 60 catalyzed by a chiral squaramide.

In 2019, Zhao and coworkers described the asymmetric synthesis of spiro pyrrolines 62 from isocyanoacetates 60 and nitaconimides 59 as Michael acceptors (Scheme 16).^60^ The process was catalyzed by squaramide 61 derived from dihydroquinine and provided the corresponding spirocyclic compounds 62 with good diastereo- and enantio-selectivity.^60^

**Scheme 17:**
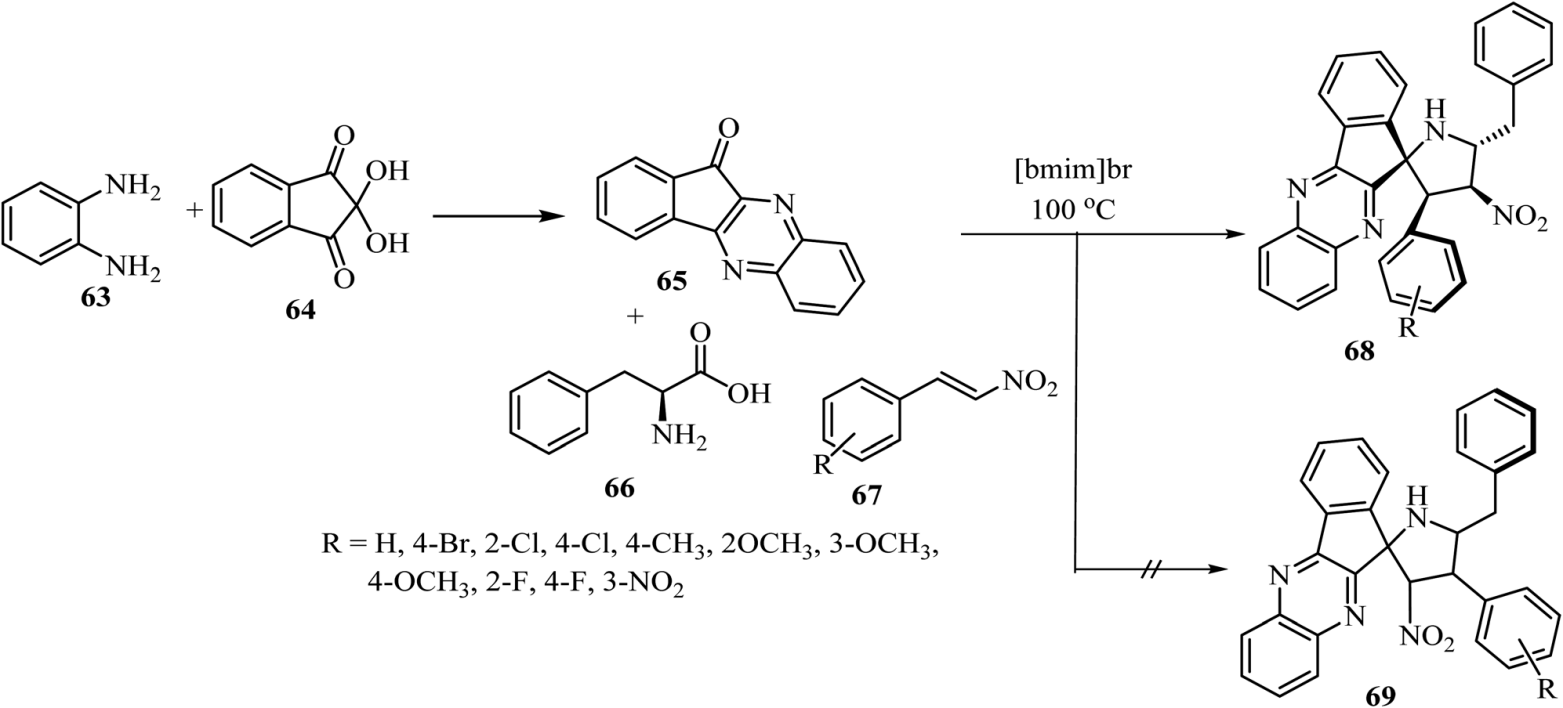
Synthesis of spiro-pyrrolidine tethered indenoqunoloxaline 68.

The spiro pyrrolidine tethered indenoquinoxaline heterocyclic hybrids 68 were synthesized by heating of *β*-nitrostyrene (67), *o*-phenylenediamine (63), ninhydrin (64), and l-phenylalanine (66) with stirring in [bmim]Br medium for 1 h at 100 °C (Scheme 17).^61^ Interestingly, the reaction was completely regioselective: the expected regioisomer 69 was not formed (Scheme 17).^61^*In vitro* activity of these spiroheterocyclic hybrids against *Mycobacterium tuberculosis* H37Rv, using MABA assay, revealed that the compound with nitro group on the phenyl ring is the most active candidate (1.56 μg mL^−1^) of the series and has an activity similar to that of the standard drug Ethambutol.^61^

**Scheme 18:**
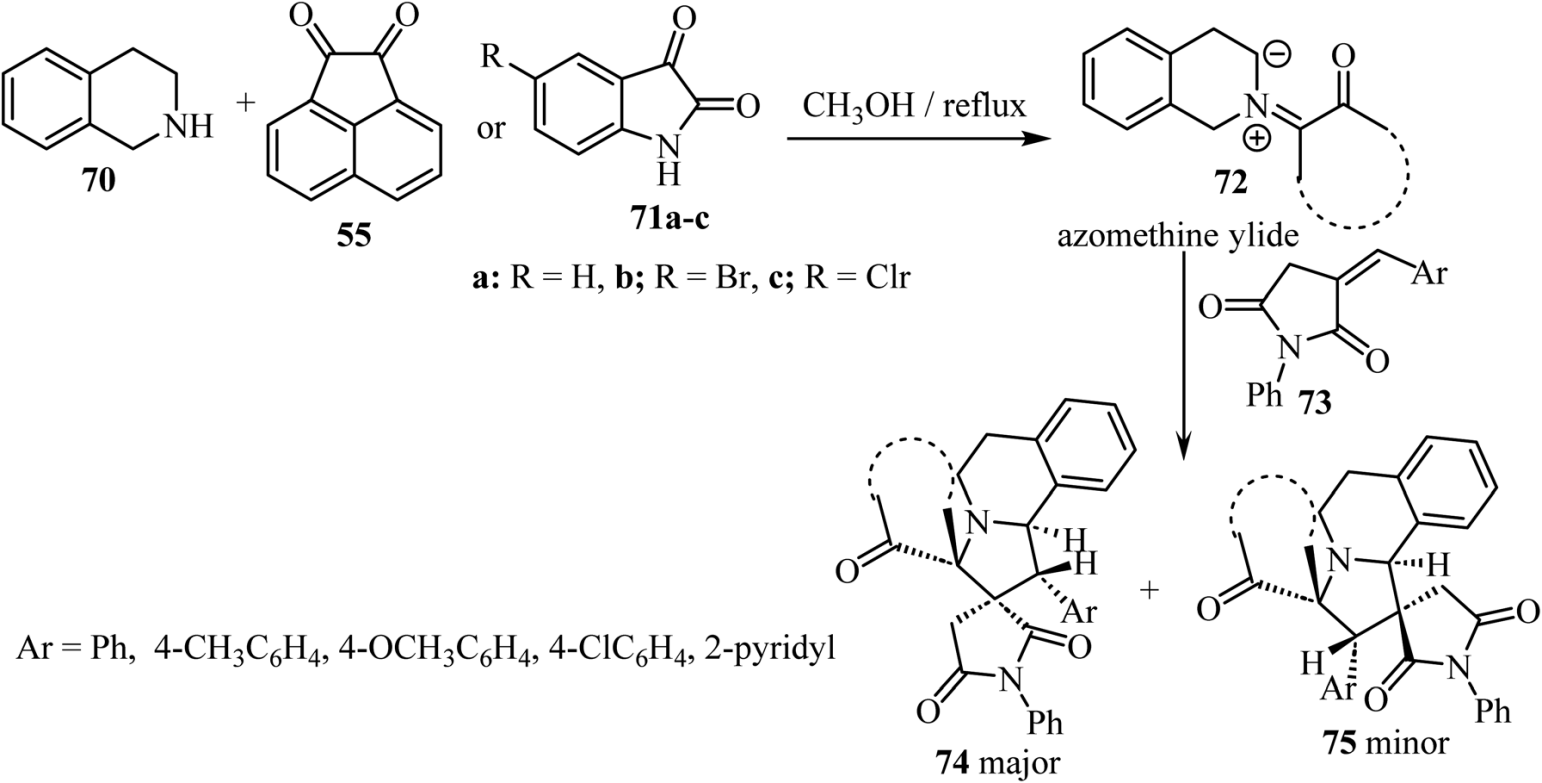
Synthesis of dispiropyrrolo[2,1-*a*]isoquinoline fused pyrrolidine-2,5-diones 74 and 75.

A diastereoselective approach^62^ to dispiropyrrolo[2,1-*a*]isoquinoline fused pyrrolidine-2,5-diones bearing two adjacent spirocarbons comprised a three-component 1,3-dipolar cycloaddition between cyclic diketones (isatin derivatives 71 or acenaphthenequinone, 55) with tetrahydroisoquinoline 70, to give *N*-ylides 72; these reacted with *α*-alkylidene succinimides 73 as dipolarophiles (Scheme 18). Among the various screened solvents (such as CH_3_OH, EtOH, CH_3_CN, and toluene), methanol (CH_3_OH) was selected as the most effective solvent and an unprecedented regioselectivity was observed in this cycloaddition reaction resulting in two products 74/75.^62^

**Scheme 19:**
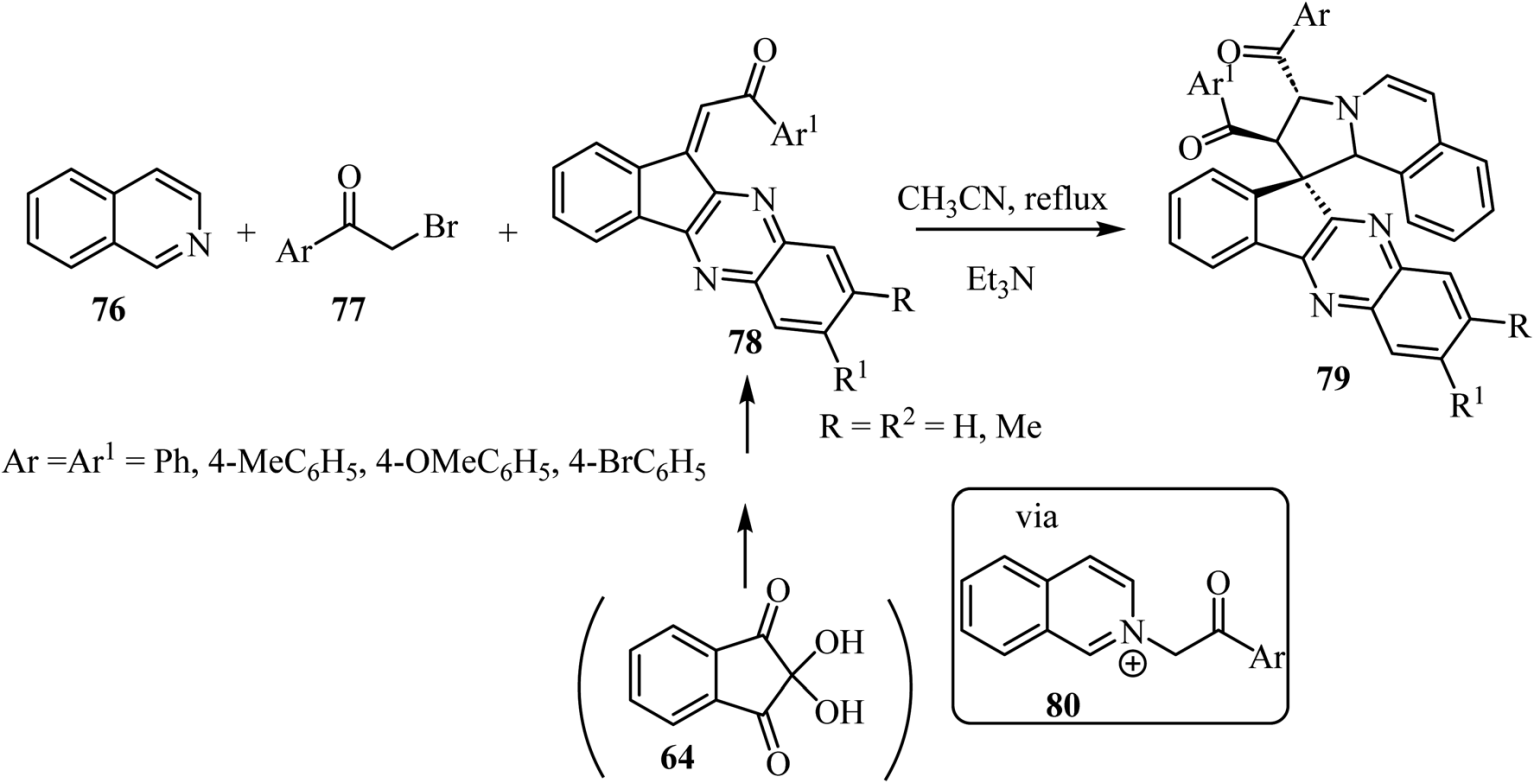
Spiropyrrolo[2,1-*a*]isoquinolines containing the indenoquinoxaline 79.

The construction of spiropyrrolo[2,1-*a*]isoquinolines containing the indenoquinoxaline frameworks 79 was achieved through the 1,3-dipolar cycloaddition reaction of isoquinolinium ylides, generated *in situ* by the reaction of isoquinoline (76) and phenacylbromides 77 in the presence of Et_3_N, with 1-aryl-2-(11*H*-indeno[1,2-*b*]quinoxalin-11-ylidene)ethanoates 78 (Scheme 19).^63^ The complex spiroheterocyclic products 79 contain four contiguous chiral centers. Importantly, the cycloaddition reaction affords only one regioisomer with high diastereoselectivity.^64–66^

**Scheme 20:**
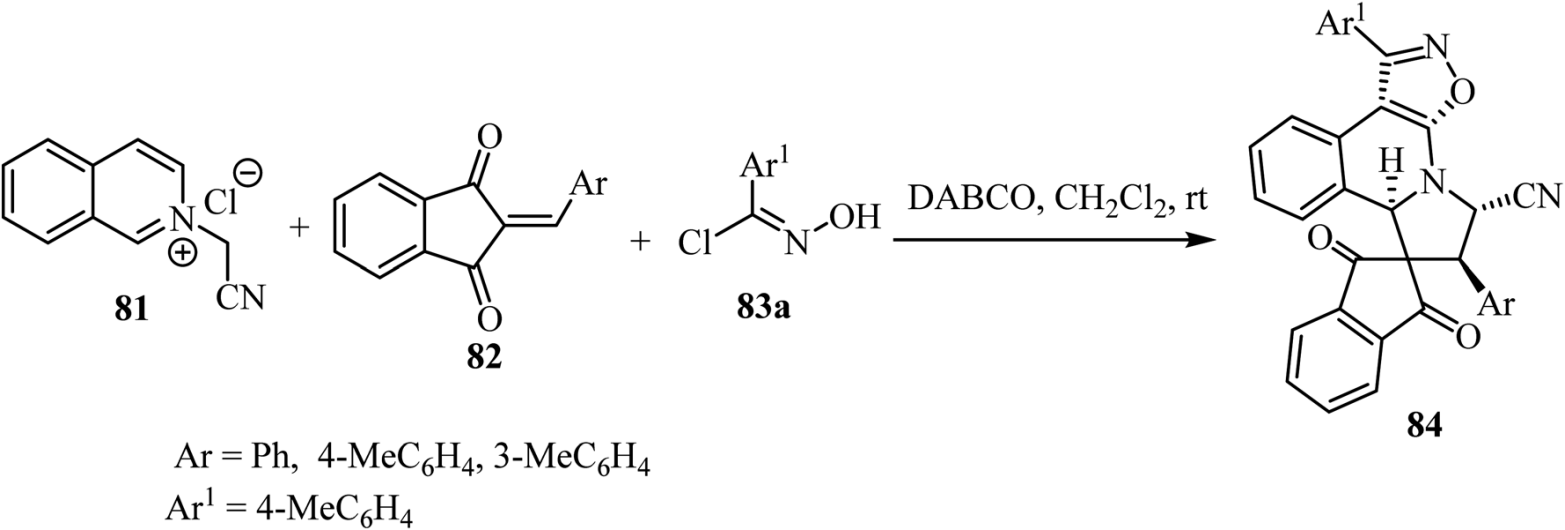
Synthesis of spiro[indene-2,8′-isoxazolo[5,4-*c*]pyrrolo[2,1-*a*]isoquinolines 84.

The one-pot cascade double [3 + 2] cycloaddition reaction of *N*-cyanomethyl isoquinolinium chloride 83 with 2-arylidene-1,3-indandiones 82 and (*E*)-*N*-hydroxybenzimidoyl chlorides 83, was carried out to access spiro[indene-2,8′-isoxazolo[5,4-*c*]pyrrolo[2,1-*a*]isoquinolines 84 in good yields with high diastereoselectivity (Scheme 20).^67^ The transformations were best performed in the presence of DABCO (1,4-diazabicyclo[2.2.2]octane) in CH_2_Cl_2_ as a solvent.^67^

### 2.3. Synthesis of spiroindol(one) derivatives

**Fig. 8:**
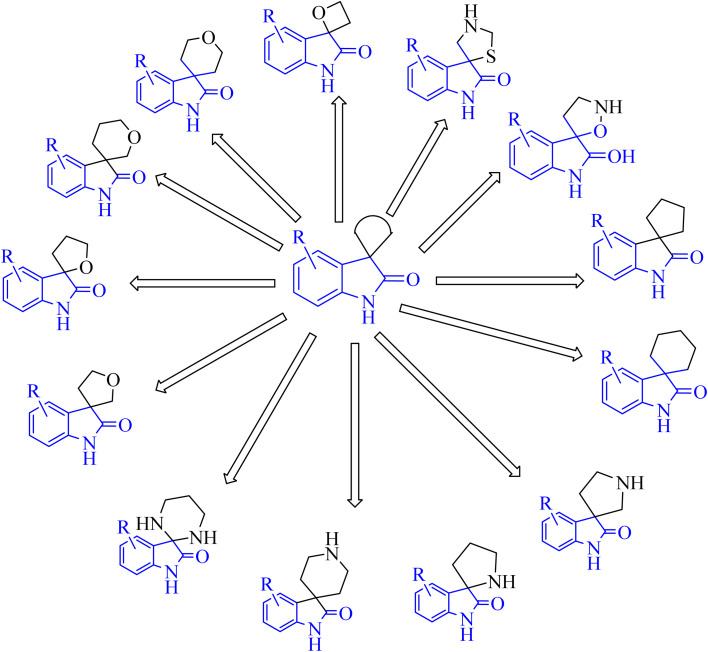
Diagram showed different spiro indol(one) derivatives.

Spiroindol(ones), which contain a spirocycle fused at the C2 or C3 of the oxindole moiety, are a known subset of indoles and form the core building blocks of highly functionalized organic structures. The procedures to obtain various spiro indolines and spiro indoles reported in the literature^68^ can be sorted into categories based on the type and size of the spirocycle that is fused to indole or oxindole such as 3-, 4-, 5-, or 6-membered rings, including different heteroatoms as illustrated in Fig. 8.^68^

**Scheme 21:**
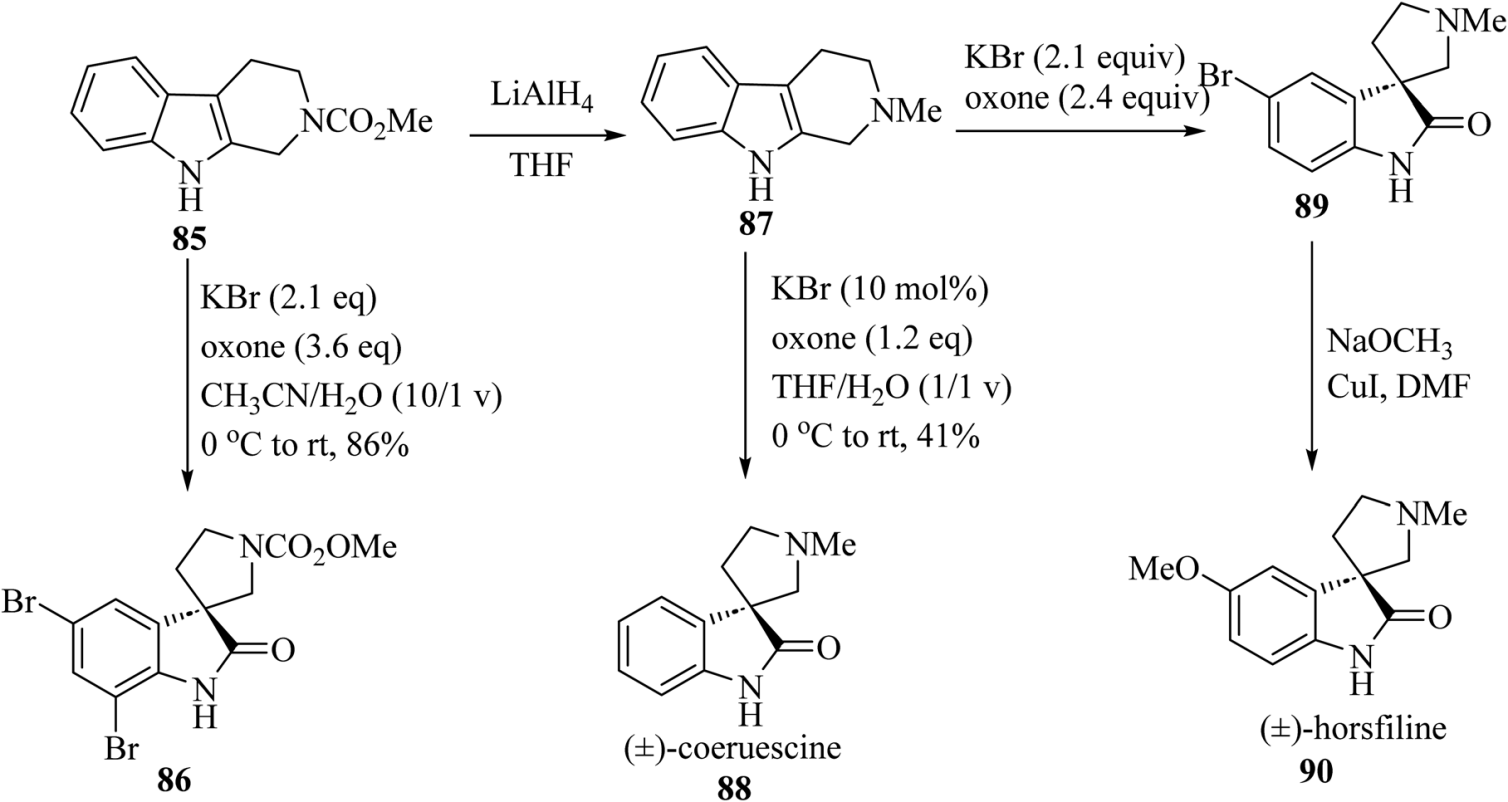
Oxidative rearrangement of tetrahydro-*β*-carbolines for the formation of spirooxindole natural compounds 86, 88 and 90.

The electron-rich property of indoles leads to easy oxidation using many reagents. Since catalysis methods in the presence of secure oxidants (H_2_O_2_, Oxone, O_2_) is highly favorable, Tong and co-workers^69^ have introduced three unique, efficient halide catalyzed oxidation processes of tetrahydro-*β*-carbolines (THCs) indoles applying oxone as the terminal oxidant, which leads to the formation of oxindoles 86, 88 and 90 (Scheme 21).^69^

**Scheme 22:**
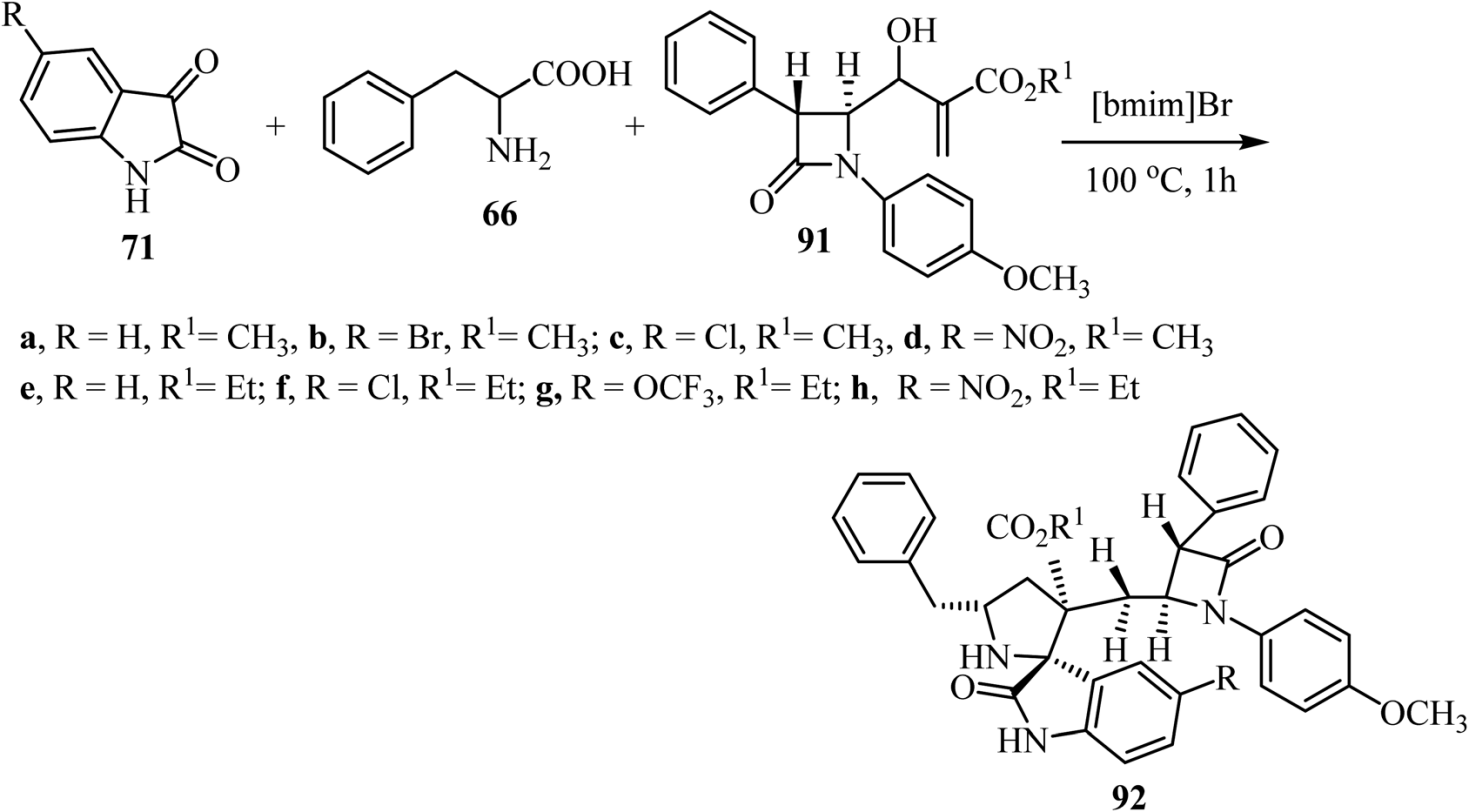
Synthesis of spiro oxindolopyrrolidine 92.

Spirooxindolopyrrolidine hybrid heterocycles 92, containing *β*-lactam subunits, were prepared as single diastereoisomers *via* 1,3-dipolar cycloaddition of Baylis–Hillman adducts 91 with azomethine ylides derived from isatins 71 and *α*-amino acid 66 under heating at 100 °C in [bmim]Br (Scheme 22).^70^ The *in vitro* antimycobacterium tubercular activity of hybrids 92 was assessed against *Mycobacterium tuberculosis* H37Rv. Members of the series with no substitution or chloro-substitution on the oxindole ring showed the most potent activity with a MIC 0.78 μg mL^−1^ and 1.56 μg mL^−1^, respectively, which were two-fold and equal activity than the standard drug, Ethambutol (MIC = 1.56 μg mL^−1^).^70^

**Scheme 23:**
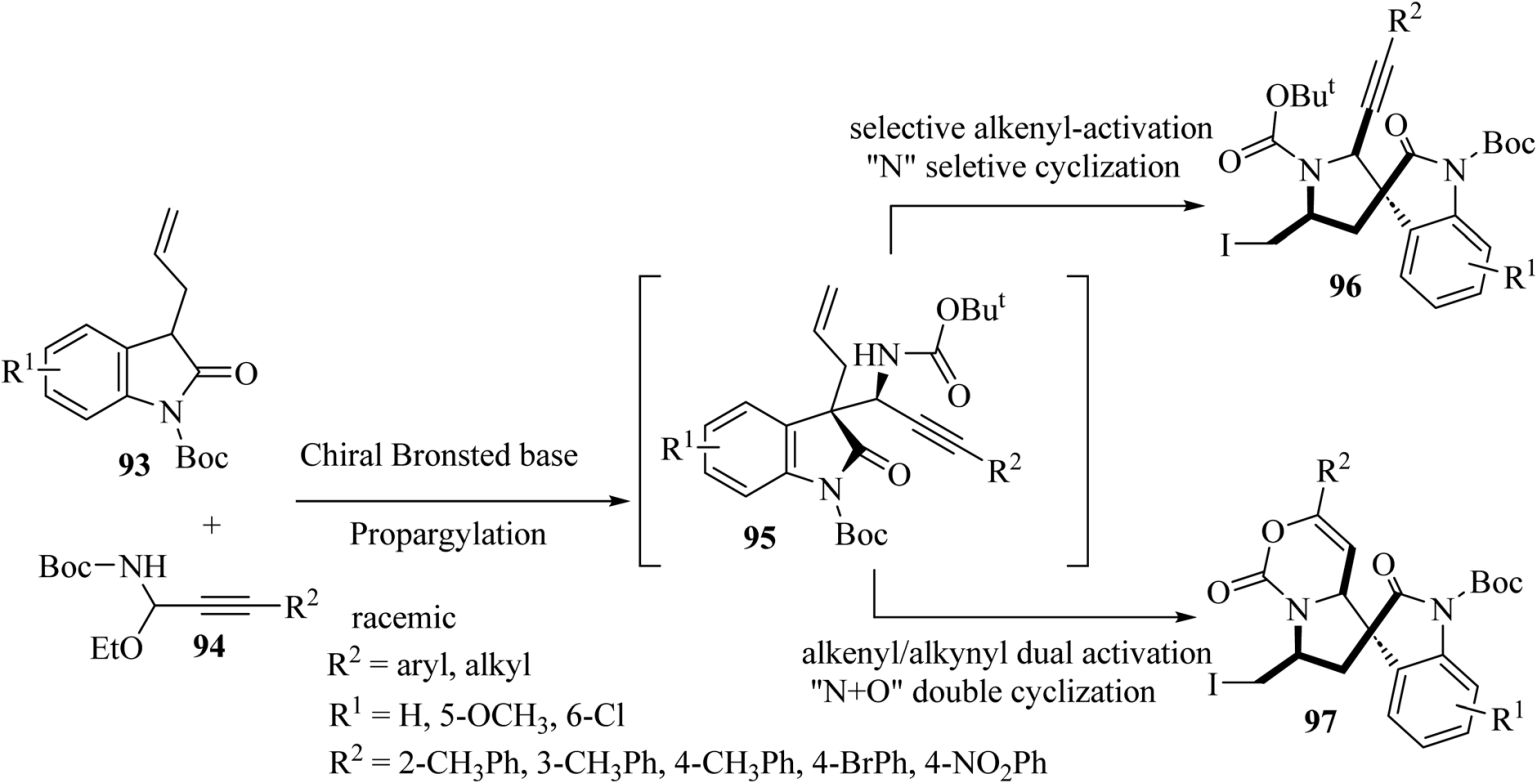
Asymmetric synthesis of tri-and tetracyclic spiro oxindoles 96 and 97.

Shao and colleagues introduced various catalytic asymmetric synthetic protocols for the formation of tricyclic and tetracyclic 3,3′-pyrrolidonyl spirooxindoles 96 and 97 (Scheme 23).^71^ This method proceeded through a one-pot asymmetric propargylation catalyzed by a chiral Brønsted base for the formation of oxindole 1,6-enynes 95 from the common and available precursors, 3-allyl oxindoles 93 and *C*-alkynyl *N*-boc acetal 94, and a subsequent interchangeable site-selective and excellent diastereoselective electrophilic iodocyclization of 1,6-enynes *via* alkenyl-activation and alkenyl/alkynyl dual activation to form tricyclic 3,3′-pyrrolidonyl spirooxindoles 96 and tetracyclic 3,3′-pyrrolidonyl spirooxindoles 97 (Scheme 23).^71^ The obtained tricyclic and tetracyclic spirooxindoles were preliminarily evaluated for *in vitro* anticancer activities. They displayed potential anticancer activities and some compounds exhibited the best inhibitory activity against HeLa and SGC7901 cell lines with IC_50_'s of 13.2 and 17.6 μM, respectively.^71^

**Scheme 24:**
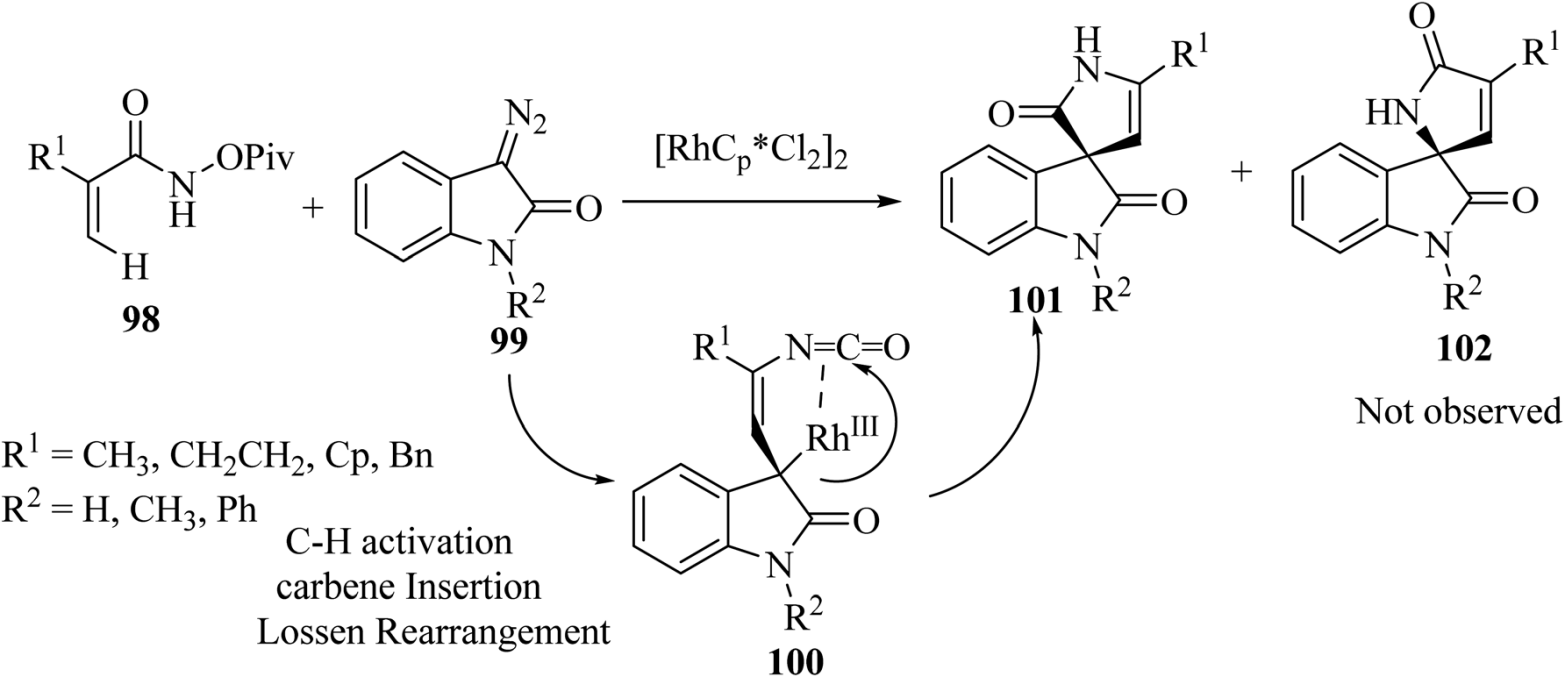
C(sp^2^)–H activation/annulation on to synthesis spiro oxindole pyrrolone scaffolds 101.

Rh(iii)-catalyzed domino annulations of *N*-(pivaloyl-oxy)acrylamides 98 with diazooxindole 99 gave spiro-oxindole pyrrolone products 101 with excellent regioselectivities; the alternative regioisomer 102 was not observed (Scheme 24). The potential application of this protocol in the next step of diversification for drug finding was illustrated in the directed presentation of spiro-oxindole-pyrrolone skeleton into medicinal molecules pentoxifylline, endo folliculin, and pregnenolone.^72^

**Scheme 25:**
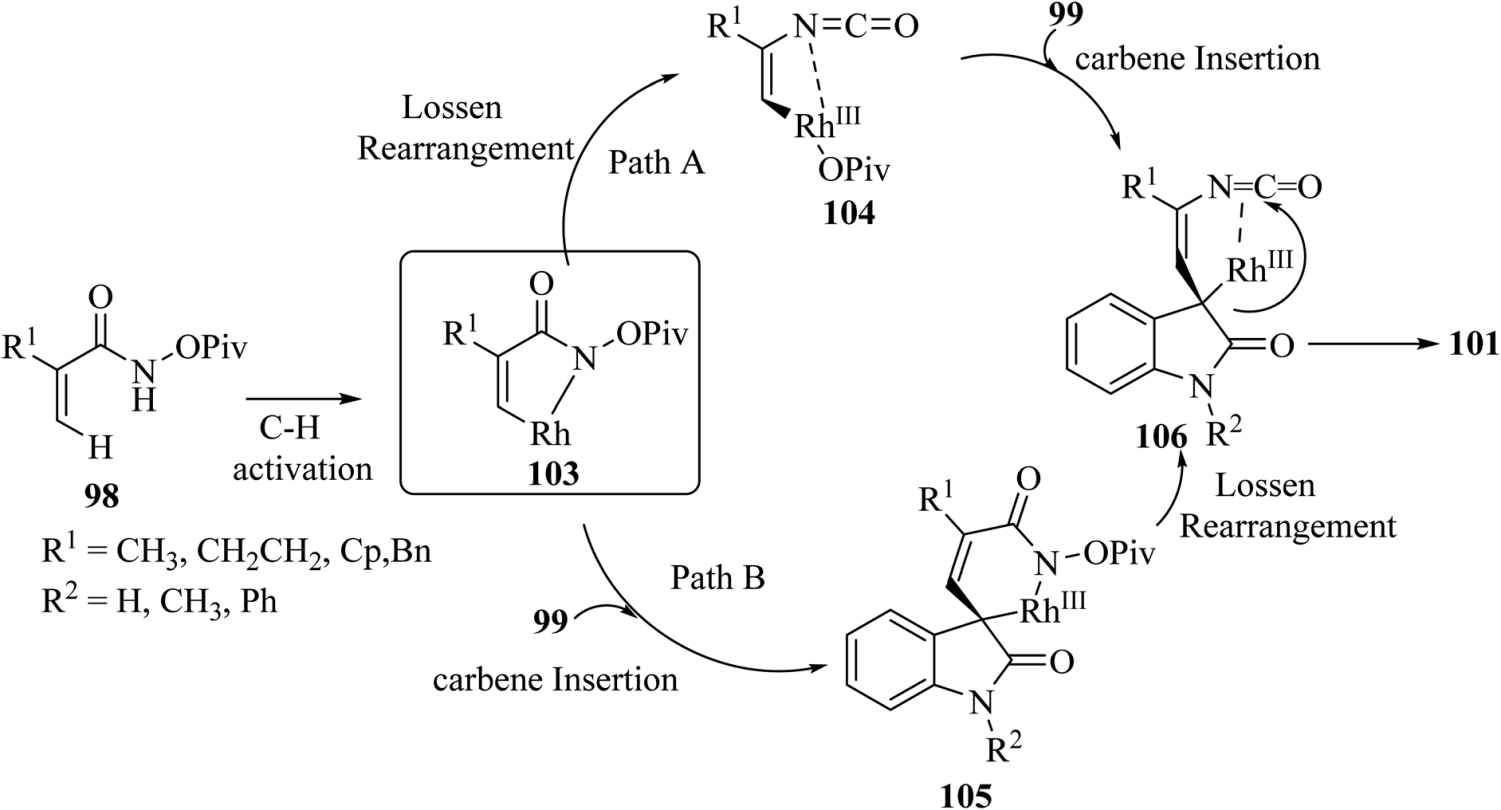
Rational mechanism for the synthesis of spiro oxindolpyrrolone 101.

It was tentatively proposed that the reaction involves cleavage of the C–H bond with Rh(iii), followed by carbene migratory insertion with the diazo-substrate to obtain an alkyl rhodium intermediate 105. Then, a formal Lossen rearrangement offers isocyanate 106, which, *via* further nucleophilic addition at the isocyanate, intramolecularly generates the final compound 101 (Scheme 25).^72^

**Scheme 26:**
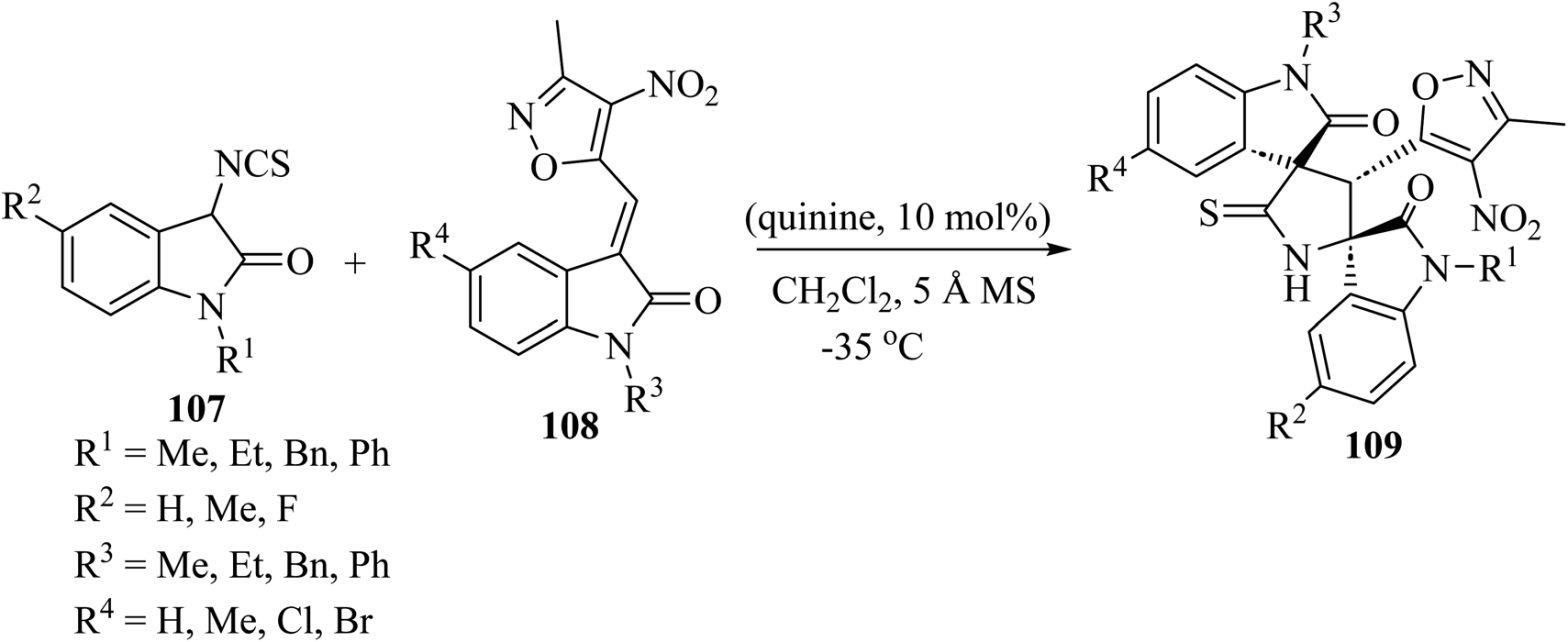
Synthesis of isoxazole-dispiro-bis-oxindoles 109 from 3-isothiocyanato oxindoles 107 and isatylidenyl isoxazoles 108 by an organocatalytic process promoted by quinine.

Providing an electron-withdrawing moiety for alkylidenyloxindoles acting as dipolarophiles, the asymmetric Michael addition/cyclization cascade reaction of 3-isothiocyanato oxindoles 107 and 3-methyl-4-nitro-5-isatylidenyl isoxazoles 108, catalyzed by quinine, yielded enantiomerically enriched isoxazole-dispirobisoxindoles 109 (Scheme 26).^73^ Although enantioselectivities were found to be dependent on the protecting group of the nitrogen atom of the isothiocyanato oxindole 107 and the isatylidenyl isoxazole 108, and on the electronic character of the substitution of both aromatic rings, excellent diastereo-selectivity was achieved in almost all cases and high reactivity was observed, with reaction times of only 30 min.^73^

**Scheme 27:**
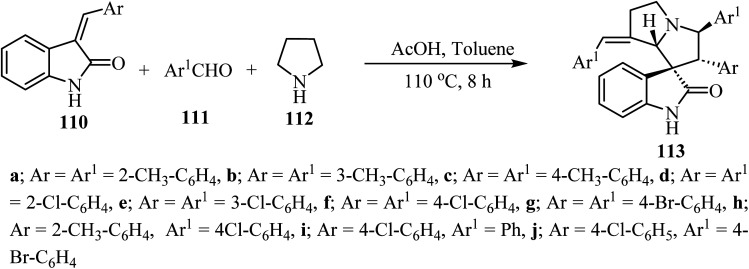
Synthesis of spiroindoles 113.

The synthesis of complex spiro heterocyclic compounds 113 has been pursued, by cycloaddition of *in situ* generated azomethine ylides with 3-alkylidene-2-oxindoles 110 as dipolarophiles. This acetic acid-promoted three component reaction 110, aldehydes 111 and pyrrolidine (112) gives the resulting cycloadducts with good yields and diastereoselectivity (50–80% yields, single diastereomer). The reaction mechanism involved *β*-C–H functionalization of pyrrolidine, generation of the azomethine ylide intermediate and subsequent 1,3-dipolar cycloaddition (Scheme 27).^74^ The resulting spiro oxindole derivatives were investigated by evaluation against mouse colon cancer cells CT26 and human liver cancer cells HepG2 by MTT assay.^74^

**Scheme 28:**
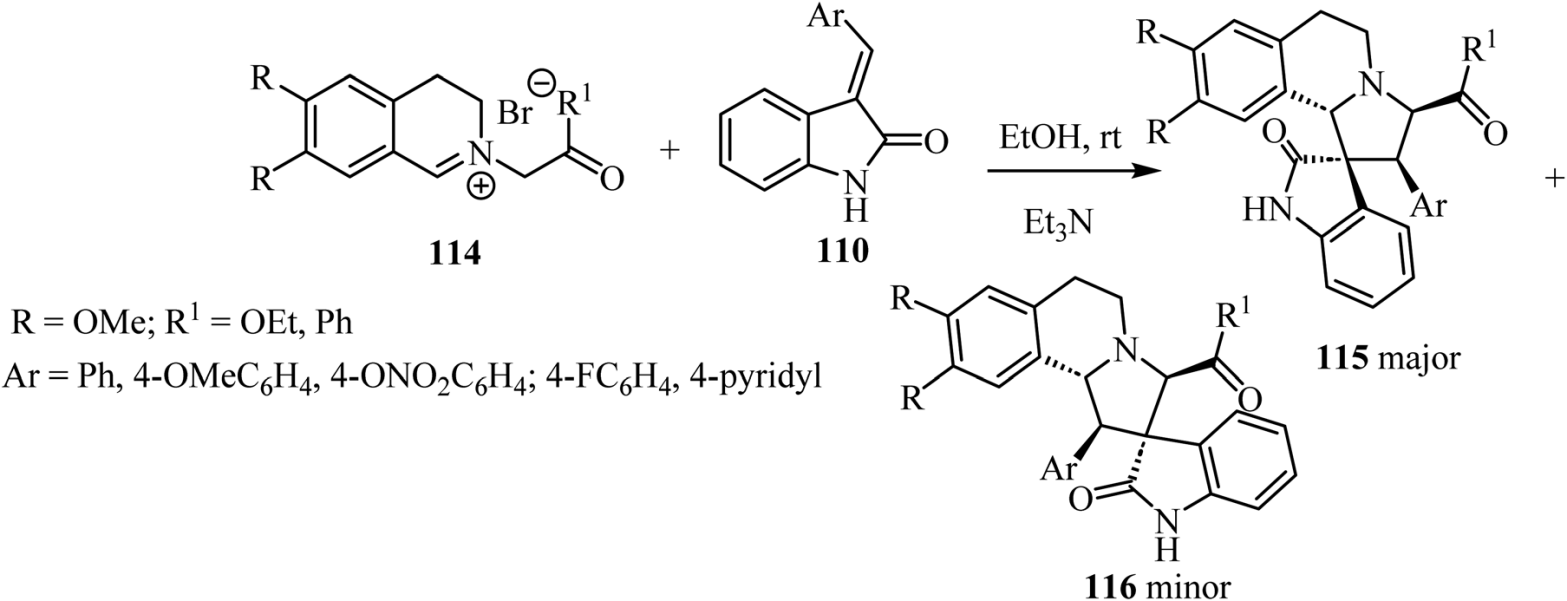
1,3-Dipolar cycloaddition reaction of isoquinolinium salts 114 with 3-arylidene-indoline-2-ones 110.

1,3-Dipolar cycloaddition reaction of isoquinolinium salts 114 with 3-arylideneindoline-2-ones 110 (Scheme 28) in the presence of trimethylamine (Et_3_N) accomplished the regioselective formation of spiro pyrrolidine oxindoles 115/116 (Scheme 28). The main products 115 were formed in most cases as a white precipitate in the reaction mixture, and could be separated by simple filtration to give the single diastereoisomer 115 (in up to 75% yield). The minor isomer 116 could be isolated from the mother liquor using preparative HPLC.^75^

**Scheme 29:**
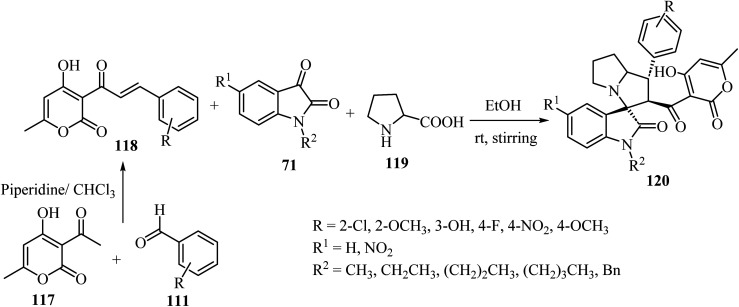
Formation of hexahydrospiro[indoline-3,3′-pyrrolizine]-2-ones 120.

Tripathi *et al.*^76^ have described the regioselective synthesis of hexahydrospiro[indoline-3,3′-pyrrolizine]-2-ones 120 in good-to-excellent efficiencies through [3 + 2] cycloaddition. The products were obtained *via* reaction of substituted 3-pyran-2-ones 118, isatin derivatives 71, and l-proline (119) at ambient temperature (Scheme 29). The chalcones 118 used in this reaction were produced *via* aldol condensation of substituted benzaldehydes 111 and 3-acetyl-4-hydroxy-6-methyl-2*H*-pyran-2-one (117) in dry chloroform (CHCl_3_) using a catalytic amount of piperidine (Scheme 29).^76^

**Scheme 30:**
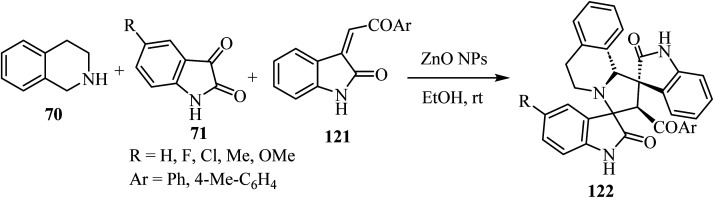
Three component azomethine ylide cycloadditions catalyzed by unsupported partially aggregated ZnO nanoparticles (NPs).

Scheme 30 shows that the dispiro-2′,4′-(2-oxindolo)indolizidine 122 skeleton^77^ was obtained at room temperature in EtOH or water (83–97% yield). Condensation between tetrahydroisoquinoline 70 and isatin derivatives 71 provided an azomethine ylide intermediate, whose subsequent cycloaddition with the appropriate dipolarophile gave the target molecules 122 as single diastereoisomers. Curiously, these reactions occurred in the absence of a basic agent. Inexpensive ZnO nanoparticles (NPs) were tested in order to determine their recyclability. Unfortunately, the efficiency of the nano-catalyst declined at every cycle, and after the third one the nanoparticles resulted as aggregates^77^ (Scheme 30).

**Scheme 31:**
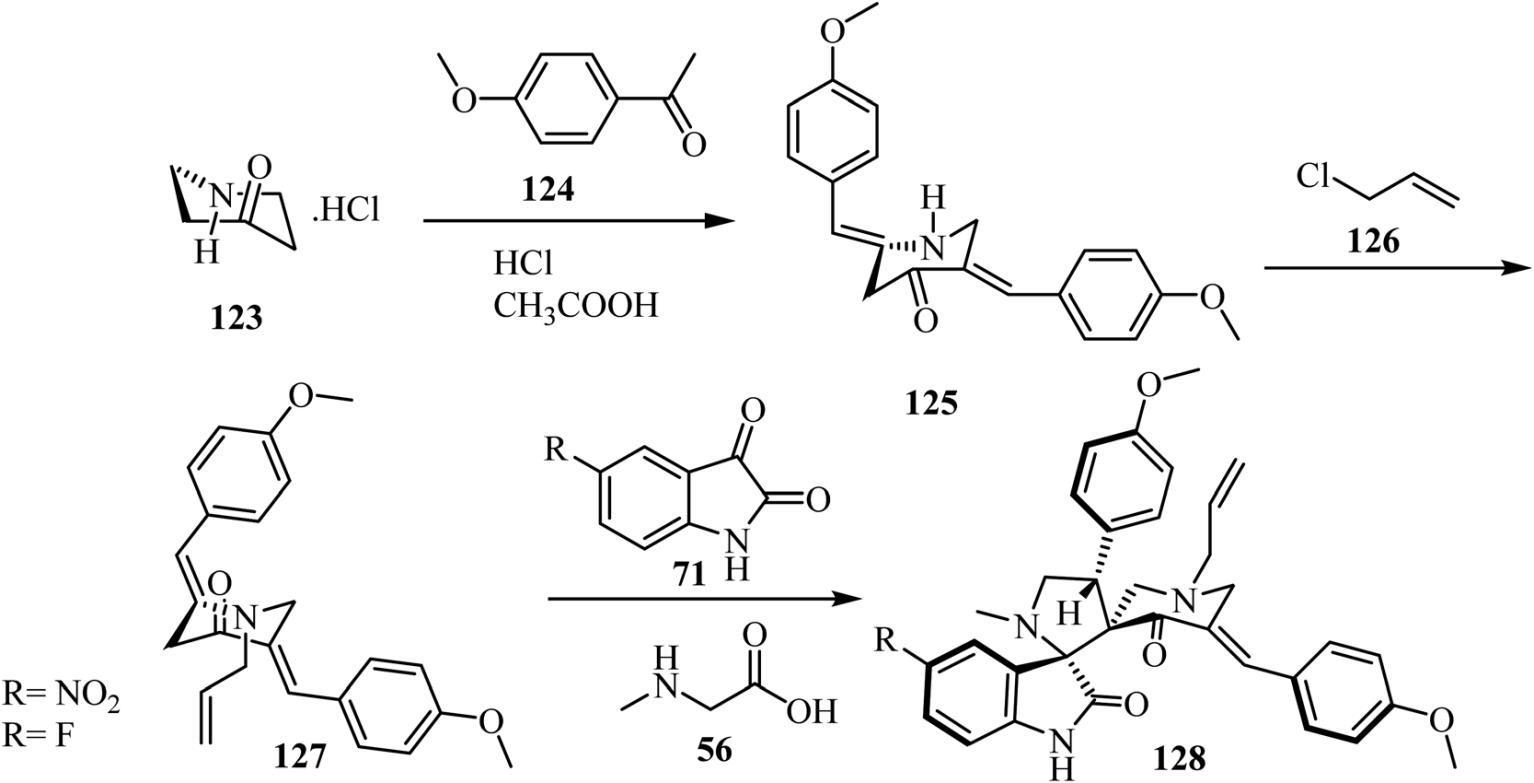
Synthesis of spirooxindole-pyrrolidine hybrids 128.

Synthesis of *N*-allyl-bis(methoxyphenylmethylidene)piperidone (127), starting with piperidin-4-one·HCl (123), is outlined in Scheme 31. The spirooxindole-pyrrolidine 128 was extracted in good yield (86%) *via* the cycloaddition of dipolarophile 127 ^78^ with the azomethine ylide generated *in situ* from isatin derivatives 71 and sarcosine (56) at reflux in CH_3_OH for 1 h. The same reaction was performed in [bmim]Br at 100 °C (Scheme 31). TLC analysis of the reaction mixture revealed completion of the reaction in about 30 min with formation of the sole reaction product. The reaction mixture was then extracted with ethyl acetate and further purified by column chromatography. The reaction in [bmim]Br afforded a slightly better yield (90%) of 128 over the conventional heating employing CH_3_OH (86%) (Scheme 31).^78^

**Scheme 32:**
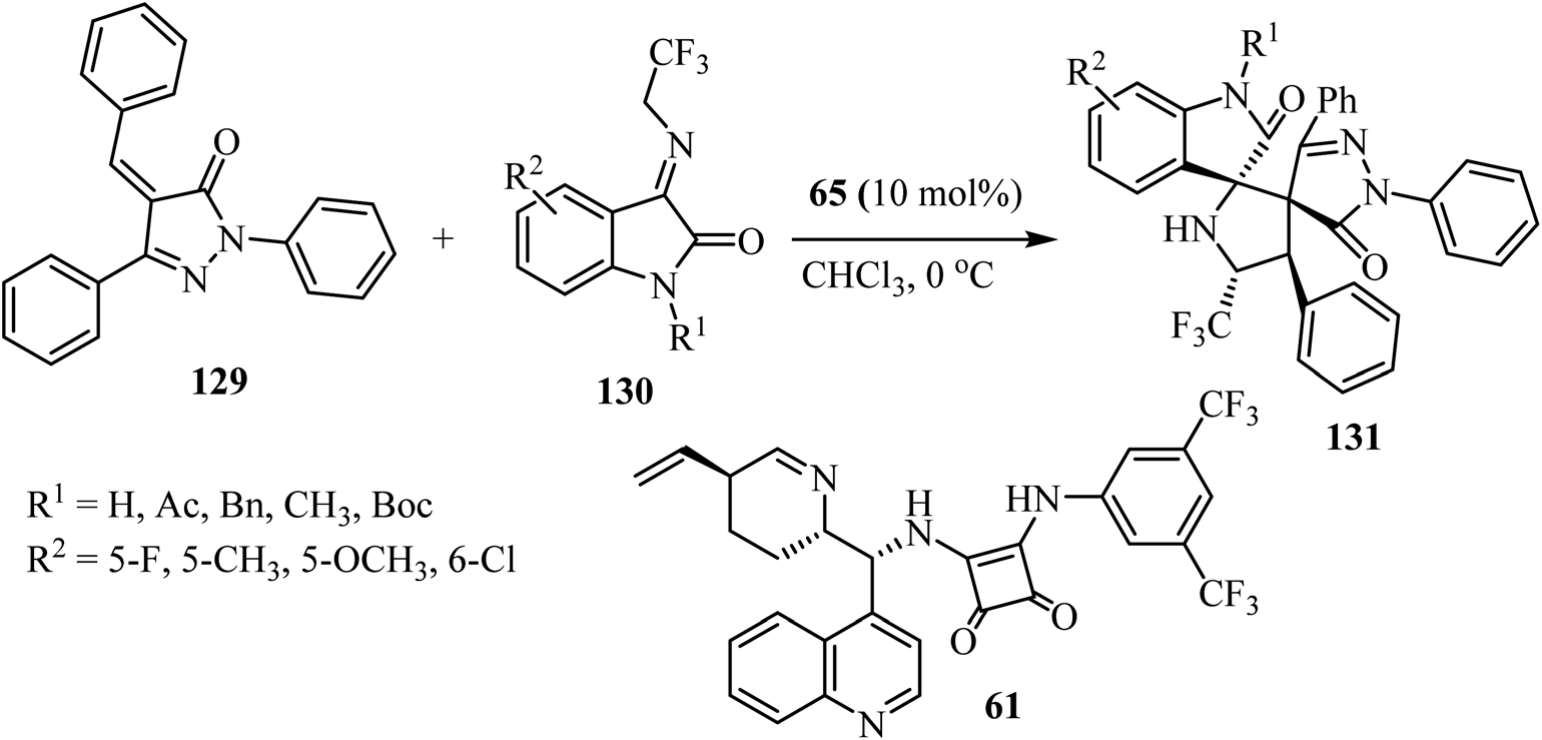
Synthesis of spiro-indolones 131.

Yan's group^79^ have described a procedure for the formation of CF_3_-containing spiro-oxindole-pyrrolidinepyrazolone compounds 131*via* organo-catalytic [3 + 2] cycloaddition. This reaction involves the cycloaddition of *α*,*β*-unsaturated pyrazolones 129 with *N*-2,2,2-trifluoroethylisatin ketimines 130 in CHCl_3_ at ambient temperature in the presence of a cinchonine-derived squaramide 65 catalyst to produce a pyrrolidine spiro-fused with both oxindole and pyrazolone (131) with four adjacent stereocenters and two adjacent spiro-quaternary chiral centers, in high efficiencies and stereoselectivities (Scheme 32).^79^

**Scheme 33:**
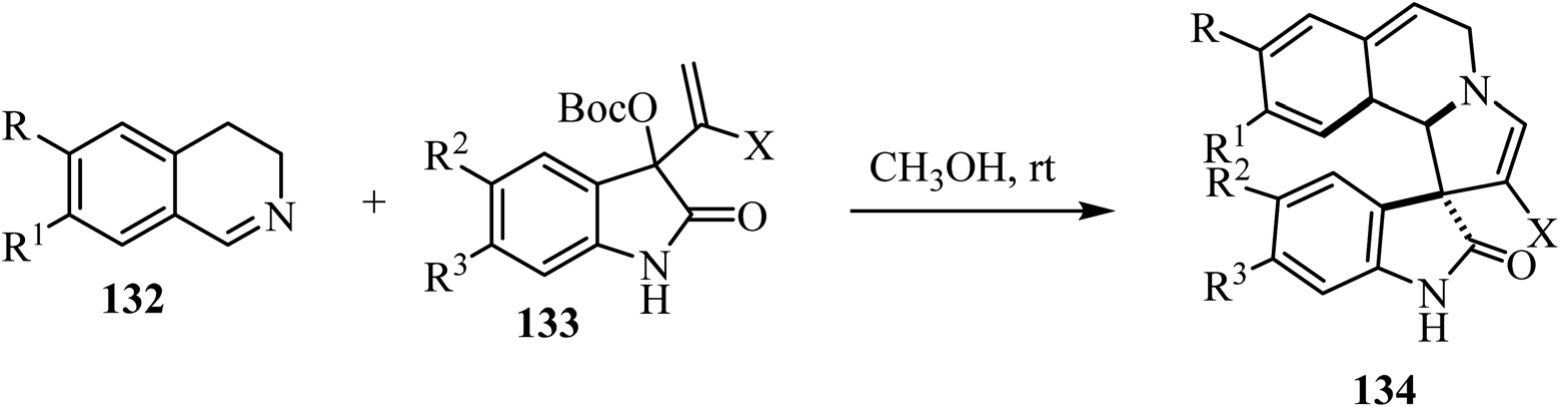
Synthesis of spiroindoles 134.

A mild catalyst-free [3 + 2] cyclization of dihydro-isoquinolines 132 and the isatin-based Morita–Baylis–Hillman (MBH) carbonates 133 has been investigated (Scheme 33).^80^ The combination of dihydroisoquinolines and the spirooxindole skeletons was achieved, leading to richly decorated spiro heterocycles 134 in moderate to good yields with good stereocontrol.^80^

**Scheme 34:**
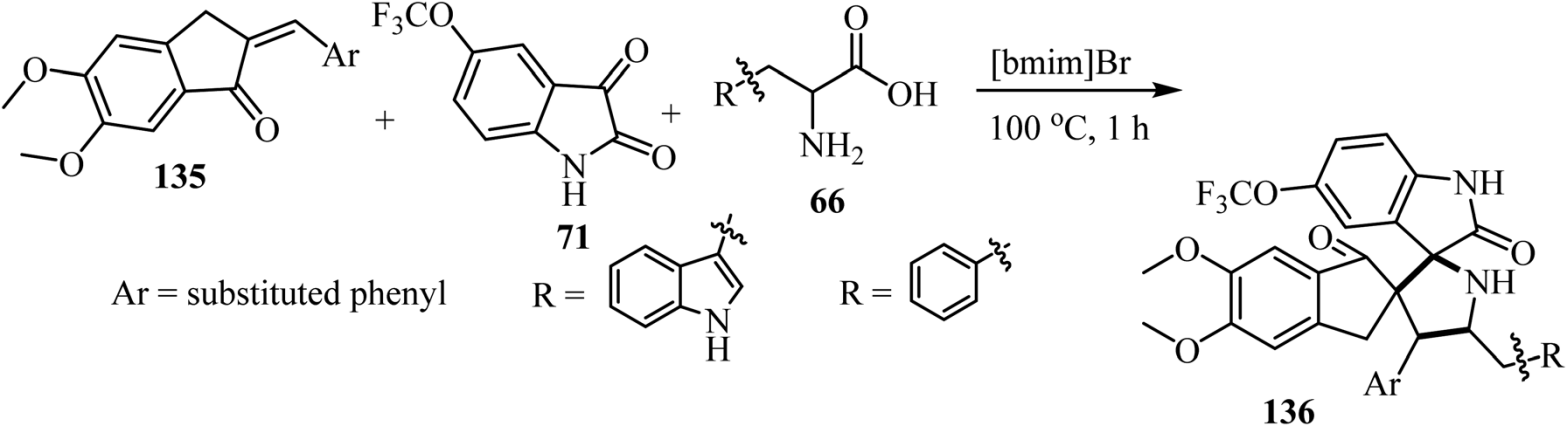
Synthesis of dispiro[indene-2,3′-pyrrolidine-2′,3′′-indoline]-1,2′′(3*H*)-diones 136.

The one-pot three-component cycloaddition reaction of 2-arylmethylidene-5,6-dimethoxyindenones 135 with azomethine ylides generated *in situ* from 5-(trifluoromethoxy)isatin (71) and tryptophan/phenylalanine 66 in [bmim]Br furnished the spiropyrrolidine heterocyclic hybrids 136 in moderate to good yields. Among the spiro pyrrolidine heterocyclic hybrids, the indole based fluorinated compound with a methoxy substituent at the *meta*-position of the aryl ring exhibited the utmost potent AChE and BChE inhibition with IC_50_ values of 1.97 ± 0.19 μM and 7.08 ± 0.20 μM, respectively (Scheme 34).^81^

**Scheme 35:**
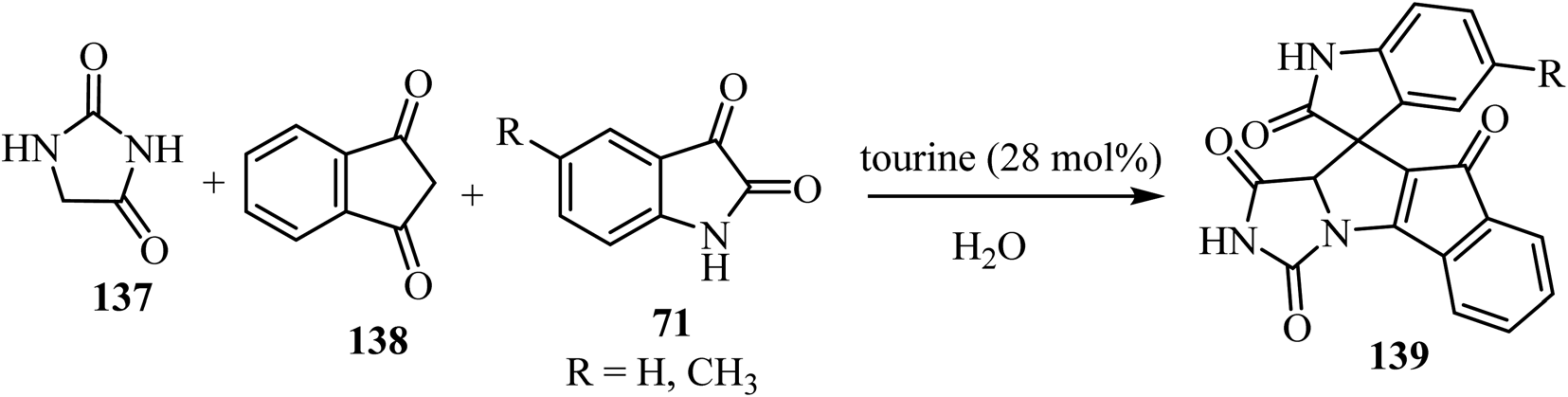
Synthesis of spiro[indeno[2′,1′:4,5]pyrrolo[1,2-*c*]imidazole-10,3′-indoline]-tetraone derivatives 139.

The three-component domino reaction of isatin derivatives 71, 1,3-diketone (138), and hydantoin (137) without catalyst did not succeed either in the absence of solvent, or in water or EtOH as solvents: the desired product 139 was not obtained after stirring for 12 h (no result).^82^ However, using either piperidine, 1,4-diazabicyclo[2.2.2]octane (DABCO), 1,8-diazabicyclo[5.4.0]undec-7-ene (DBU), or taurine (2-aminoethanesulfonic acid) as a catalyst in H_2_O as solvent, the desired products 139 were obtained in appreciably good yield (Scheme 35). But when the reaction was carried out in the presence of l-proline as a catalyst using H_2_O as a solvent, comparatively better yield of the desired product was obtained.^82^ An excellent yield of 139 was obtained when taurine (28 mol%) was used as a catalyst using H_2_O as a solvent (Scheme 35).^82^

**Scheme 36:**
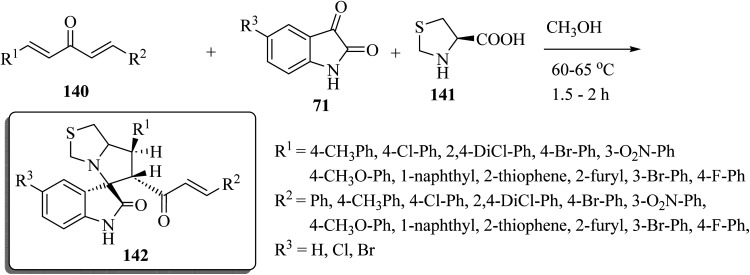
Synthesis of spiro[indoline-3,5′-pyrrolo[1,2-*c*]thiazol]-2-ones 142 with four stereogenic centers.

Spiro[indoline-3,5′-pyrrolo[1,2-*c*]thiazol]-2-one (142) was synthesized as shown in Scheme 36. One-pot multi-component condensation of *α*,*β*-unsaturated dienones 140 with the isatin derivatives 71 and amino acid derivatives 141 (l-4-thiazolidinecarboxylic acid) in CH_3_OH at reflux produced the spiro-oxindole series 142 (Scheme 36).^83^ The anticancer activities of compounds 142 were tested against colon (HCT-116), prostate (PC-3), and hepatocellular (HepG-2) cancer cell lines; some compounds inhibited colony formation, cell migration, arrested cancer cell growth at G2/M, and induced apoptosis through intrinsic and extrinsic pathways.^83^

**Scheme 37:**
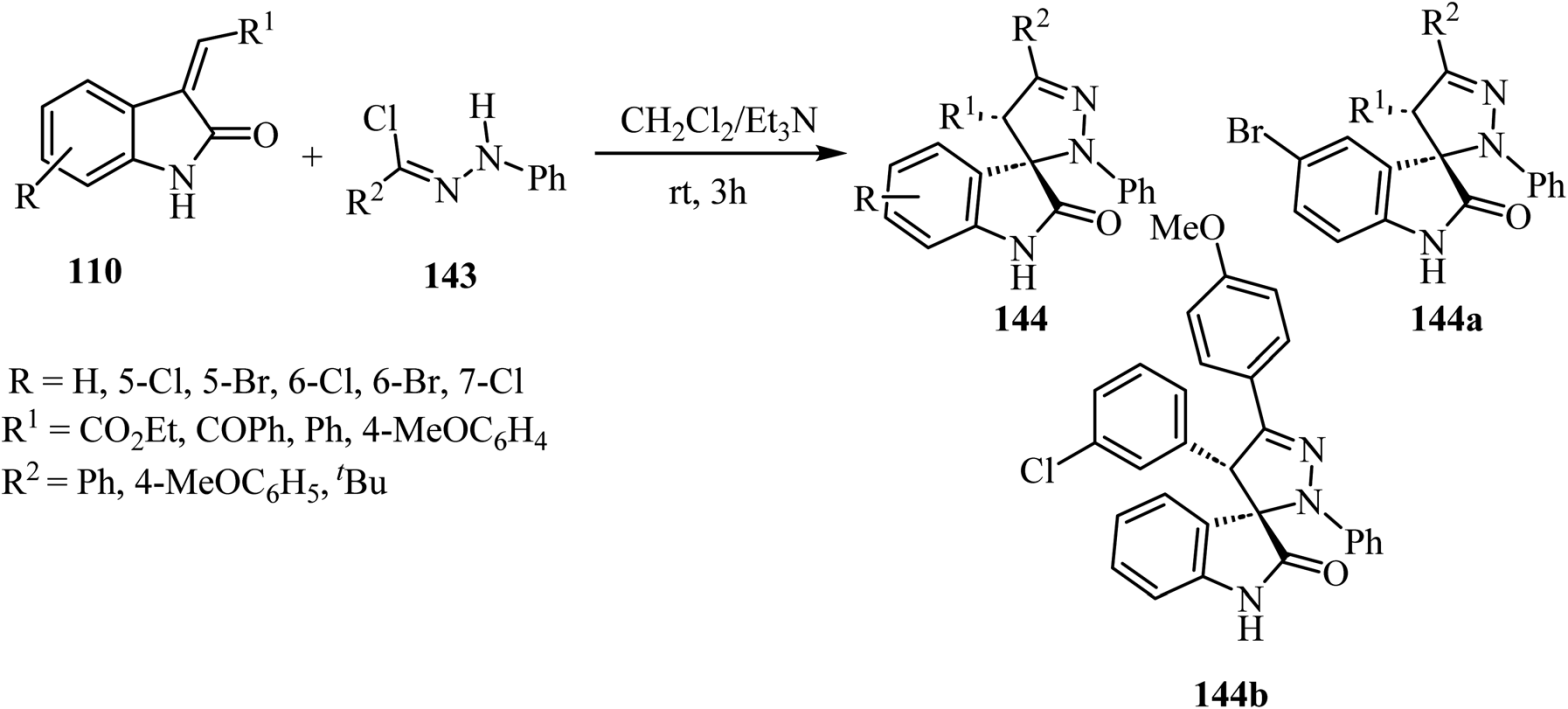
Nitrilimine cycloaddition to 3-alkylidene-2-oxindoles 110 hydrazonoyl chloride 143.

The synthesis of racemic spiro(2-oxindolo)pyrazolines 144 was accomplished by treating hydrazonoyl chlorides 143 with 3-alkenyl-2-oxindoles 110 in the presence of triethylamine (Et_3_N) as the organic basic agent and CH_2_Cl_2_ as a solvent. The regioselective cycloaddition of the dipolar intermediate gave 19 examples of the spiro cycloadducts with 80–90% yield (Scheme 37).^84^ Other examples were provided similarly.^85,86^ Biological evaluation of the so-obtained spiro(2-oxindolo)pyrazoline library showed antiproliferative activity in HCT-116p53(+/+) human colorectal cancer cell line with two derivatives displaying good activities (144a: IC_50_ = 13.1 ± 1.0 μM, 144b: IC_50_ = 10.9 ± 0.8 μM), see Scheme 37. Both spiro(indolo)pyrazolines 144a,b were able to induce apoptosis and cell cycle arrest. Cytotoxic effects induced by 144a occurred in cancer cells without eliciting cells death in non-malignant human colon fibroblasts. Furthermore, it was demonstrated that the combination of 144a with subtoxic concentrations of the chemotherapeutic agent 5-fluorouracil exerted a synergistic inhibitory effect on HCT-116 colon cancer cell proliferation.^84^

**Scheme 38:**
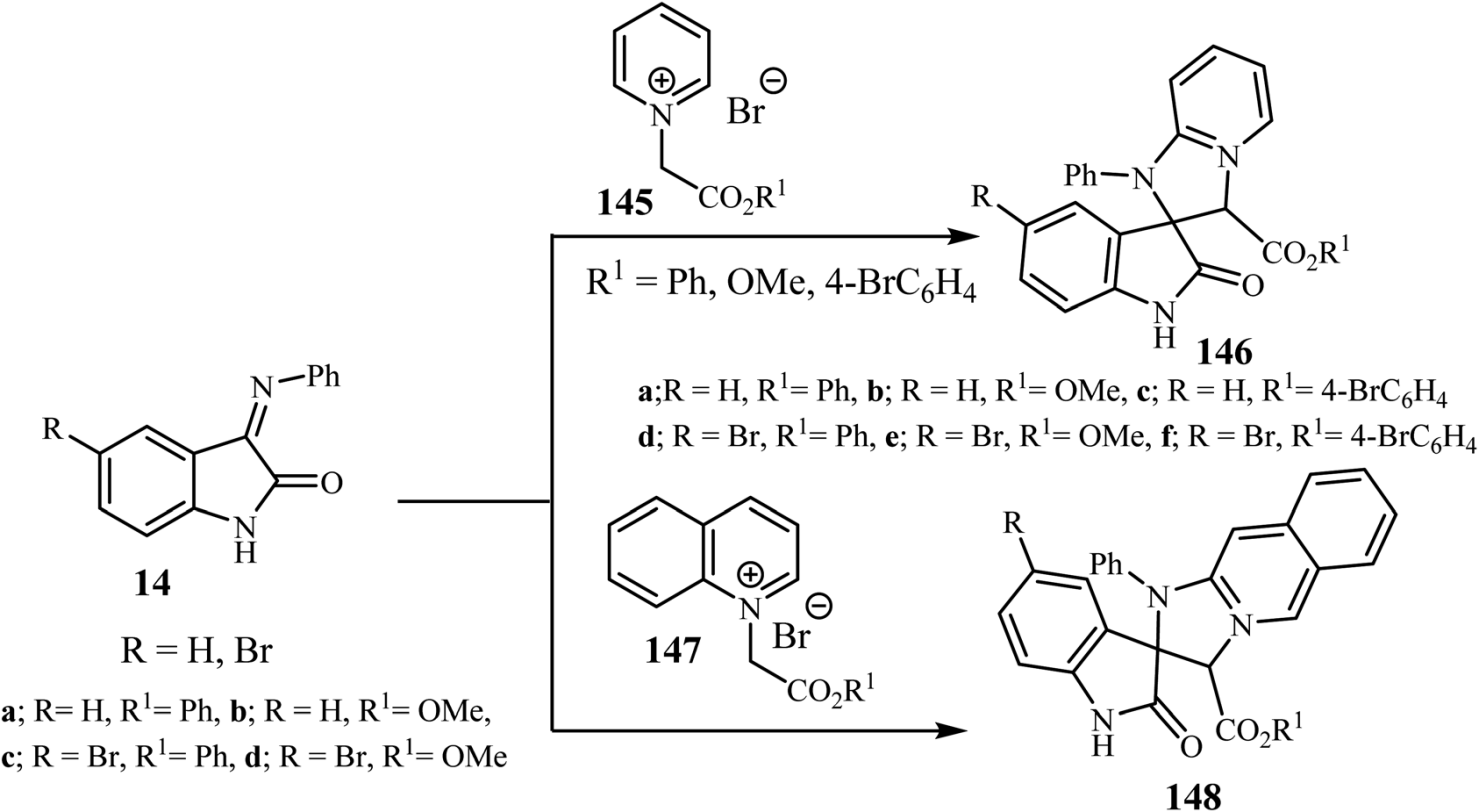
Cycloaddition between isatin-3-imines 14 and pyridinium or isoquinolinium ylides 145 and 147.

Sheibani and co-workers^87^ reported that cycloaddition between isatin-3-imines 14 and pyridinium or isoquinolinium salts gave racemic spiro(2-oxindolo) imidazolines 146/148 in 90–95% yield. Mild conditions, operational simplicity and easily accessible starting materials were features of these cycloadditions (Scheme 38).^87^ The cycloaddition reactions involved the nucleophilic attack by the isoquinolinium-ylides on isatin-3-imines 14 which act as a dipolarophile.^87^

**Scheme 39:**
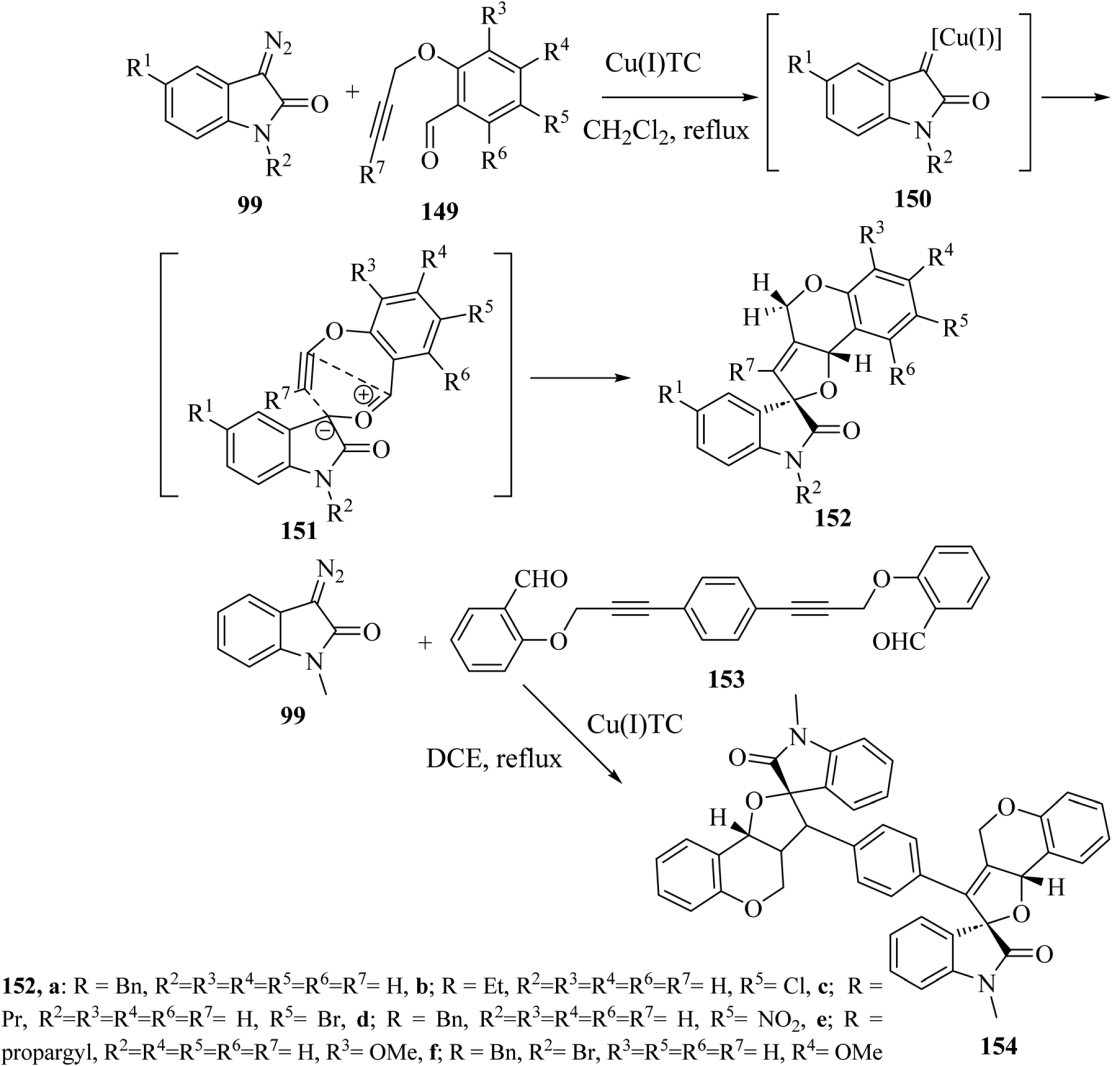
Intramolecular cycloaddition of the indolic carbonyl ylide 151.

Addition of 3-diazoisatins 99 to *O*-propargyl salicylaldehydes 149 in the presence of copper(i) thiophenecarboxylate [(Cu(i)TC] in dichloromethane (CH_2_Cl_2_) as solvent gave the spiro(furo[3,2-*c*]chromene)-2-oxindoles 152 (ref. 88) (Scheme 39). Mechanistically, the reaction between 3-copper(i)carbene-diazoisatins and salicylaldehydes involved the generation of the carbonyl ylide intermediates 151, whose subsequent stereoselective intramolecular cycloaddition gave products 152 in 61–84% yield as single diastereoisomers (Scheme 39).^88^ In the same reaction conditions, a bis-propargylated salicylaldehyde 153 reacted with diazoisatin 99 giving the complex bis-cycloadduct 154 as single diastereoisomer.^88^

**Scheme 40:**
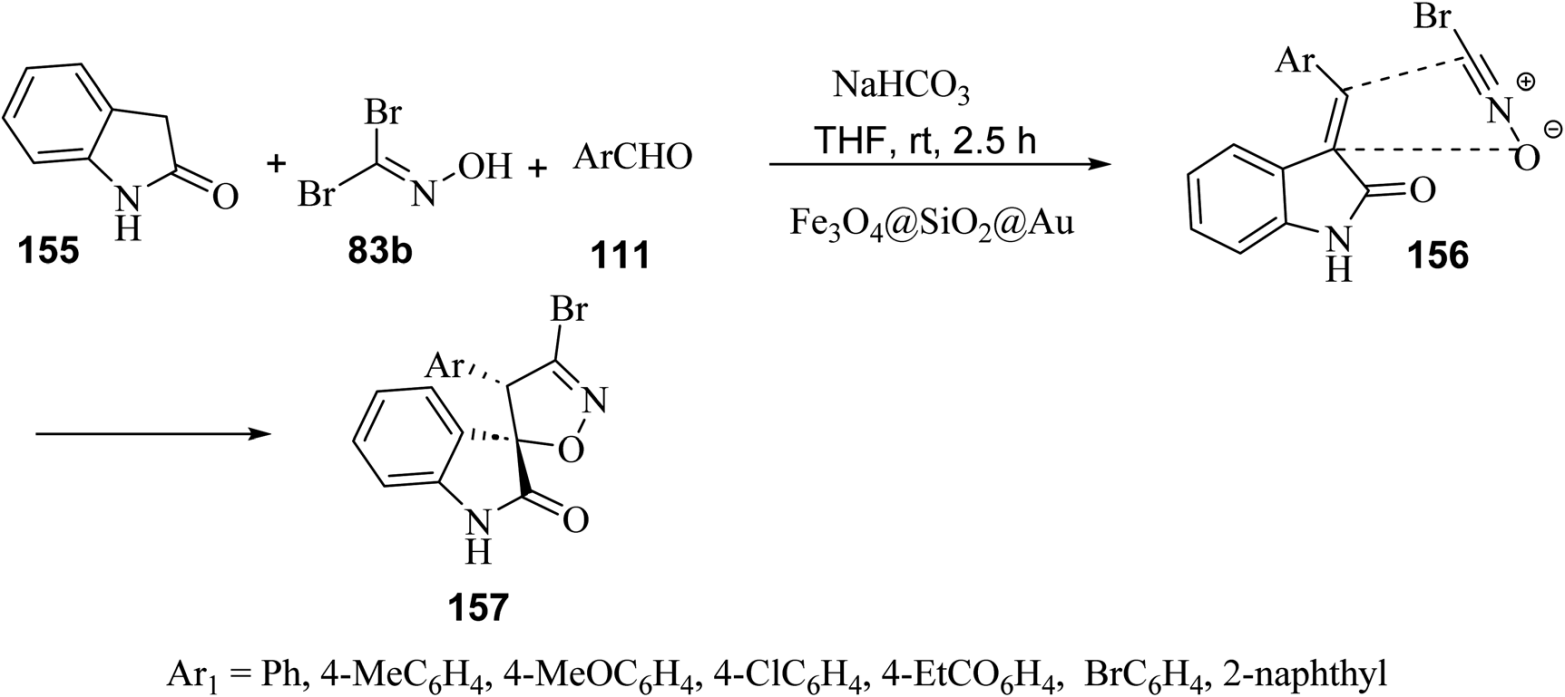
Fe_3_O_4_@SiO_2_@Au-catalyzed three component reactions between 2-oxindole, aromatic aldehydes and dibromoaldoxime.

A regio- and diastereo-selective three-component reaction between aldehydes 111, dibromoformaldoxime (83b) and 2-oxindole (155) has been pursued in the presence of ferrite-silica nanoparticles decorated with Au(0) nanoparticles (Fe_3_O_4_@SiO_2_@Au) as the nano-catalyst (Scheme 40). From the synthetic standpoint, a sequential Knoevenagel condensation-nitrile oxide cycloaddition led to the formation of spiro(2-oxindolo) isoxazolines 157 under mild reaction conditions in 78–85% yield.^89^ Both bromonitrile oxide and 3-alkylidene-2-oxindole, were generated *in situ*, the former by action of sodium hydrogen carbonate (NaHCO_3_) on dibromoformaldoxime, the latter by Knoevenagel condensation between 2-oxindole and aromatic aldehydes. The spiro(2-oxindolo) isoxazoline cycloadducts apparently result from the regioselective attack of bromonitrile oxide on the carbon–carbon double bond of the 3-alkylidene-2-oxindole. From the mechanistic standpoint, Au(0) nanoparticles act as efficient catalysts by activating bromonitrile oxide through the lanthanide contraction effect.^90^

**Scheme 41:**
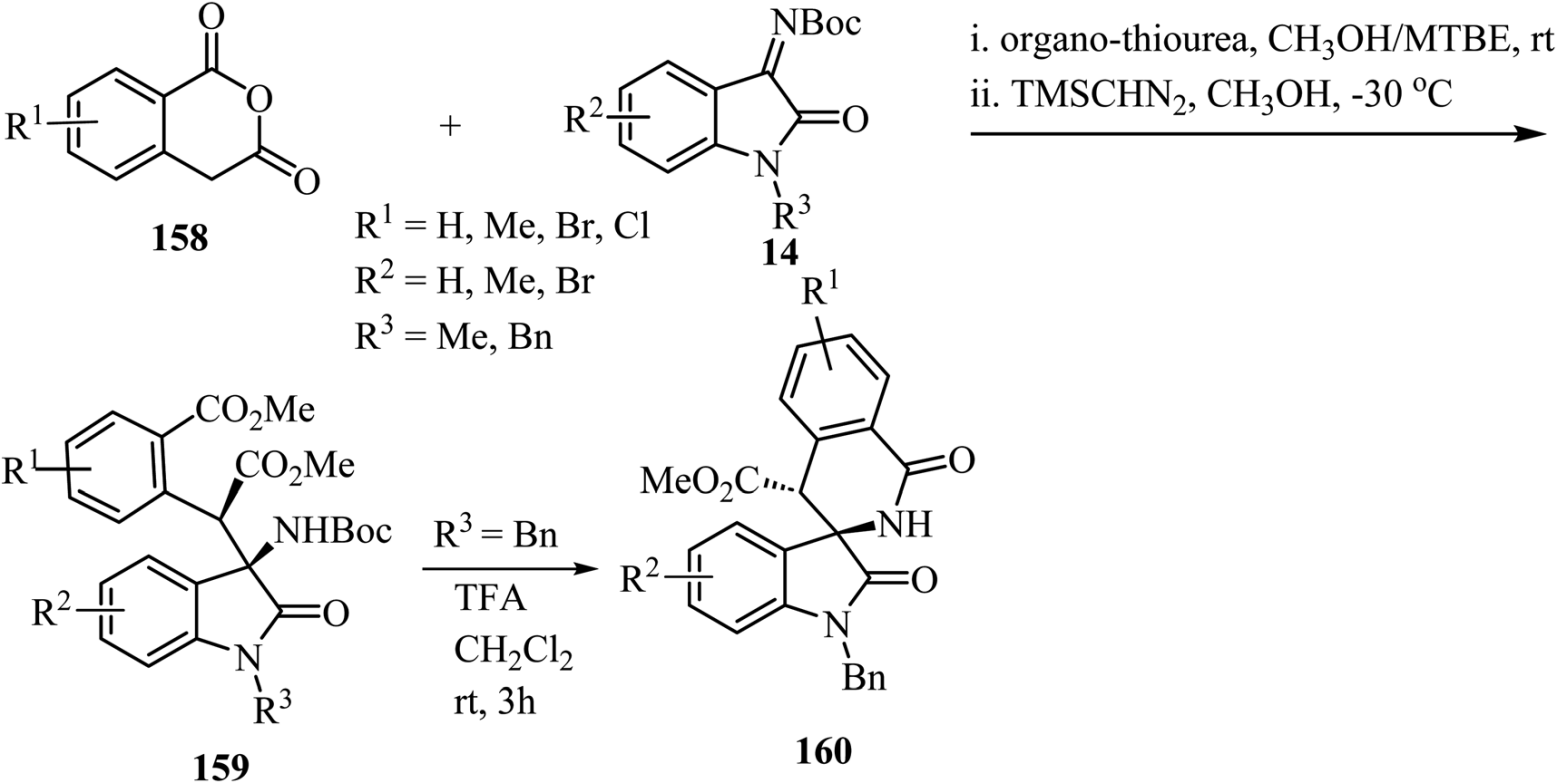
TFA-mediated *N*-Boc deprotection/intramolecular *N*-acylation reaction of oxoindole-derived *α*-aryl-*β*-amino acids.

The synthesis of spirooxindole-*δ*-lactams from oxoindole-derived *α*-aryl-*β*-amino acids has been described.^91^ Oxoindole derivatives 159 were obtained stereoselectively by an organocatalyzed asymmetric Mannich reaction between homophthalic anhydrides 158 and isatin-derived *N*-Boc imines 14 with trimethylsilyldiazomethane (TMSCHN_2_) as methylating agent (Scheme 41). Treatment of oxindoles 159 with trifluoroacetic acid (TFA) provided spiro-*δ*-lactams 160 in good yields (65–75%) with retention of the stereochemistry of the two adjacent carbon chiral centers. The reaction proceeds through deprotection of the amino group followed by intramolecular *N*-acylation to afford the spiro-*δ*-lactams.^91^

**Scheme 42:**
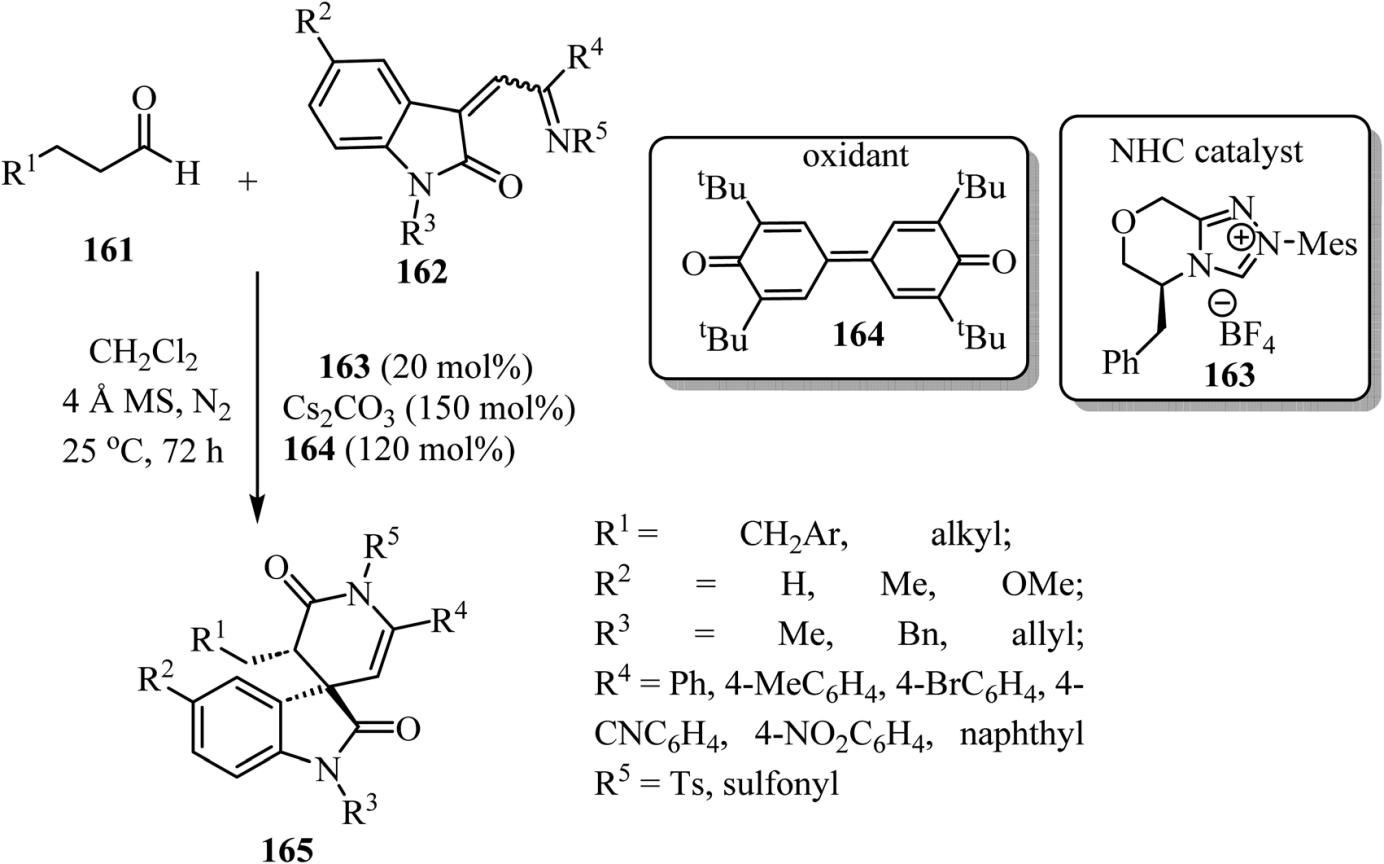
NHC-catalyzed [2 + 4] spiroannulation of aliphatic aldehydes 161 with oxoindole-derived *α*,*β*-unsaturated imines 162.

Spiro-oxindole-*δ*-lactams 165 were synthesized *via* an NHC-catalyzed oxidative [4 + 2] annulation of aliphatic aldehydes 161 with oxindole-derived *α*,*β*-unsaturated imines 162 using chiral pre-NHC catalyst 163 (Scheme 42).^92^ The target spirocyclic lactams 165 were obtained in good yields (up to 94%) and good to excellent enantioselectivities (87–97% ee). The reaction exhibited good functional group tolerance although attempts to carry out the reaction with *N*-Boc-imine or *N*-Ac-imine derivatives did not lead to the formation of the desired products.^92^

**Scheme 43:**
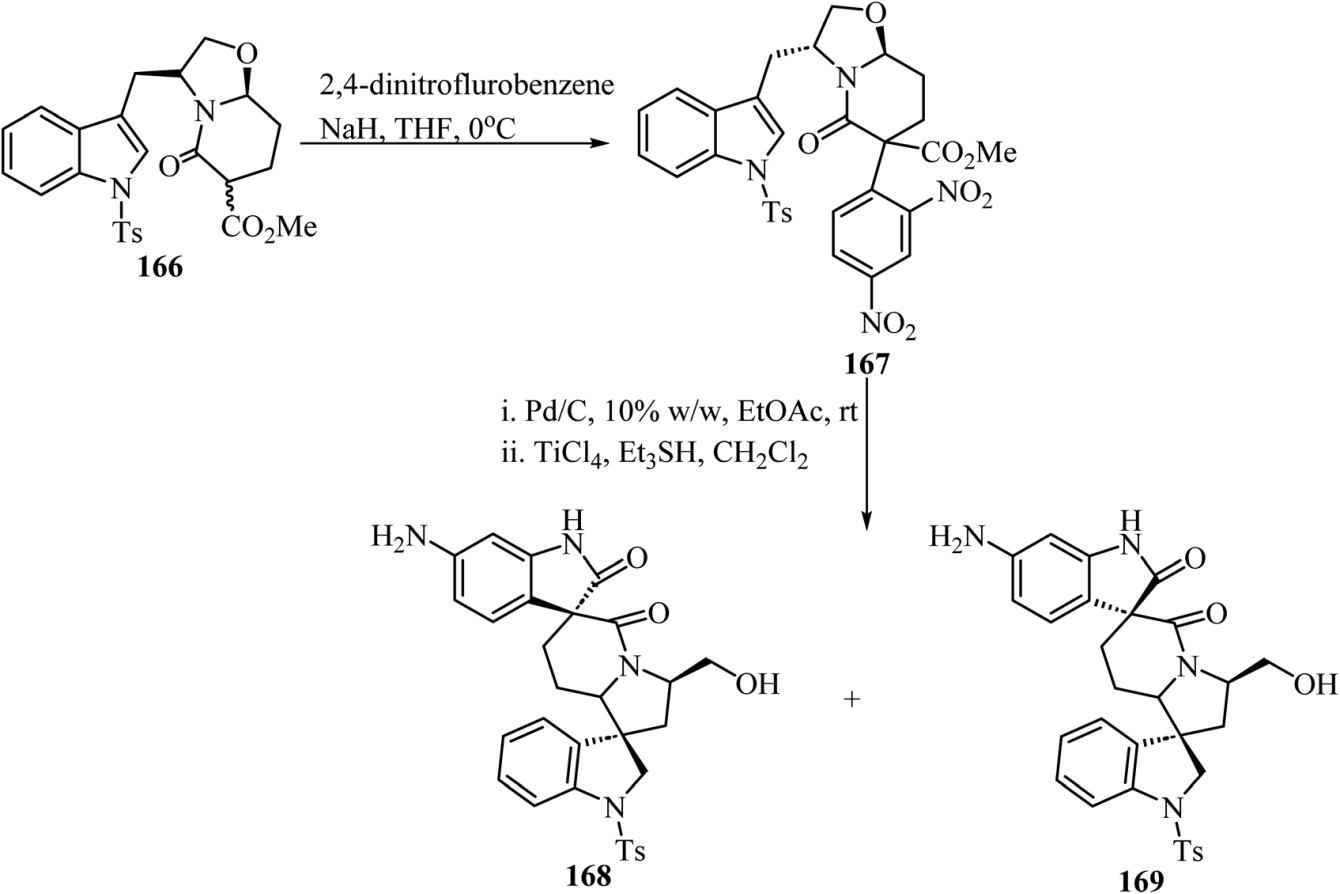
Cyclization of a *S*-tyrptophanol derived chiral bicyclic spiro *δ*-lactam 166.

The synthesis of chiral spiro-*δ*-lactams with antimalarial activity was reported *via* two different strategies using bicyclic *δ*-lactam 166, derived from *S*-tryptophanol, as building block (Scheme 43).^93^ Compound 166 reacted with 2,4-dinitrofluorobenzene *via* aromatic nucleophilic substitution to afford compound 167. Next, reduction with H_2_/Pd–C followed by TiCl_4_/triethylsilyl hydride promoted spiro-cyclization led to spiro oxindole-*δ*-lactams 168 and 169 (Scheme 43).

**Scheme 44:**
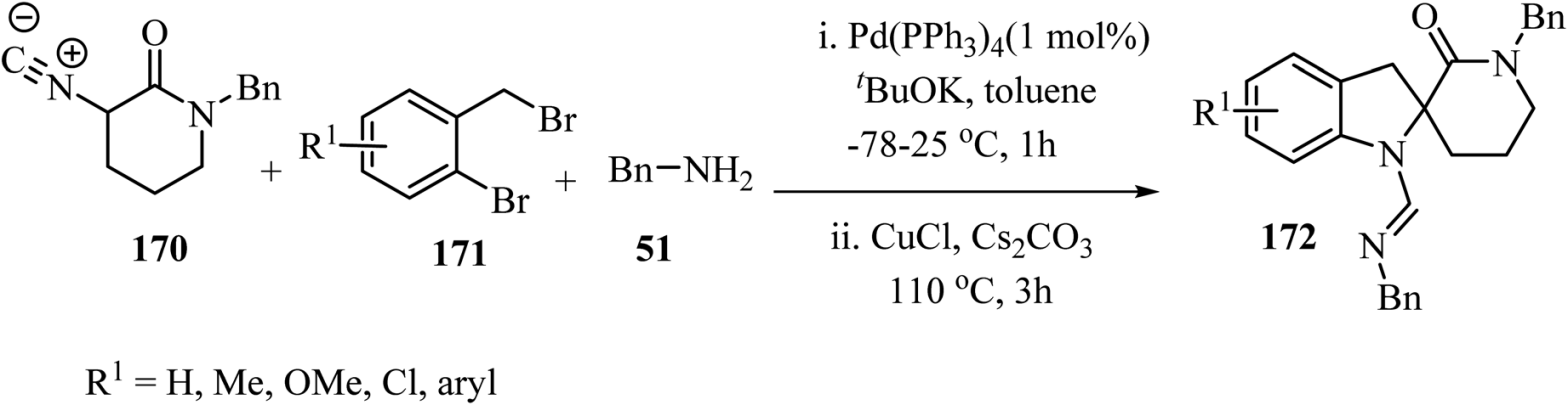
Cooperative Pd/Cu-catalyzed tandem three-component reaction involving *δ*-lactams 170, amines 51 and 2-bromobenzyl bromides 171.

The three-component approach to the synthesis of spiro indoline *δ*-lactams 172, used 2-bromobenzyl bromides 171 as the third reaction component along with *α*-isocyano *δ*-lactams 170 and benzylamine (51), in the presence of a Pd/Cu catalytic system (Scheme 44).^94^

**Scheme 45:**
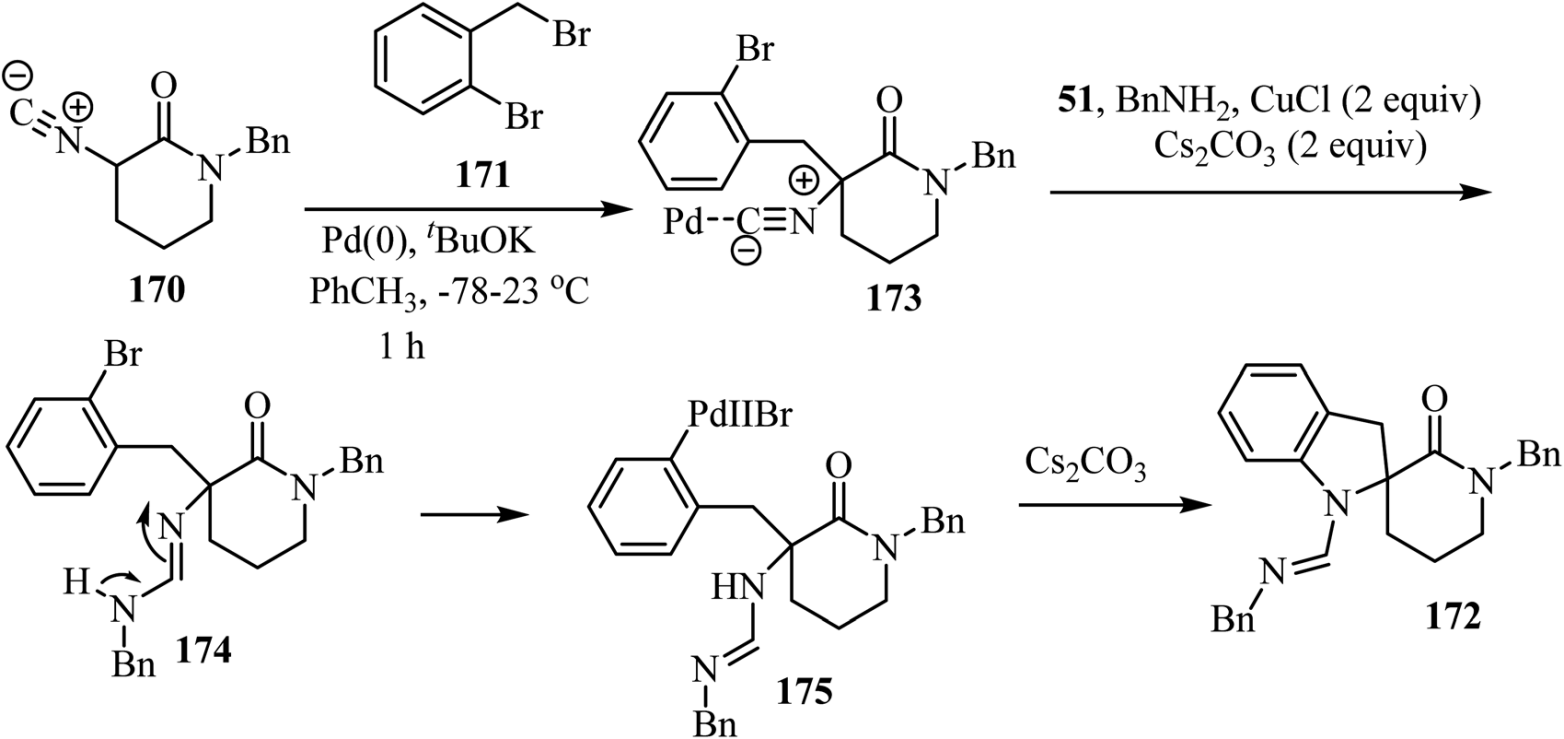
The proposed mechanism for the formation of 172.

The proposed mechanism for this multicomponent reaction involves initial Pd-catalyzed benzylation of *α*-isocyano *δ*-lactams 170 to give intermediate 173 which undergoes a copper-mediated *in situ* amine addition to the isocyanide moiety and finally isomerization to generate 174.^94^ In the final step, the intramolecular *N*-arylation of palladium complex 175*via* Pd/Cu catalysis generates the indoline core, affording spirocyclic *δ*-lactams 172 in moderate to good yields (51–73%) (Scheme 45).^94^

**Scheme 46:**
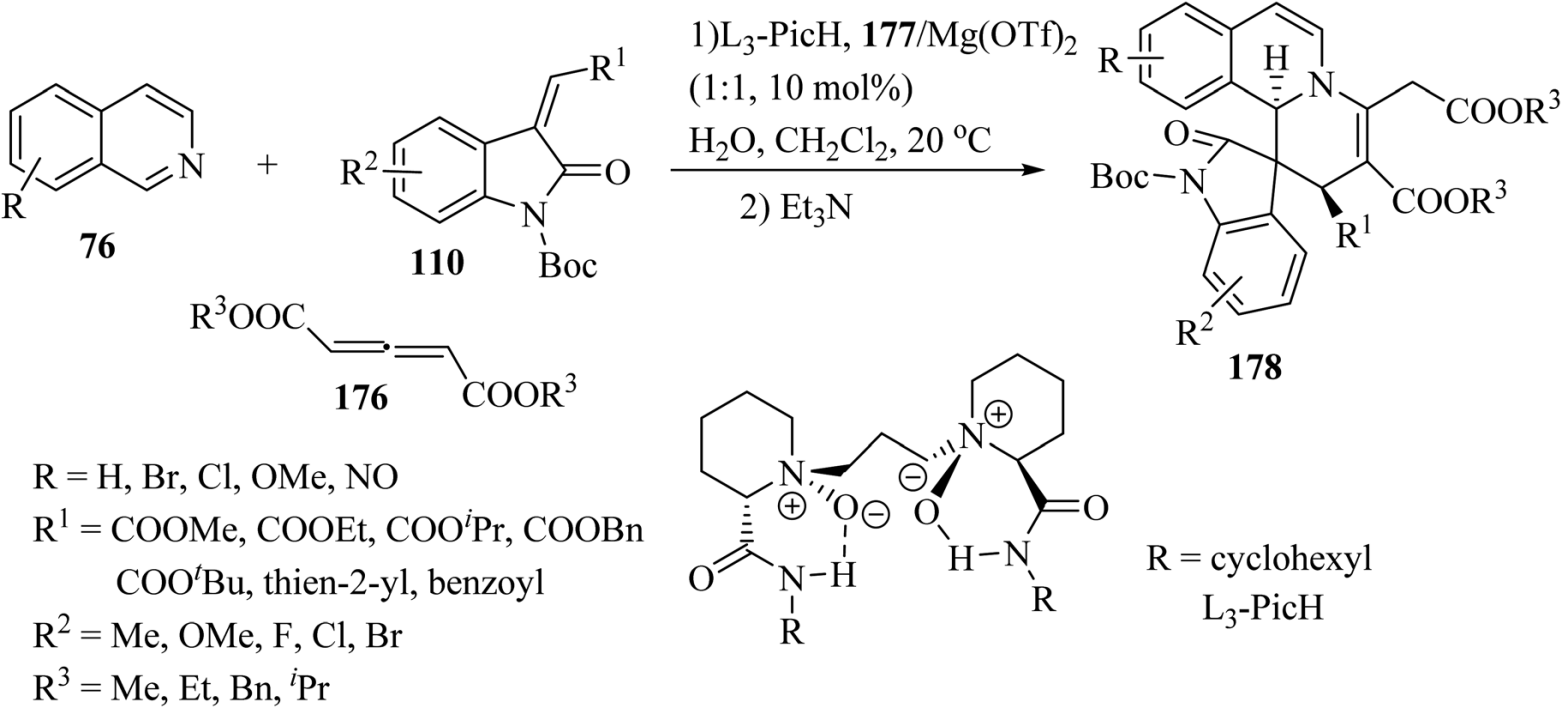
Synthesis of spiro-indolino-pyrido[2,1-*a*]isoquinolines 178.

Recently, an efficient catalytic asymmetric three-component reaction of isoquinolines 76, allene dicarboxylates 176 and methylene indolinones 110 was realized for the synthesis of spiro-indolino-pyrido[2,1-*a*]isoquinolines 178.^95^ In the presence of the chiral *N*,*N*′-dioxide/Mg(OTf)_2_ catalytic system, the reaction proceeded *via* the nucleophilic addition and [4 + 2] cycloaddition/isomerization sequence. The tandem reaction enabled rapid access to the spiro-products with good to excellent stereoselectivities under mild reaction conditions (Scheme 46).

**Scheme 47:**
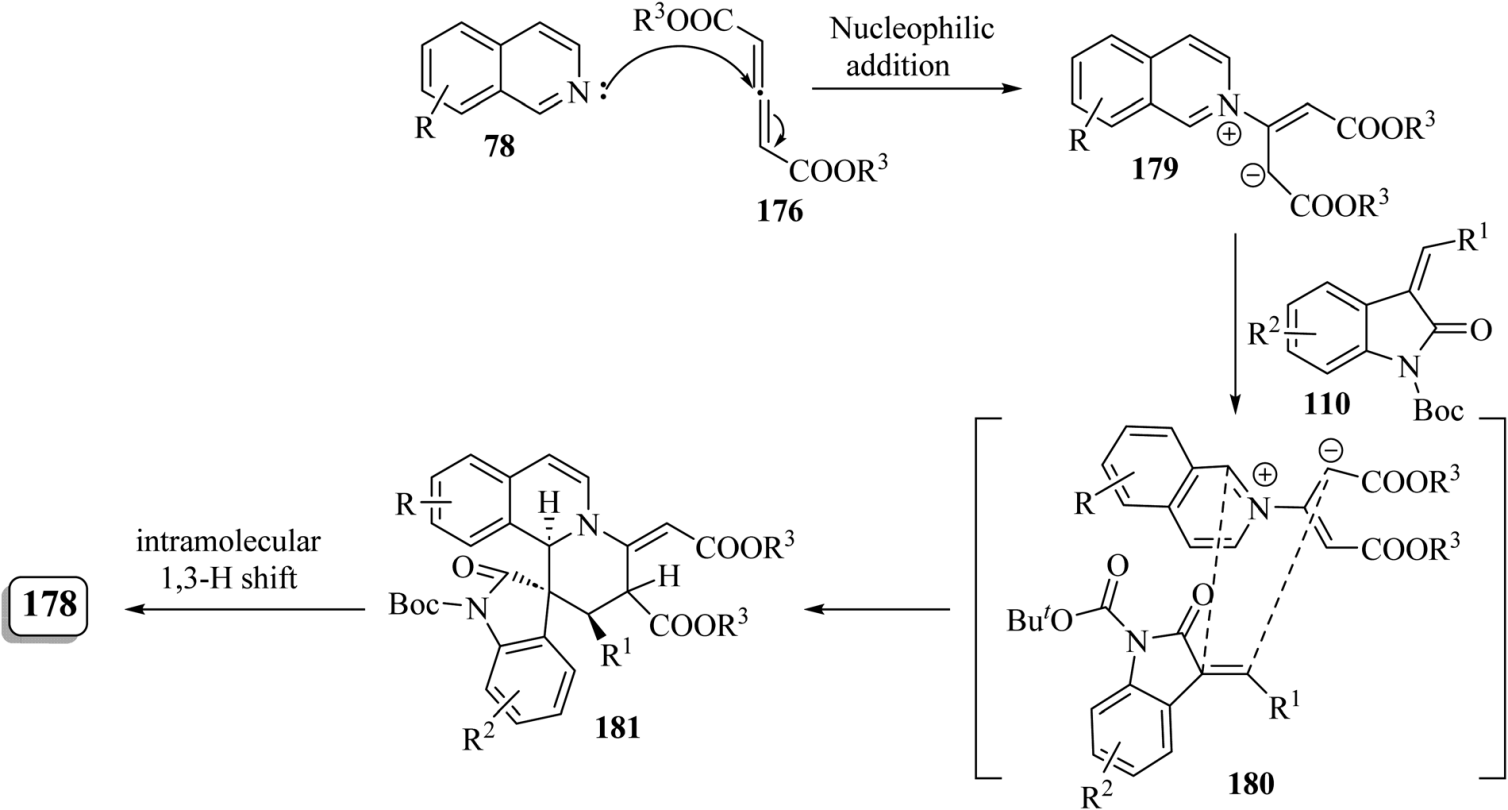
Rational pathway for the formation of 178.

It is conceivable that initially the 1,4-dipole 179 was generated *in situ* through the nucleophilic attack of isoquinoline 78 on the allene dicarboxylate 176 (ref. 95) (Scheme 47). Subsequently, the [4 + 2] cycloaddition of 110 with intermediate 179 proceeded *via* the simultaneous Si/*β-Re* face attack, affording the exocyclic alkene intermediate 180. Finally, intramolecular [1,3]-hydrogen shift resulted in the isomerized product 178 (Scheme 47).^95^

**Scheme 48:**
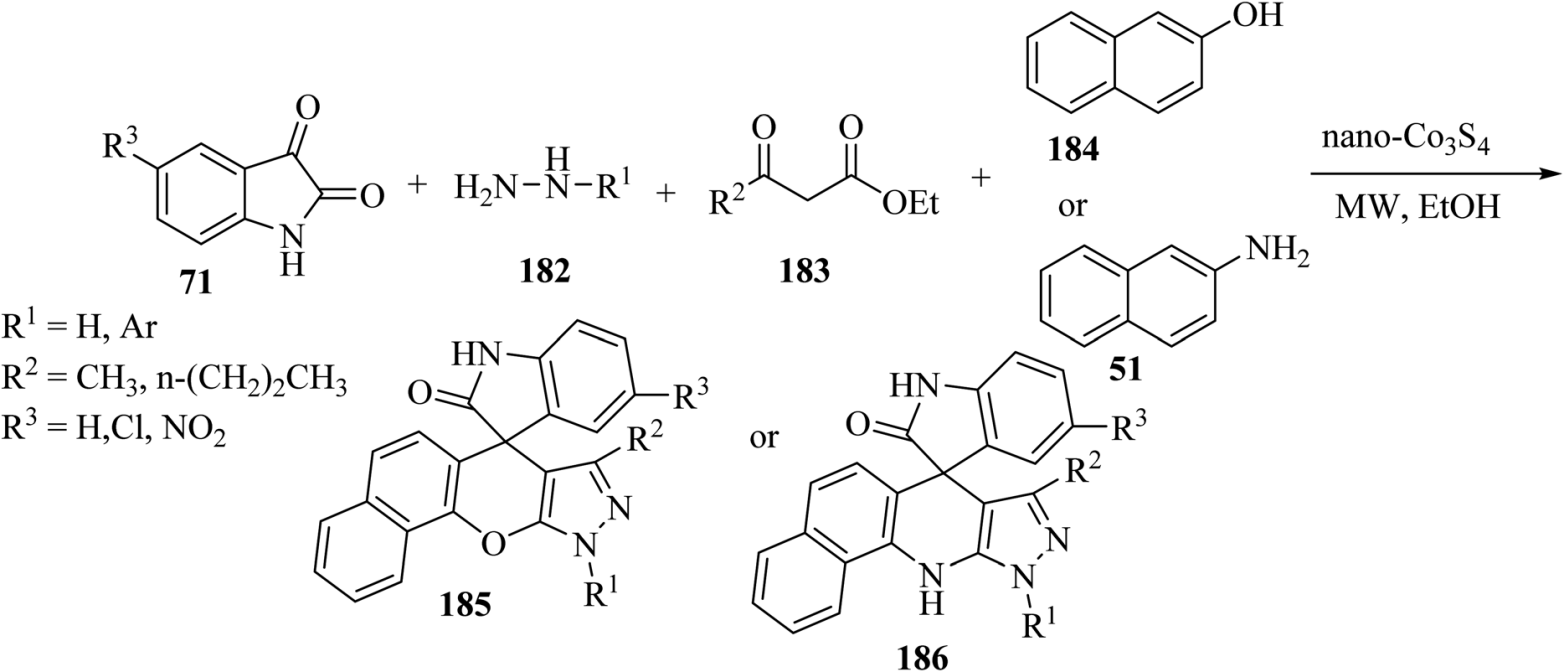
Synthesis of spirooxindoles 185 and 186 under microwave (MW) irradiation.

Khojasteh-Khosro and Shahbazi-Alavi^96^ developed an efficient and rapid procedure for the formation of spiro[benzo[5,6]chromeno[2,3-*c*]pyrazole-11,3′-indol]-2′(1′*H*)-ones 185 and dihydrospiro[pyrazolo-[3,4-*b*]benzo[*H*]quinolin-7,3′-indol]-2′(1′*H*)-ones 186 through a four-component reaction of hydrazines 182, isatins 71, ketoesters 183, and 2-naphthol (184) or naphthylamine (51) in the presence of nano-Co_3_S_4_ under MW-assisted reaction conditions (Scheme 48).^96^

**Scheme 49:**
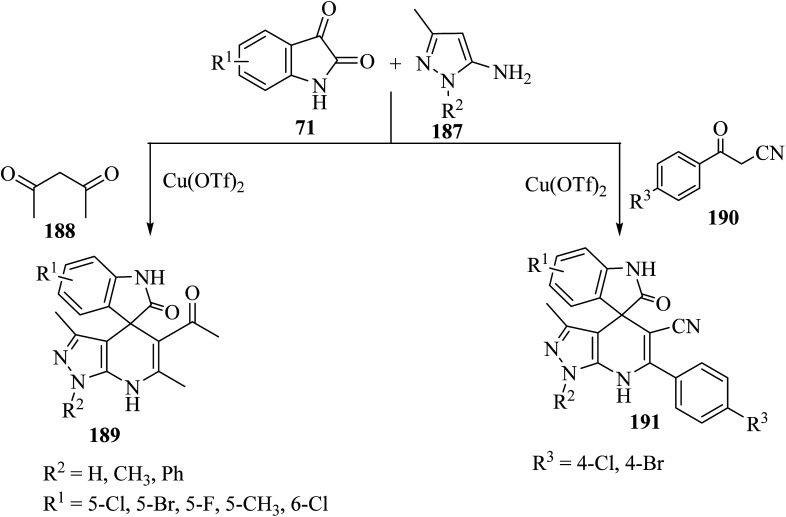
Synthesis of spiro compounds 189 and 191.

Wu *et al.*^97^ have developed a facile, efficient and environmentally benign method to produce the structurally diverse spiro-oxindole scaffolds named spiro[indole-[4*H*]pyrazolo[3,4-*b*]quinolines] 189 and spiro[indoline pyrazolo[3,4-*b*]pyridine]carbonitrile (191) through a three-component condensation of isatin derivatives 71, 5-aminopyrazole (187), and 1,3-dicarbonyl compound such as pentane-2,4-dione (188) or *β*-oxo-benzenepropanenitriles 190 catalyzed by copper triflate [Cu(OTf)_2_] in EtOH (Scheme 49).^97^

**Scheme 50:**
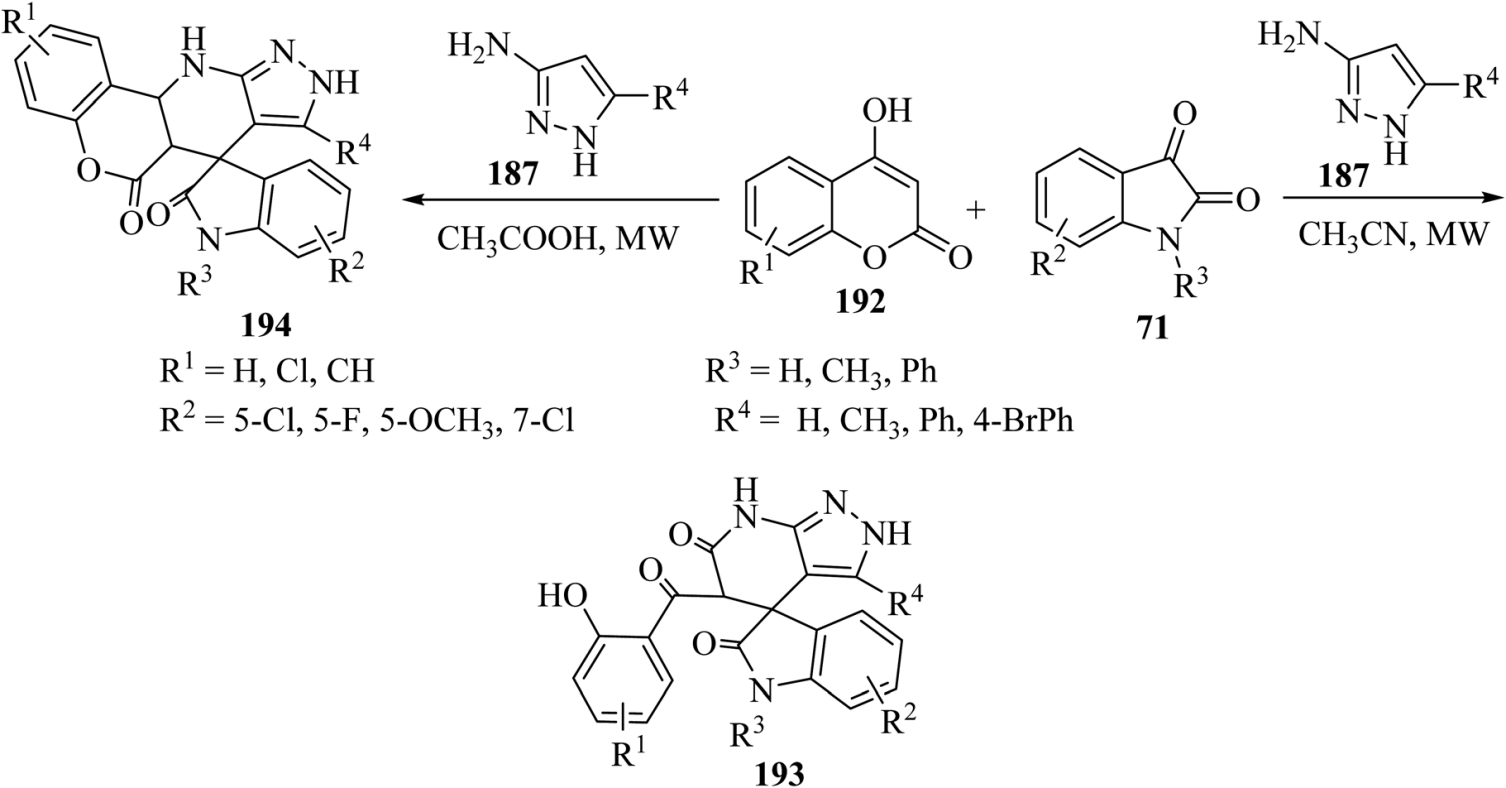
Synthesis of fused spirooxindooles 193 and 194*via* the reaction of 4-hydroxy coumarin derivatives 192 and amino pyrazole 187.

Choudhury and co-workers^98^ have described a medium-dependent, metal-free three-component condensation of isatin derivatives 71, 4-hydroxycoumarin (192) and aminopyrazole (187) under MW-assisted conditions for the formation of two diverse kinds of fused spirooxindoles 193 and 194. Isatin derivatives 71, 4-hydroxycoumarins 192 and aminopyrazole 187 reacted together under MW-assisted conditions in acetonitrile (CH_3_CN) solvent and produced spirooxindoles fused with pyrazolo-tetrahydropyridinones 193*via* opening the ring of the hydroxycoumarin core (Scheme 50). But when acidic conditions are used as the reaction medium, related fused spirooxindoles containing a tetracyclic coumarin dihydropyridine-pyrazole scaffold 194 were obtained. This medium-dependent three component condensation led to the production of a class of pharmaceutically important spiro-oxindoles under metal-free conditions (Scheme 50).^98^

**Scheme 51:**
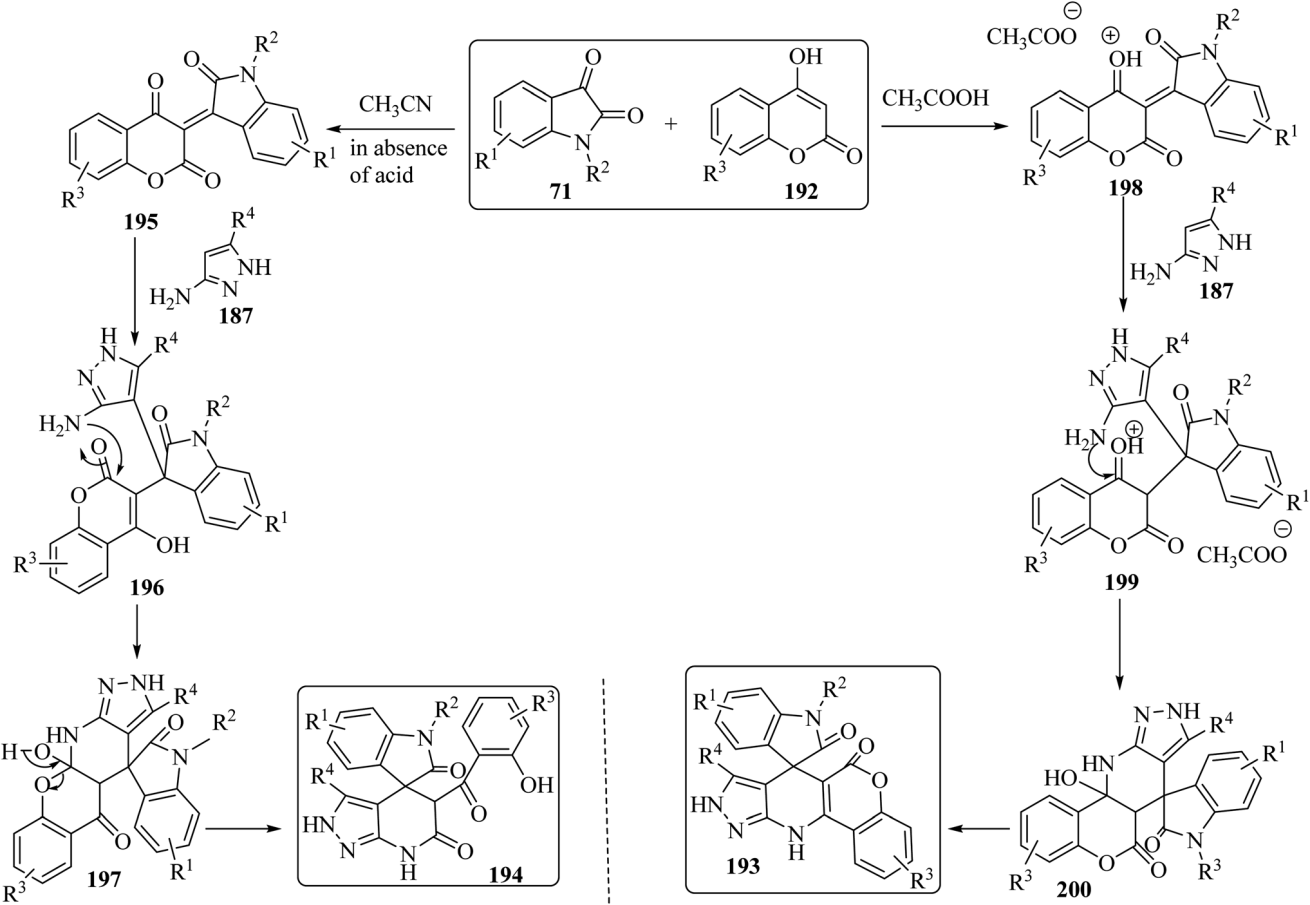
A rational mechanism for the formation of 193 and 194.

The proposed reaction mechanism for the synthesis of 193 or 194 is illustrated in Scheme 51.^98^ It is expected that 4-hydroxycoumarin 192 first reacts with isatin derivatives 71 to form intermediate 195; then amino pyrazole 187 underwent 1,4-addition to obtain tri-substituted methane 196. In non-acidic conditions, 196 remains at the step of enol formation, so the masked carbonyl's reactivity is less than that of the ester part. Therefore, intramolecular cyclization happens in the ester group of the coumarin species and produces intermediate 197, and eventually stable compound 194 forms *via* ring-opening of the coumarin. On the other hand, by using acid, tri-substituted methane intermediate 196 remains as protonated form 199, containing the active protonated carbonyl group (ketone) 200, so ring closure happens intramolecularly by involving the ketone group of the coumarin instead of the ester group, and product 193 was formed (Scheme 51).^98^

**Scheme 52:**
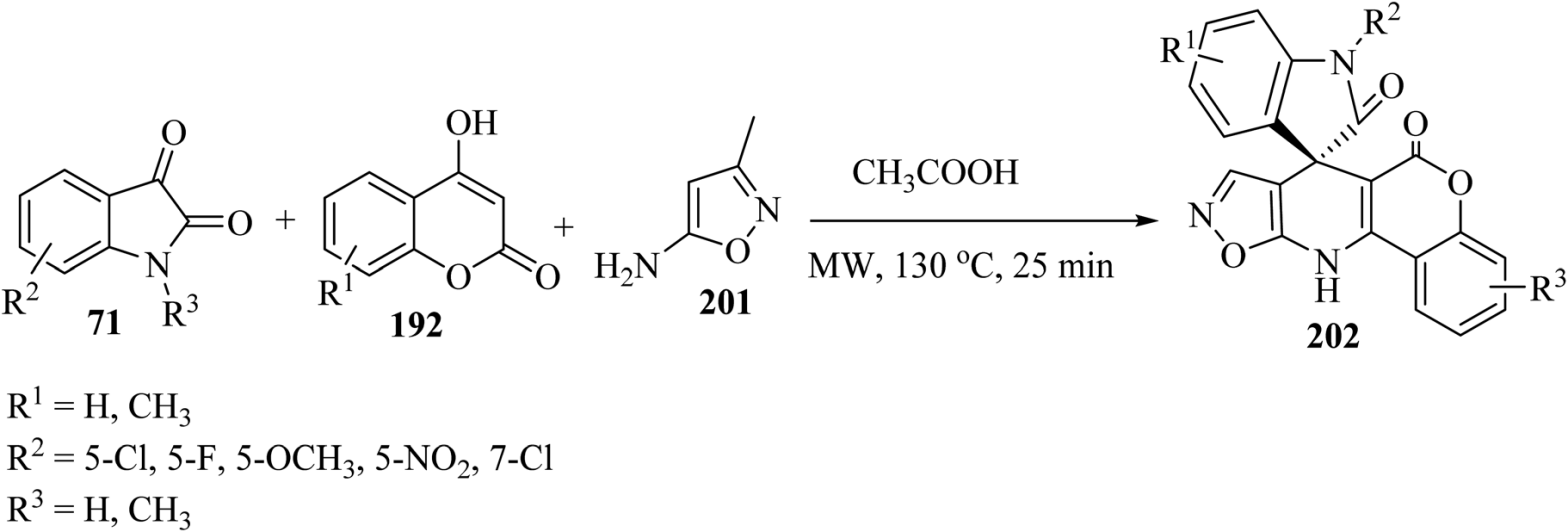
Formation of spirooxindole scaffolds 202 containing coumarin-dihydropyridine-isoxazol tetracycles.

In order to expand the substrate scope, the same authors have prepared another spiro-oxindole fused with coumarin-dihydropyridine-isooxazole tetracycle 202 by applying 5-amino-3-methylisoxazole (201) (Scheme 52).^98^

**Scheme 53:**
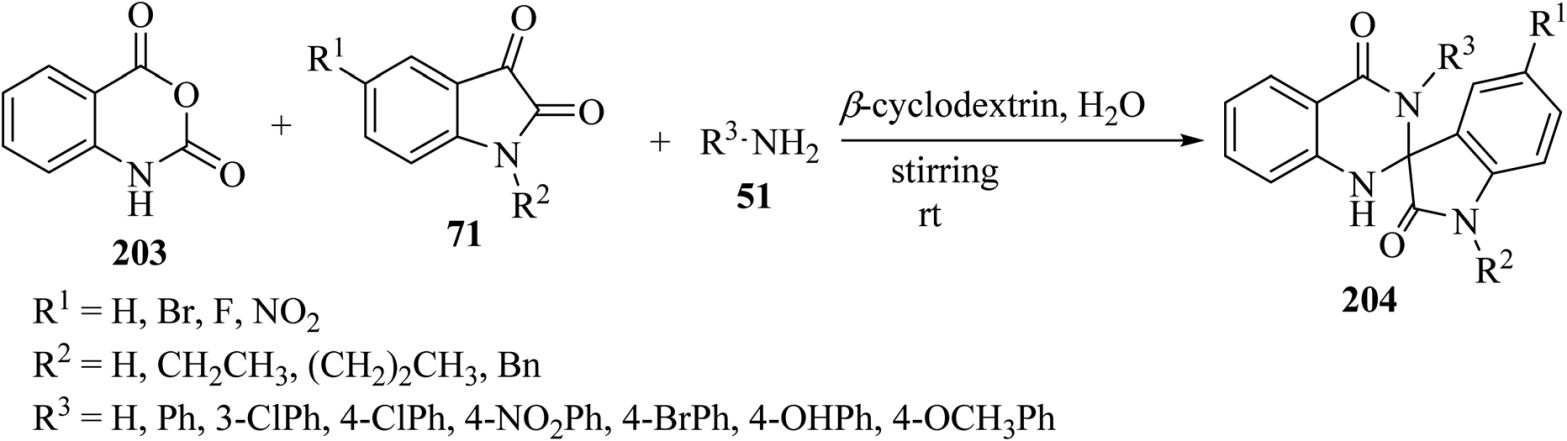
Synthesis of various spiroindole quinazoline derivatives 204.

Tripathi^99^ has introduced an efficient multicomponent synthetic method for the formation of spiro[indoline-3,2′-quinazoline]-2,4′(3′*H*)-diones 204 from isatoic anhydride (203), isatin derivatives 71, and primary amines 51, which was catalyzed by *β*-cyclodextrin in an aqueous medium (Scheme 53). Due to the use of environmentally friendly catalysts and green solvents, this is a green method to produce valuable spiro-heterocycles.^99^

**Scheme 54:**
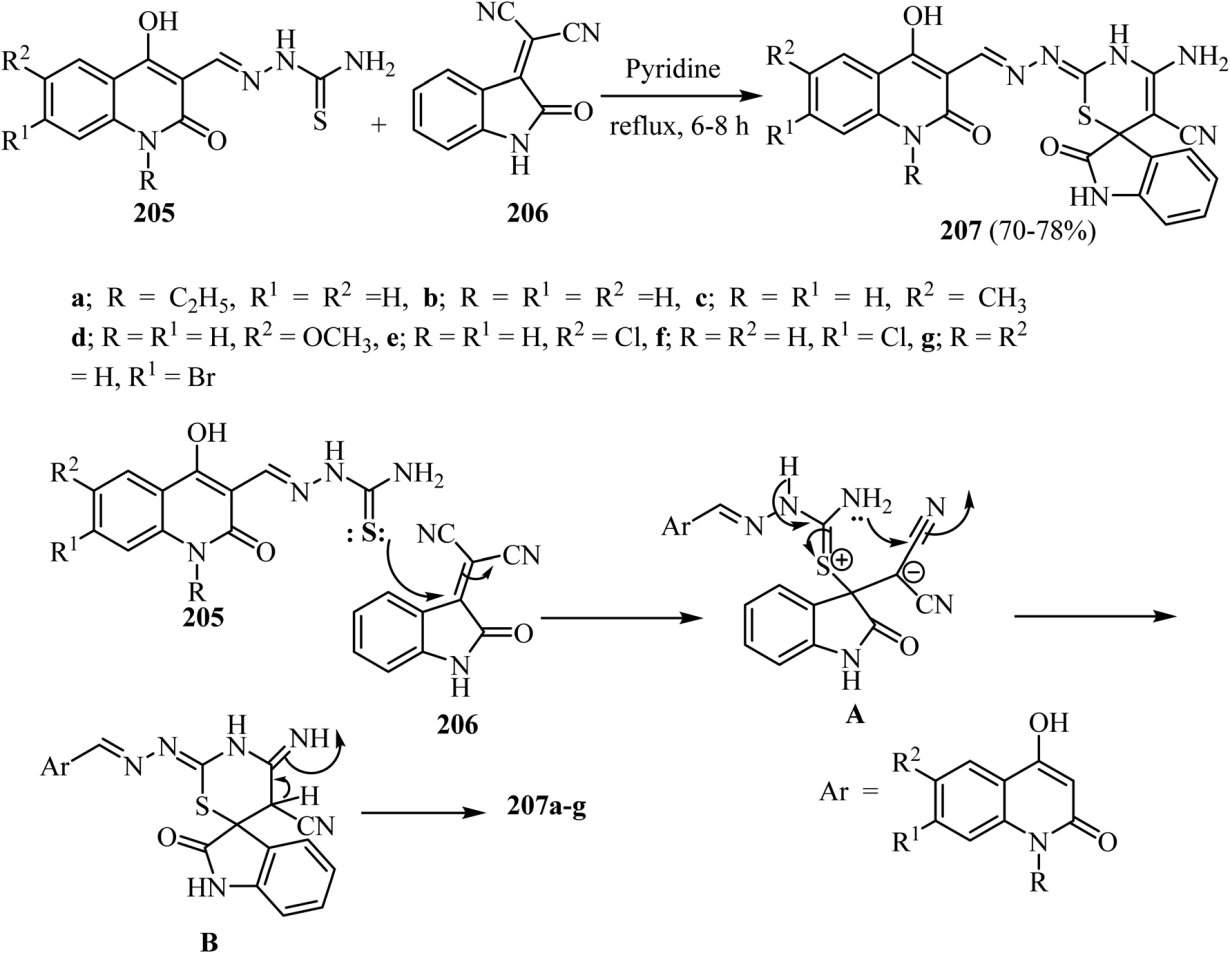
Formation of spiro [indoline-3,6′-[1,3]thiazine]-5′-carbonitriles 207a–g and the mechanism describes their formation.

**Scheme 55:**
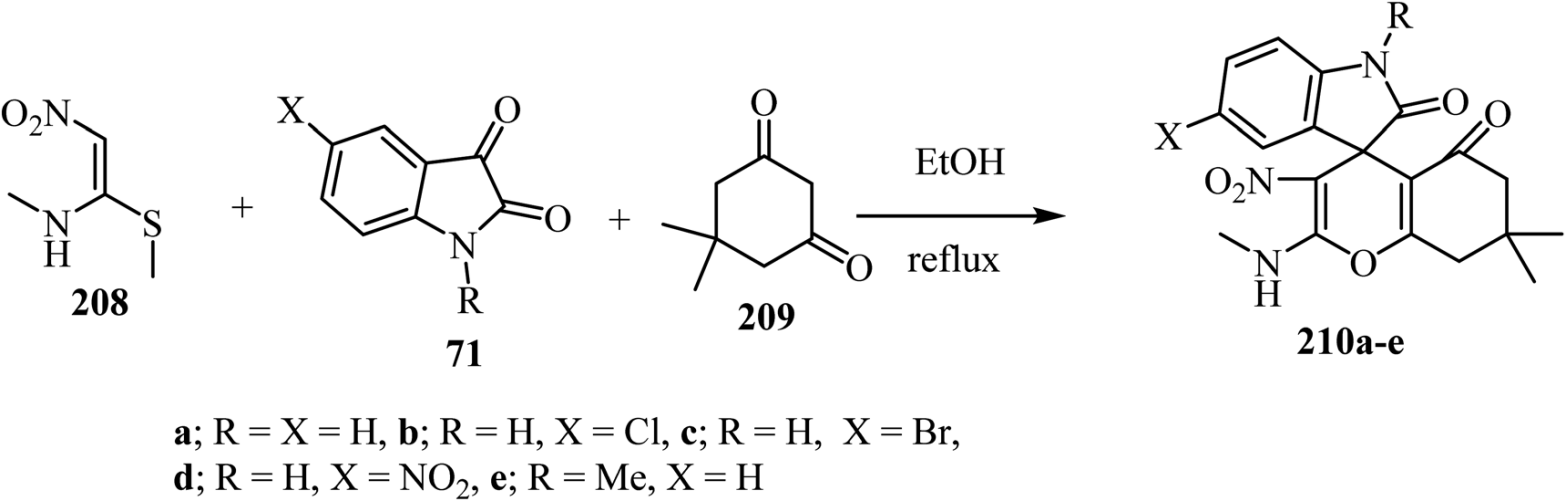
Utility of NMSM for the one-pot synthesis of fused spiro 4*H*-pyrans 210.

Aly *et al.*^100^ prepared a series of spiro[indoline-3,6′-[1,3]thiazines] 207 in modest yields by refluxing substituted (1,2-dihydroquinolin-3-yl)methylene)hydrazine-carbothioamides 205 with 2-(2-oxoindolin-3-ylidene)malononitrile (206) in pyridine as a solvent (Scheme 54).^100^ The suggested mechanism for the formation of spiro indolothiazines 207 was based upon attack by the thione-lone pair in 205 on the olefinic C=C in 206 leading to the formation of salt A as an intermediate (Scheme 53). Attack of the amino lone pair on the electrophilic carbon in the nitrile group leads to the formation of adduct B (Scheme 54). Finally, the products 207a–g were formed after proton transfer in B as shown in Scheme 54.^100^

(*E*)-*N*-Methyl-1-(methylthio)-2-nitroethenamine (NMSM) (208) has drawn significant attention as an important synergistic building block due to the presence of push–pull skeleton for the synthesis of various O/N-heterocyclic ring systems *via* multicomponent reactions (MCRs).^101,102^ By using an MCR strategy and NMSM as a building block, spiro-4*H*-pyrans were synthesized using oxygen containing 1,3-dinucleophilic sources (cyclic-1,3-diketone, 209), isatin derivatives 71, and 1*N*-methyl-1*S*-methyl-2-nitroethylene (NMSM) 208 under catalyst-free conditions to furnish compounds 210 (Scheme 55).^102^ These compounds were initially screened for *in vitro* antibacterial activity against two Gram-positive and three Gram-negative bacterial strains, and all the compounds exhibited moderate to potent antibacterial activity.^102^

**Scheme 56:**
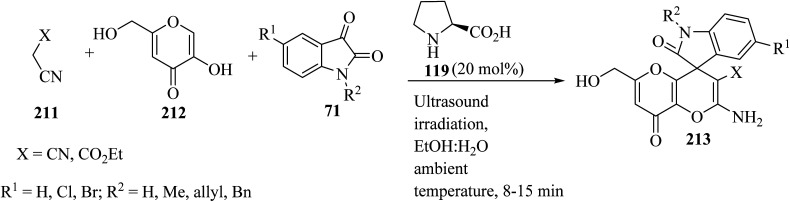
Ultrasound-assisted organocatalytic domino synthesis of spiro[indoline-3,4′-pyrano[3,2-*b*]pyran 213.

Spiro[indoline-3,4′-pyrano[3,2-*b*]pyran]-3′-carbonitrile/carboxylate derivatives 213 were formed from a domino three-component reaction of active methylene compounds 211, kojic acid 212, and isatin derivatives 71 in aqueous ethanolic solution by employing secondary amine, l-proline (119), as a catalyst at ambient temperature (Scheme 56).^103^

**Scheme 57:**
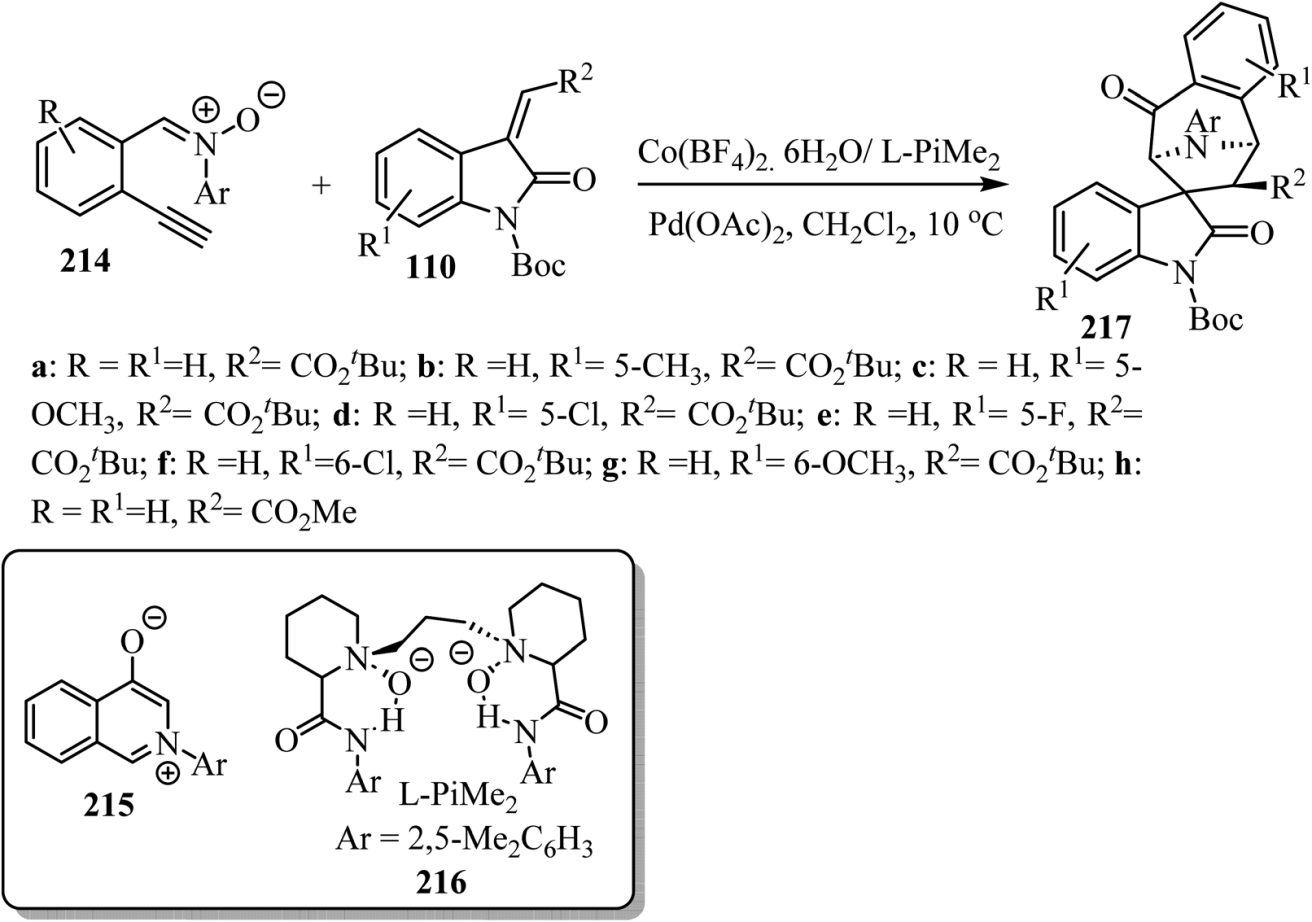
Synthesis of spiro-tropanyl oxindoles 217.

An asymmetric tandem cycloisomerization and intramolecular [5 + 2] cycloaddition reaction of 2-ethynylphenyl substituted nitrones 214 with arylideneindolinones 110 by dual metallic relay catalysis was investigated by Feng's group (Scheme 57). The reaction comprises the palladium(ii)-promoted *in situ* formation of the isoquinolinium salt 215 followed by the chiral *N*,*N*′-dioxide-Co(ii) complex-catalyzed regio-, diastereo-, and enantio-selective [5 + 2] cycloaddition. The desired spiro-tropanyl oxindoles 217 (ref. 104) containing four contiguous chiral centers were produced in good yields. Generally, arylideneindolinones 110 bearing either electron-donating or electron-withdrawing groups at the 5- or 6-position provided good yields. Furthermore, electron-donating groups led to better diastereoselectivity. The synthetic potential of this catalytic system was confirmed by the gram-scale production of the products (Scheme 57).^104^

### 2.4. Synthesis of spiro pyran

**Scheme 58:**
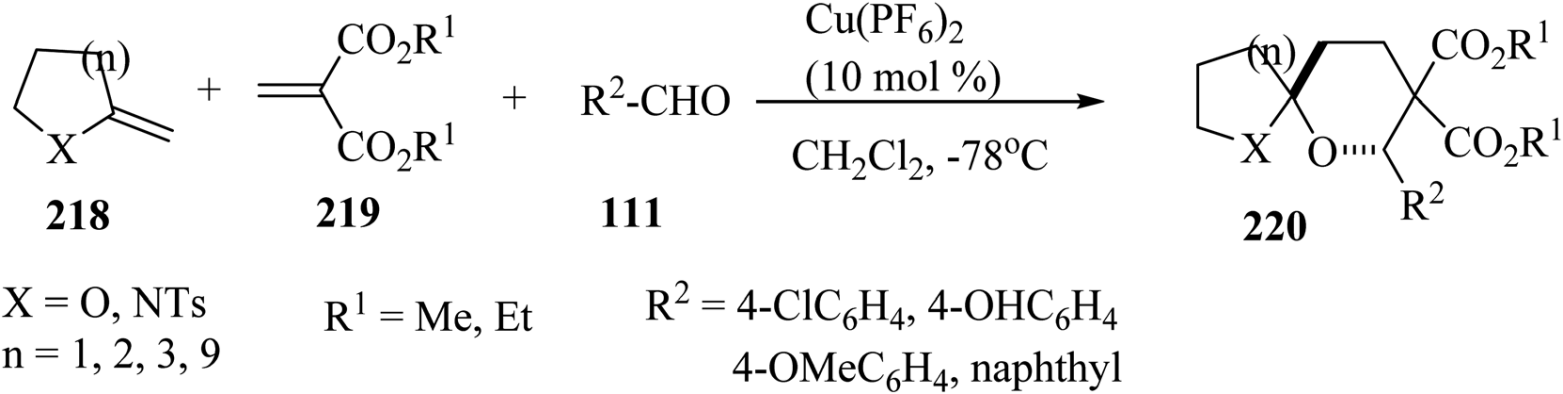
Synthesis of spiroketals 220.

Spiroketals 220 were synthesized in an excellent yield by the reaction of exocyclic enol ether 218, methylene malonate (219), and aldehydes 111. The reaction was carried out with 10 mol% of Cu(PF_6_)_2_ as a catalyst in dichloromethane (CH_2_Cl_2_) at −78 °C (Scheme 58).^105^

**Scheme 59:**
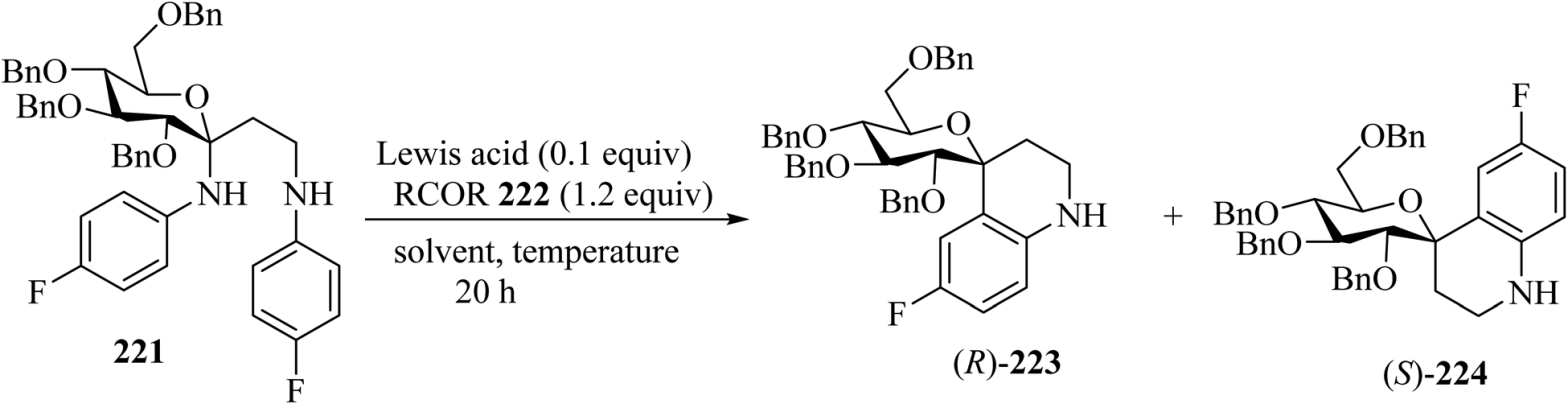
Conversion of *C*,*N*-glycosides to spiro[pyran-4-quinolines] (*R*)-223 and (*S*)-224.

Mixing of *C*,*N*-glucoside 221 with benzophenones 222 in the presence of InCl_3_ (0.1 equiv.) in dichloroethane (DCE) at 60 °C for 20 h resulted in the formation of spiro[pyran-4-quinoline] 223/224 in 59% yield as a mixture of diastereomers (ratio of (*R*)-223/(*S*)-224 = 2.5/1). The reaction did not occur in the absence of benzophenone, indicating that the ketone is essential for promoting the elimination of *N*-aryl group (Scheme 59).^106^

**Scheme 60:**
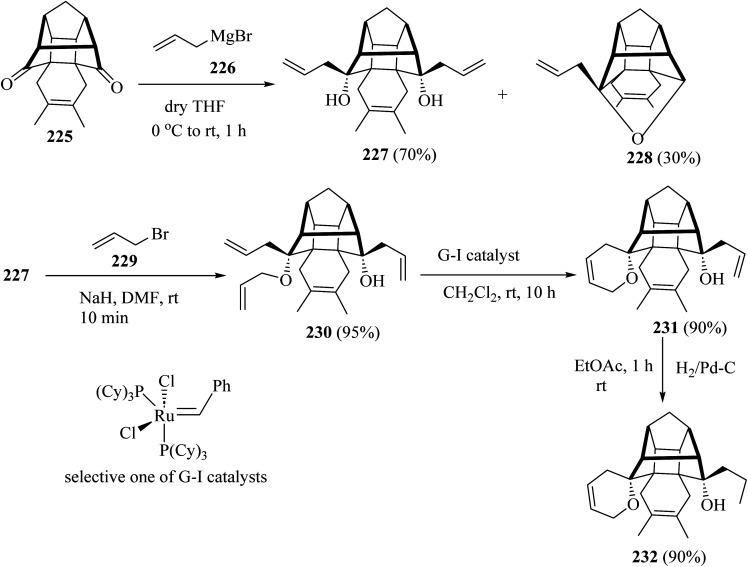
Synthesis of spiro-pyrano-cage framework 231 and 232.

For the synthesis of compound 231, the cage dione 225 was treated with allyl magnesium bromide (226) in dry ether to deliver the diallyl cage diol 227 along with another hemiketal derivative 228 by transannular cyclization. Next, the cage diol 227, on allylation with the NaH in the presence of allyl bromide (229) in dry DMF, gave the triallyl cage compound 230 (ref. 107) (Scheme 60). Subsequent, ring-closing metathesis of triallyl compound 230 with the Grubbs catalysts (or G-I catalysts) produced at room temperature condition, the cage derivative 231 (ref. 107) (Scheme 60). Grubbs catalysts^108^ are a series of transition metal carbene complexes used as catalysts for olefin metathesis. Finally, hydrogenation of the compound 231 with hydrogen in the presence of 10% palladium on activated charcoal in dry EtOAc gave the saturated cage system 232 with a 90% yield (Scheme 60).^107^

**Scheme 61:**
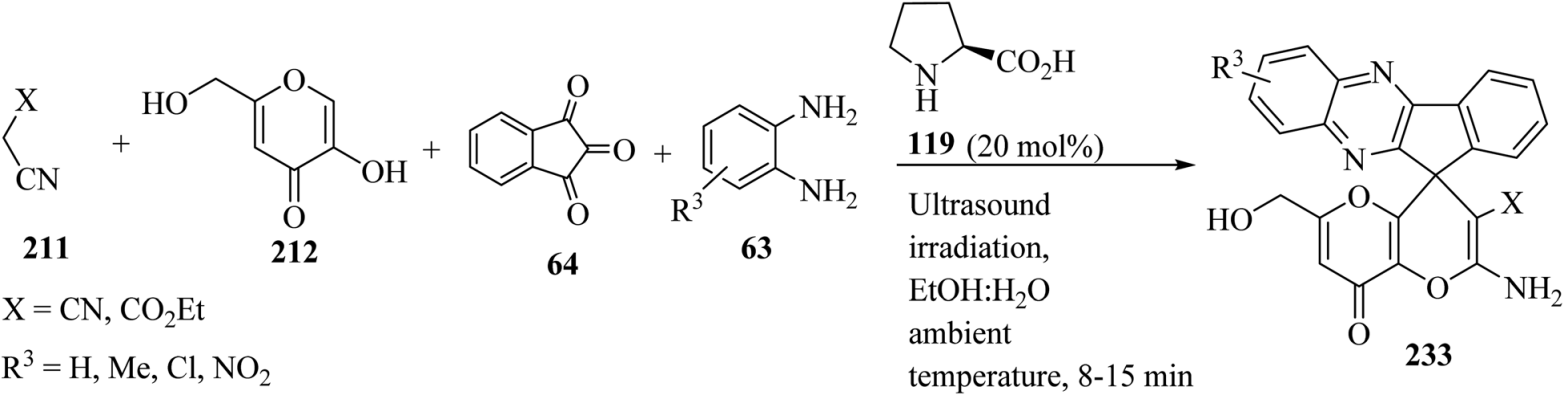
Ultrasound-assisted organocatalytic domino synthesis of 2′-amino-6′-(hydroxymethyl)-8′-oxo-8′*H*-spiro[indeno[1,2-*b*]quinoxaline-11,4′-pyrano[3,2-*b*]pyran]-3′-carbonitrile/carboxylate derivatives 233.

Several amino-substituted 2′-amino-6′-(hydroxymethyl)-8′-oxo-8′*H*-spiro[indeno[1,2-*b*]-quinoxaline-11,4′-pyrano[3,2-*b*]pyran]-3′-carbonitrile/carboxylate derivatives 233 were synthesized *via* ultrasound-assisted organocatalytic domino, four-component reactions of active methylene compounds 211, kojic acid (212), ninhydrin (64), and 1,2-diamines 63 in the presence of l-proline (119) in aqueous ethanolic solution at ambient temperature (Scheme 61).^103^

**Scheme 62:**
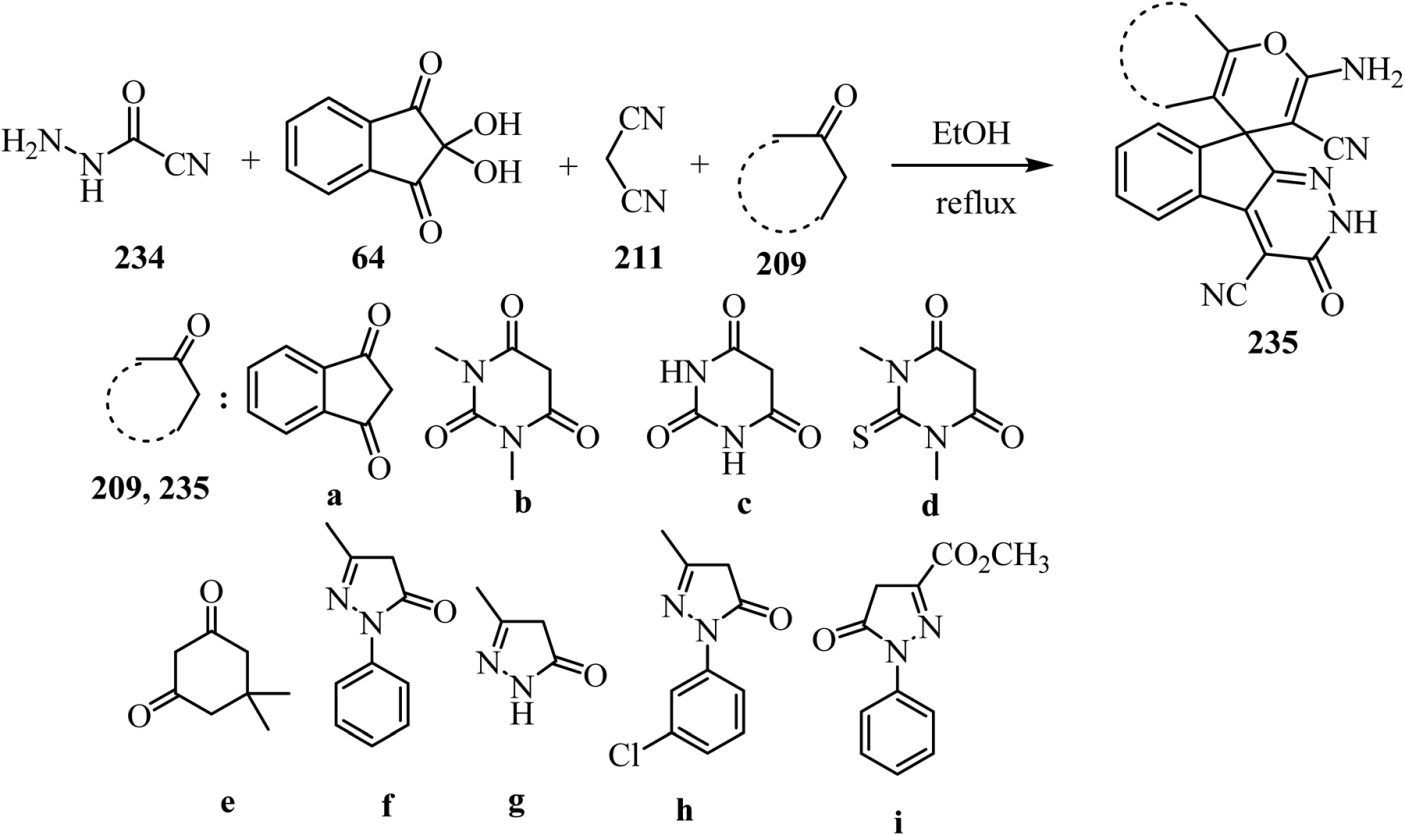
Synthesis of spiroindenopyridazine-4*H*-pyran derivatives 235a–i.

As shown in Scheme 62, spiro[indeno[2,1-*c*]pyridazine-9,4′-pyran]-3′,4′-dicarbonitrile derivatives 235 were synthesized by refluxing a mixture of cyanoacetohydrazide (234), ninhydrin (64), malononitrile (211) and various cyclic CH-acids 209 in EtOH under catalyst-free conditions in a one-pot procedure (Scheme 62).^109^

**Scheme 63:**
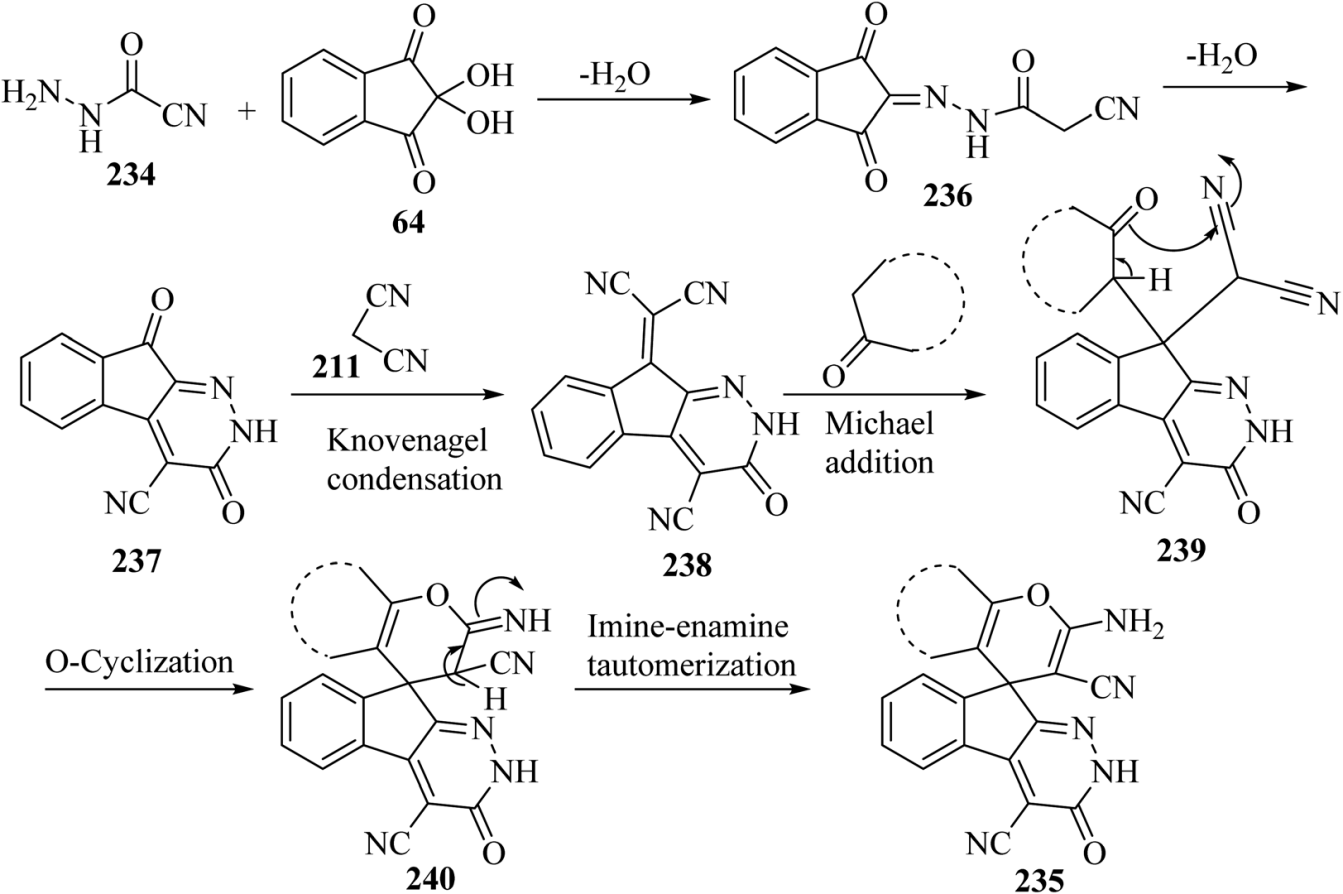
Proposed mechanism for the formation of products 235.

A proposed mechanism for the construction of spiroindenopyridazines 235 is shown in Scheme 63. Initially, condensation of cyanoacetohydrazide 234 and ninhydrin 64 leads to intermediate 236, which undergoes intramolecular cyclization to give the corresponding indeno[2,1-*c*]pyridazine 237. Subsequent addition of malononitrile 211 to indeno[2,1-*c*]pyridazine 237 affords intermediate 238. Michael addition of CH-acid 209 to Knoevenagel adduct 238 leads to intermediate 239, which undergoes keto–enol tautomerization followed by *O*-cyclization *via* nucleophilic addition of oxygen to a nitrile group to produce intermediate 240. Finally, imine–enamine tautomerization of 240 afforded the desired structures 235 ^109^ (Scheme 63).

**Scheme 64:**
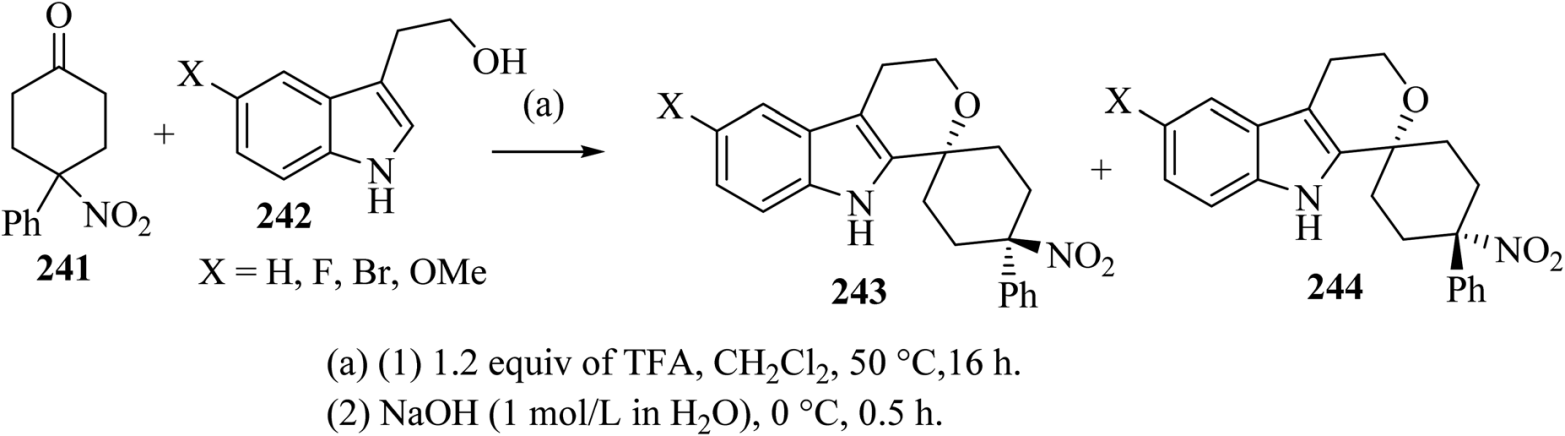
Spirocyclization by oxa-Pictet–Spengler reaction furnishing separable *trans*-isomers 243 and *cis*-isomers 244.

Oxa-Pictet–Spengler reaction between stoichiometric amounts of the nitroketone 241 with the indole derivatives 242 in TFA–CH_2_Cl_2_ proceeded straightforwardly (Scheme 64).^110^ In general, the *trans* diastereoisomers 243 were precipitated from the reaction mixture and were collected by filtration. The supernatants contained the *cis*-diastereo-isomers 244 together with residual amounts of the *trans*-isomers 243, which were separated and purified by chromatography.^110^

**Scheme 65:**
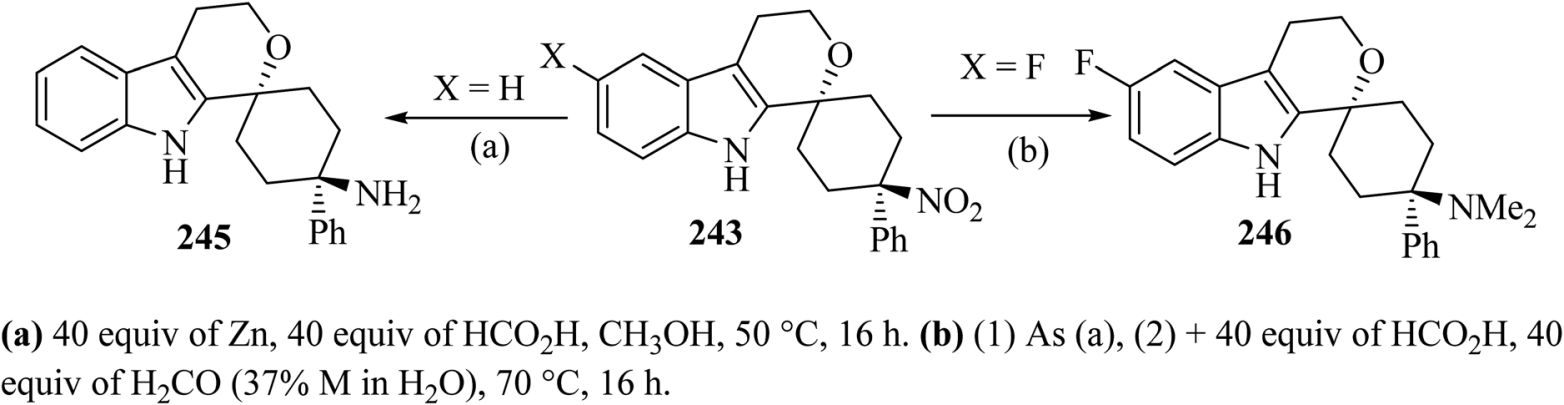
Synthesis of spiro-pyran 246.

Synthesis of spiro-pyran 246 ^111^ was established as shown in Scheme 64. The relative configuration of one representative was established by the *trans*-isomer 243; it was subsequently submitted to the reduction of the nitro group with zinc to furnish the primary amine 245 (89%) (Scheme 65). Formic acid was chosen because it was also used in the next step, the reductive amination with formaldehyde according to an Eschweiler–Clarke protocol,^112^ which gave the corresponding dimethylamino derivative 246.^111^

## 3. Conclusion

This review summarizes various methodologies used to synthesize spiro-azetidine-2-one, spiro-pyrrolidine, spiro-indol(one), and spiro-pyran compounds with enormous scope in pharmaceutics. During the past two decades, ample attention has gone into replacing the age-old methods associated with volatile solvents, harsh reaction conditions, and poor yield of products. New methods have been developed to mitigate these shortcomings as well as increase quantitative yields. In this review, new methods have been systematically catalogued for the convenience of readers. In addition, we focused light on some spots dealing with biological activity of the aforementioned spiro heterocycles.

## Author contributions

M. B. Alshammari (writing and revision), A. A. Aly (conceptualization, writing, edit, revision, and submitting), A. Ahmed (editing), A. H. Mohamed (writing and editing), A. B. Brown (editing). All authors have read and agreed to the published version of the manuscript.

## Conflicts of interest

The authors declare no conflicts of interest.

## Supplementary Material
